# Duration of orthodontic treatment with fixed appliances in adolescents and adults: a systematic review with meta-analysis

**DOI:** 10.1186/s40510-020-00334-4

**Published:** 2020-10-05

**Authors:** Allen Abbing, Vasiliki Koretsi, Theodore Eliades, Spyridon N. Papageorgiou

**Affiliations:** Clinic of Orthodontics and Pediatric Dentistry Center of Dental Medicine, University of Zurich, Plattenstrasse, 11 Zurich, Switzerland

**Keywords:** Orthodontics, Fixed appliances, Treatment duration, Clinical trials, Systematic review, Meta-analysis

## Abstract

**Objectives:**

Adults with fixed orthodontic appliances are increasing nowadays. Compared with adolescents, adults present biological differences that might influence treatment duration. Therefore, the aim of the study was to compare duration of treatment with fixed appliances between adults and adolescents.

**Materials and methods:**

Eight databases were searched up to September 2019 for randomized and non-randomized clinical studies comparing treatment duration with fixed appliances in adolescents and adult patients. After duplicate study selection, data extraction, and risk of bias assessment with the Cochrane ROBINS-I tool, random effects meta-analyses of mean differences (MD) and their 95% confidence intervals (CIs) were performed, followed by assessment of the quality of evidence with GRADE.

**Results:**

A total of 11 unique studies (one prospective and 10 retrospective non-randomized) with 2969 adolescents and 1380 adult patients were finally included. Meta-analysis of 7 studies found no significant difference in the duration of comprehensive treatment with fixed appliances (MD = − 0.8 month; 95% CI = − 4.2 to 2.6 months; *P* = 0.65; *I*^2^ = 92%) between adults and adolescents. Similarly, both distalization of upper first molars with skeletal anchorage for class II correction and the retraction of canines into the premolar extraction spaces lasted similarly long among adults and adolescents. On the other hand, alignment of palatally displaced canines lasted considerably longer in adults compared to adolescents (1 study; MD = 3.8 months; 95% CI = 1.4 to 6.2 months; *P* = 0.002). The quality of evidence for the meta-analysis was low due to the inclusion of non-randomized studies with considerable risk of bias.

**Conclusions:**

While existing evidence does not indicate a difference in the overall duration of treatment with fixed appliances between adults and adolescents, the alignment of palatally displaced canines lasted significantly longer in adults. However, our confidence in these estimates is low due to the risk of bias in the included studies.

**Trial registration:**

PROSPERO: (CRD42019148169)

## Introduction

Over the last several years, there has been an increase in the proportion of adults in orthodontic practices. This is due to the projected modern beauty standards, raised public awareness, increased treatment desire, novel techniques, and extensive direct-to-consumer advertising [1].

However, orthodontic treatment of adult patients might considerably differ from the treatment of children and adolescents. In growing adolescents, many malocclusion traits are corrected by attempting to influence physiological growth with orthopedic appliances [2, 3]. Adult patients do not exhibit growth potential, and they are thus treated with other protocols, which usually focus on dentoalveolar compensation [4]. Due to that fact, orthodontic treatment might differ in expectations, duration, and obtained results in adult patients.

Moreover, orthodontic tooth movement is a primarily biological process initiated by forces, which are translated to biochemical signals, and it is mainly dependent on the physiology of mineralized and non-mineralized tissues [5]. Animal studies imply that biological differences between adult and juvenile rats are apparent during orthodontic tooth movement. Lower initial rates of osteoclast differentiation, absence of a positive correlation between the rate of tooth movement and the number of activated osteoclasts [6], significantly lower proliferation activity of the periodontal ligament cells in the initial phase of tooth movement [7], and a decreased bone turnover activity [8] have been reported in older rats. Although the initial phase of tooth movement appeared to be faster in juvenile than adult rats, tooth movement rates were similar once the linear phase was reached [6]. In human adults, inflammatory mediators of the gingival crevicular fluid were reported to be less responsive in the initial phase of tooth movement [6] and yet higher levels of cytokine and osteoclast activity were coupled with slower tooth movement rates [9].

It is widely accepted that orthodontic treatment lasts for a long time; an average treatment with fixed appliances approximately lasts 24.9 months [10]. Considering that long-treatment times are a burden to the patients and are associated with various adverse effects [11, 12], the ability to predict treatment duration and accordingly inform patients in advance is an essential skill for orthodontists [13] and lies in the interest of both orthodontists and patients. In that context, patients’ age might be an important factor in predicting treatment duration.

## Objective

The present systematic review aims to critically compare the evidence derived from randomized and non-randomized clinical trials on the duration of treatment with fixed appliances between adolescents and adults.

## Materials and methods

### Protocol and registration

This review’s protocol was made a priori, registered in PROSPERO (CRD42019148169), and all post hoc changes were appropriately noted (Appendix 1). This review is conducted and reported according to the Cochrane Handbook [14] and PRISMA statement [15].

### Eligibility criteria

Clinical studies on human patients of any age, sex, ethnicity, or malocclusion were included, in which duration of orthodontic treatment with fixed appliances was compared between adolescent and adult patients (Appendix 2). Due to high inter-individual differences, the cut-off age of adulthood was arbitrarily chosen to be 18 years of age, unless otherwise noted in the included studies. No limitations concerning language, publication year, or status were applied. The primary outcome of this review was the duration of comprehensive orthodontic treatment in months from the insertion to the removal of fixed appliances. The secondary outcome was to assess the complete duration of any partial orthodontic treatments, like alignment of displaced canines or correction of deep-bites/cross-bites, if such treatments were reported.

### Information sources and search

Eight electronic databases were systematically searched without any restrictions for publication date, type, and language from inception up to 28 September 2019 (Appendix 3), while Directory of Open Access Journals, Digital Dissertations, metaRegister of Controlled Trials, WHO, and Google Scholar, as well as the reference lists of eligible articles or existing systematic reviews were manually searched for any additions.

### Study selection

Two authors (AA, SNP) screened the titles and/or abstracts of studies retrieved from the searches to identify articles that potentially meet the inclusion criteria, before moving to their full texts. Any differences between the two reviewers were resolved by discussion with a third author (VK).

### Data collection process and items

Data collection from the identified reports was conducted using pre-defined and piloted forms covering (a) study characteristics (design, clinical setting, country), (b) patient characteristics (age, sex), (c) malocclusion characteristics, (d) appliance characteristics, and (e) number and type of extractions performed (if any). Data were extracted by two authors (AA, SNP) with the aforementioned way to resolve discrepancies.

### Risk of bias of individual studies

The risk of bias of included randomized studies was assessed with the Cochrane Collaboration’s RoB 2.0 tool [16]. The risk of bias of included non-randomized studies was assessed with the ROBINS-I (“Risk Of Bias In Non-randomized Studies of Interventions”) [17]. Assessment of the risk of bias within individual trials was likewise independently performed by two authors (AA, SNP) and discrepancies were resolved by consulting a third author (VK).

### Data synthesis and summary measures

An effort was made to include all existing trials in the analysis; where data were missing, they were calculated by us. As duration of orthodontic treatment is bound to be affected by clinician-, appliance-, and patient-related characteristics, a random-effects model was deemed appropriate to calculate the average distribution of true effects, based on clinical and statistical reasoning [18], and a restricted maximum likelihood random-effects model was used according to recent guidance [19]. Mean differences (MDs) and their corresponding 95% confidence intervals (CIs) were calculated as effect sizes.

The extent and impact of between-study heterogeneity was assessed by inspecting the forest plots and by calculating the tau^2^ (absolute heterogeneity) and the *I*^2^ statistic (relative heterogeneity), respectively. *I*^2^ defines the proportion of total variability explained by heterogeneity (not chance) in the results. An *I*^2^ statistic over 75% was arbitrarily considered to represent considerable heterogeneity, while also considering the heterogeneity’s direction (localization on the forest plot) and uncertainty intervals around heterogeneity estimates [20]. Ninety-five percent predictive intervals, which are crucial for the correct interpretation of random-effects meta-analyses [21], were calculated for meta-analyses of ≥ 3 trials to incorporate existing heterogeneity and provide a range of possible effects for a future clinical setting.

### Additional analyses and risk of bias across studies

Possible sources of heterogeneity were a priori planned to be sought through subgroup analyses and random-effects meta-regression in meta-analyses of at least five trials but could not be ultimately performed (Appendix 1). Likewise, reporting biases were planned, but they were not assessed due to the limited number of meta-analyzed trials.

The overall quality of meta-evidence (i.e., the strength of clinical recommendations) was rated using the Grades of Recommendations, Assessment, Development, and Evaluation (GRADE) approach [22] following recent guidance on synthesizing non-randomized studies [23], and summary of findings tables were constructed using the improved format proposed by Carrasco-Labra et al. [24]. The minimal clinically important, large, and very large effects were defined as half, one, and two standard deviations of the response of the control (adolescents) group [25]. The produced forest plots were augmented with contours denoting the magnitude of the observed effects to assess heterogeneity, clinical relevance, and imprecision [26].

Robustness of the results was planned to be checked a priori with sensitivity analyses based on (a) inclusion/exclusion of non-randomized studies, (b) inclusion/exclusion of trials with methodological shortcomings, and (c) improvement of the GRADE classification. In the end, only one sensitivity analysis excluding non-randomized studies with methodological shortcomings could be conducted (Appendix 1).

All the analyses were run in Stata version 14.0 (StataCorp LP, College Station, TX, USA) by one author (SNP) and the dataset is openly available [27]. All *P* values were two-sided with α = 5%, except for the test of between-studies or between-subgroups heterogeneity, where α value was set at 10% [28].

## Results

### Study selection

**Fig. 1:**
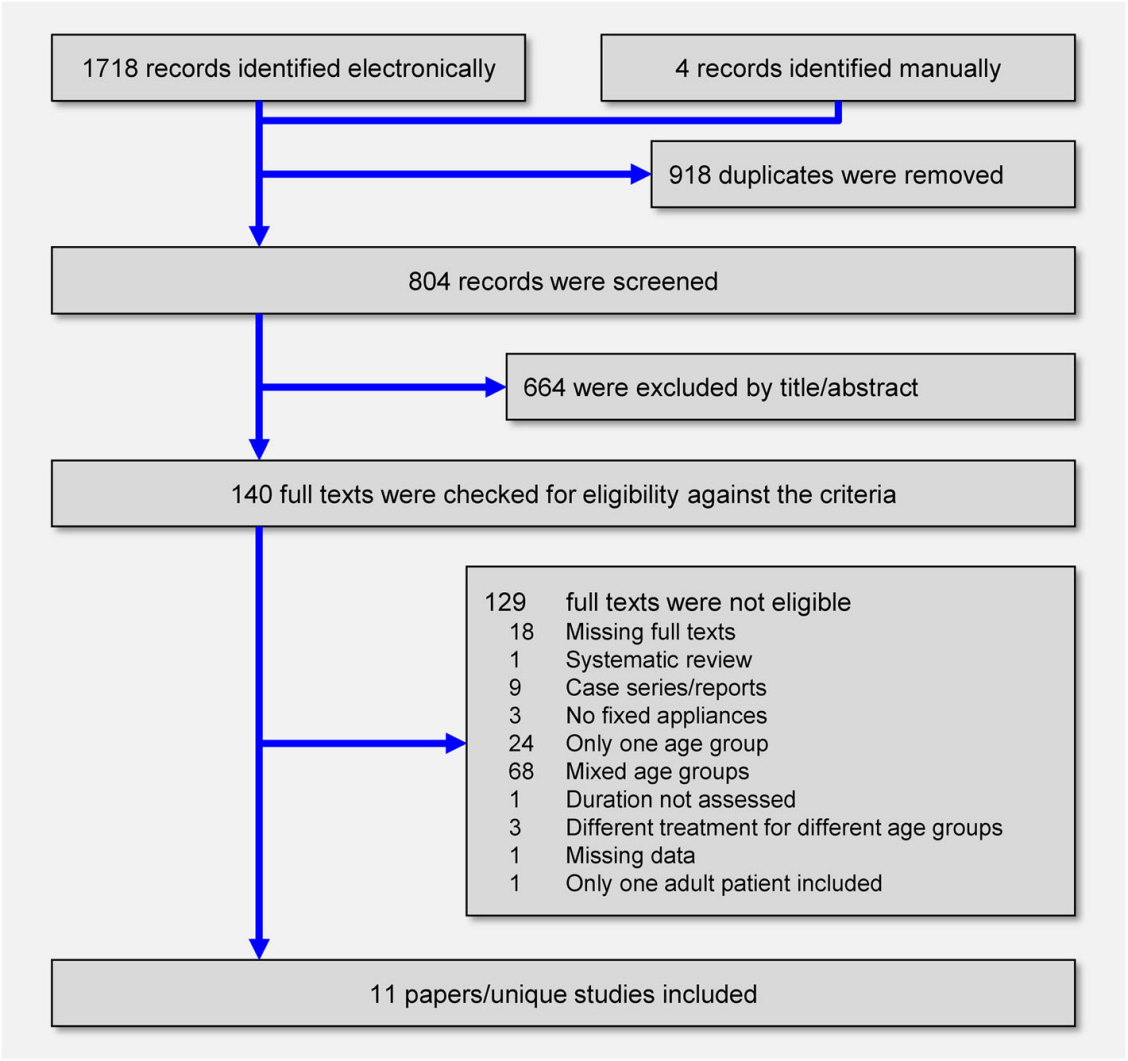
PRISMA flow diagram for the identification and selection of eligible studies

The electronic literature search yielded 1718 results, while 4 studies were manually identified from the reference list of the identified papers (Fig. 1). After duplicate removal and screening of titles/abstracts against the pre-defined eligibility criteria (Appendix 4), the full texts of 140 papers were checked. One study [29] was excluded post hoc, since it included only one adult patient, which made statistical comparisons between adolescents and adults patients difficult. Eventually, 11 papers pertaining to 11 unique studies (1 prospective and 10 retrospective non-randomized studies), which were published as journal papers, were finally included (Table 1) [1, 31–40].

**Table 1.** Characteristics of included studies.

Study	Design; setting; country^**a**^	Patients (M/F); mean age^**b**^	Malocclusion	Treatment	Severity	Appliance	Ex
Bhattarai 2011 [30]	rNRS; Uni; NP	AD: 134 (NR); 13.6 ADU: 46 (NR); 23.1	No impactions, 2-phase Tx, or non-compliant patients; all permanent teeth except M3	Full Tx	NR	Roth FA 0.018” (loops, elastics; HG)	NR
Dyer 1991 [1]	rNRS; Pract; US	AD: 30 (0/30); 12.5 ADU: 26 (0/26); 27.6	Cl. II/1; all permanent teeth except M3	Full Tx	≥ ½ Cl. II MR	SE FA 0.022” (elastics, HG, sliding jigs)	4xPM
Furquim 2018 [31]	rNRS; Pract; BR	AD: 23 (10/13); 11.8 ADU: 16 (7/9); 22.4	Cl. II	Full Tx	NR	FA and MPA	NR
Harris 1990 [32]	rNRS; Pract; US	AD^c^: 29 (0/29); 12.5 ADU^c^: 30 (0/30); 27.9	Cl. II/1; all permanent teeth except M3	Full Tx	≥ ½ Cl. II MR	FA (SDFET)	4xPM
Iancu 2018 [33]	rNRS; Uni; IT	AD: 19 (8/11); 13.8 ADU: 3 (2/1); 23.7	PDC	PDC alignment	NR	OSE; FA 0.022 (TPA, CAN)	NR
Jiang 2017 [34]	pNRS; Uni; US	AD: 10 (6/4); 14.7 ADU: 8 (1/7); 25.1	Need for Mx canine retraction	Canine retraction	NR	FA 0.019” (T-loops 124cN; TPA)	2x Mx PM
Loke 2012 [35]	rNRS; Pract; MY	AD^c:^ 716 (NR); NR ADU^c^: 156 (NR); NR	No syndromes, CLP, or only RFA; Cl. I (28%), II (57%), III (15%); impactions (7%)	Full Tx	NR	FA ± Mx removable appliance, functional appliance, or surgery	Ex (74%)
Nienkemper 2014 [36]	rNRS; Uni; DE	AD: 37 (17/20); 12.9 ADU: 14 (4/10); 30.9	≥ ¼ bilateral Cl. II MR or anterior Mx crowding	Full Tx	≥ ¼ Cl. II MR	MI-distalizer	NR
Robb 1998 [37]	rNRS; Pract; US	AD^d^: 40 (15/25); 12.9 ADU^d^: 32 (12/20); 31.3	Cl. I (94%) or II (6%)	Full Tx	NR	FA	4xPM
Sachdeva 2012 [38]	rNRS; Pract; US	AD: 1861 (NR); NR ADU: 979 (NR); NR	Cl. I, II, or III	Full Tx	Mean PAR = 25.5	FA	NR
Shim 2011 [39]	rNRS; Pract; KR	AD^c^: 70 (35/35); NR ADU^c^: 70 (35/35); NR	No root resorptions, root-fillings, or trauma	Full Tx	NR	SE FA	Ex PM1 (55%)

*rNRS* retrospective non-randomized study, *pNRS* prospective non-randomized study, *Uni* university clinic, *Pract* private practice, *AD* adolescent, *ADU* adult; NR, not reported, *Tx* treatment, *M3* 3rd molars, *Cl.* angle’s class, *CLP* cleft lip and palate, *RFA* removable functional appliance, *MR* molar relationship, *Mx* maxillary, *PDC* palatally displaced canine, *PAR* Peer Assessment Rating, *FA* fixed appliance, *HG* headgear, *SE* standard edgewise, *MPA* mandibular protraction appliance, *SDFET* sequential directional force edgewise technique, *MI* miniscrew implant, *OSE* open surgical exposure, *TPA* transpalatal arch, *CAN* cantilever, *PM* premolar, *Ex* extraction of permanent teeth

^a^Given with the country’s ISO 3166 alpha-2 code

^b^With 18 years of age taken as cut-off for adults, except if otherwise noted

^c^20 years taken as cut-off for adults

^d^21 years taken as cut-off for adults.

### Study characteristics

The primary studies were conducted in university clinics (*n* = 4; 36%) or private practices (*n* = 7; 64%) and originated from seven different countries (Brazil, Germany, Italy, Malaysia, Nepal, South Korea, and the USA) (Table 1). A total of 2969 adolescents and 1380 adult patients were included with a median total sample of 59 patients per included study (range 18 to 2840 patients per study). Out of the 8 studies reporting on patient sex, 152 (33%) of the 457 patients in total were male, while the mean age for adolescents and adults was 13.1 and 26.7 years, respectively, in the 8 studies providing data.

Nine of the included studies assessed comprehensive orthodontic treatment with fixed appliances, while one of them also included patients, whose treatment plan involved removable or functional appliances and orthognathic surgery [36]. One of the studies on comprehensive fixed appliance treatment compared conventional fixed appliances with the Suresmile appliances [39]. This comparison falls outside this review’s scope and data for conventional appliances was therefore only included. The other two studies solely assessed either orthodontic alignment of palatally displaced canines [34] or retraction of maxillary canines into premolar extraction spaces [35]. These are reported separately.

As far as complexity of the treated cases is concerned, this was defined in the inclusion criteria of the primary studies in only three studies [1, 33, 37] and consisted of a minimum Class II molar relationship of a quarter (one study) or half cusp (two studies). As far as tooth extractions are concerned, 4 studies (36%) did not report on extractions, 4 studies (36%) performed extractions on all patients, and 2 studies (18%) included both extraction and non-extraction cases.

### Risk of bias within studies

The included non-randomized trials presented several issues that increased their risk for bias (Table 2). Even though all included non-randomized studies were prone to confounding and did not use any kind of matching, three studies (27%) were judged to be in moderate risk of bias for confounding, as they included patients with similar baseline severity and who were treated with similar appliances. The remaining 8 studies (73%) either did not report on these confounders or had obvious baseline discrepancies. Five studies (45%) were in moderate or serious risk of bias for the selection of participants as they included either not representative cases of the average patient or recruited patients being treated at different periods. All studies did not blind the outcome assessor and were judged to be in moderate risk of bias for outcome measurement, even though it is unclear how this might affect the reported results. Finally, all studies were judged to be in low risk of bias for (a) classification of interventions (exposure), (b) deviations from intended interventions, (c) missing data, and (d) selection of the reported result.

**Table 2:** Assessment of included non-randomized studies with the ROBINS-I tool

Domain	Reference	Bhattarai 2011 [30]	Dyer 1991 [1]	Furquim 2018 [31]	Harris 1990 [32]	Iancu 2018 [33]	Jiang 2017 [34]	Loke 2012 [35]	Nienkemper 2014 [36]	Robb 1998 [37]	Sachdeva 2012 [38]	Shim 2011 [39]
**1. Confounding**	**1.1**	**Y**	**Y**	**Y**	**Y**	**Y**	**Y**	**Y**	**Y**	**Y**	**Y**	**Y**
	**1.2**	**N**	**N**	**N**	**N**	**N**	**N**	**N**	**N**	**N**	**N**	**N**
	**1.3**	**N**	**N**	**N**	**N**	**N**	**N**	**N**	**N**	**N**	**N**	**N**
	**1.4**	**NI**	**PY**	**PN**	**PY**	**N**	**NI**	**NI**	**PY**	**NI**	**NI**	**NI**
	**1.5**	**N**	**Y**	**PY**	**Y**	**Y**	**N**	**N**	**Y**	**N**	**N**	**N**
	**1.6**	**PN**	**PN**	**PN**	**PN**	**PN**	**PN**	**PN**	**PN**	**PN**	**PN**	**PN**
	**1.7**	**N**	**NA**	**N**	**NA**	**N**	**N**	**N**	**NA**	**N**	**N**	**N**
	**1.8**	**NA**	**NA**	**NA**	**NA**	**NA**	**NA**	**NA**	**NA**	**NA**	**NA**	**NA**
	**Judgement**	**Serious**	**Moderate**	**Serious**	**Moderate**	**Serious**	**Serious**	**Serious**	**Moderate**	**Serious**	**Serious**	**Serious**
**2. Selection of participants into the study**	**2.1**	**Y**	**PN**	**N**	**PN**	**N**	**N**	**Y**	**PN**	**NI**	**NI**	**PN**
	**2.2**	**Y**	**N**	**N**	**N**	**N**	**N**	**Y**	**N**	**NA**	**NA**	**N**
	**2.3**	**Y**	**N**	**N**	**N**	**N**	**N**	**Y**	**N**	**NA**	**NA**	**N**
	**2.4**	**PY**	**PY**	**PY**	**PY**	**Y**	**Y**	**NI**	**PY**	**PN**	**PN**	**PN**
	**2.5**	**NA**	**NA**	**NA**	**NA**	**NA**	**NA**	**NA**	**NA**	**N**	**N**	**N**
	**Judgement**	**Serious**	**Low**	**Low**	**Low**	**Low**	**Low**	**Serious**	**Low**	**Moderate**	**Moderate**	**Moderate**
**3. Classification of interventions**	**3.1**	**Y**	**Y**	**Y**	**Y**	**Y**	**Y**	**Y**	**PY**	**Y**	**Y**	**Y**
	**3.2**	**Y**	**Y**	**Y**	**Y**	**Y**	**Y**	**Y**	**Y**	**Y**	**Y**	**Y**
	**3.3**	**N**	**N**	**N**	**N**	**N**	**N**	**N**	**N**	**N**	**N**	**N**
	**Judgement**	**Low**	**Low**	**Low**	**Low**	**Low**	**Low**	**Low**	**Low**	**Low**	**Low**	**Low**
**4. Deviations from intended interventions**	**4.1**	**NI**	**NI**	**NI**	**NI**	**NI**	**NI**	**NI**	**NI**	**NI**	**NI**	**NI**
	**4.2**	**NA**	**NA**	**NA**	**NA**	**NA**	**NA**	**NA**	**NA**	**NA**	**NA**	**NA**
	**4.3**	**NI**	**Y**	**NI**	**Y**	**Y**	**Y**	**NI**	**Y**	**NI**	**NI**	**NI**
	**4.4**	**NI**	**NI**	**PY**	**PY**	**PY**	**PY**	**NI**	**Y**	**Y**	**NI**	**NI**
	**4.5**	**PY**	**PY**	**PY**	**PY**	**PY**	**PY**	**PY**	**PY**	**PY**	**PY**	**PY**
	**4.6**	**NA**	**NA**	**NA**	**NA**	**NA**	**NA**	**NA**	**NA**	**NA**	**NA**	**NA**
	**Judgement**	**Low**	**Low**	**Low**	**Low**	**Low**	**Low**	**Low**	**Low**	**Low**	**Low**	**Low**
**5. Missing data**	**5.1**	**NI**	**NI**	**NI**	**NI**	**NI**	**NI**	**NI**	**NI**	**NI**	**NI**	**NI**
	**5.2**	**PN**	**PN**	**PN**	**PN**	**PN**	**PN**	**PN**	**PN**	**PN**	**PN**	**PN**
	**5.3**	**PN**	**PN**	**PN**	**PN**	**PN**	**PN**	**PN**	**PN**	**PN**	**PN**	**PN**
	**5.4**	**NA**	**NA**	**NA**	**NA**	**NA**	**NA**	**NA**	**NA**	**NA**	**NA**	**NA**
	**5.5**	**NΑ**	**NΑ**	**NΑ**	**NΑ**	**NΑ**	**NΑ**	**NΑ**	**NΑ**	**NΑ**	**NΑ**	**NΑ**
	**Judgement**	**NI**	**NI**	**NI**	**NI**	**NI**	**NI**	**NI**	**NI**	**NI**	**NI**	**NI**
**6. Measurement of outcomes**	**6.1**	**PY**	**PY**	**PY**	**PY**	**PY**	**PY**	**PY**	**PY**	**PY**	**PY**	**PY**
	**6.2**	**PY**	**PY**	**PY**	**PY**	**PY**	**PY**	**PY**	**PY**	**PY**	**PY**	**PY**
	**6.3**	**PY**	**PY**	**PY**	**PY**	**PY**	**PY**	**PY**	**PY**	**PY**	**PY**	**PY**
	**6.4**	**PN**	**PN**	**PN**	**PN**	**PN**	**PN**	**PN**	**PN**	**PN**	**PN**	**PN**
	**Judgement**	**Moderate**	**Moderate**	**Moderate**	**Moderate**	**Moderate**	**Moderate**	**Moderate**	**Moderate**	**Moderate**	**Moderate**	**Moderate**
**7. Selection of the reported result**	**7.1**	**PN**	**PN**	**PN**	**PN**	**PN**	**PN**	**PN**	**PN**	**PN**	**PN**	**PN**
	**7.2**	**PN**	**PN**	**PN**	**PN**	**PN**	**PN**	**PN**	**PN**	**PN**	**PN**	**PN**
	**7.3**	**PN**	**PN**	**PN**	**PN**	**PN**	**PN**	**PN**	**PN**	**PN**	**PN**	**PN**
	**Judgement**	**Low**	**Low**	**Low**	**Low**	**Low**	**Low**	**Low**	**Low**	**Low**	**Low**	**Low**
**Overall**	**Judgement**	**Serious**	**Moderate**	**Serious**	**Moderate**	**Serious**	**Serious**	**Serious**	**Moderate**	**Serious**	**Serious**	**Serious**

*N* no, *NA* not applicable, *NI* no information, *PN* probably not, *PY* probably yes, *Y* yes

### Data synthesis

**Fig. 2:**
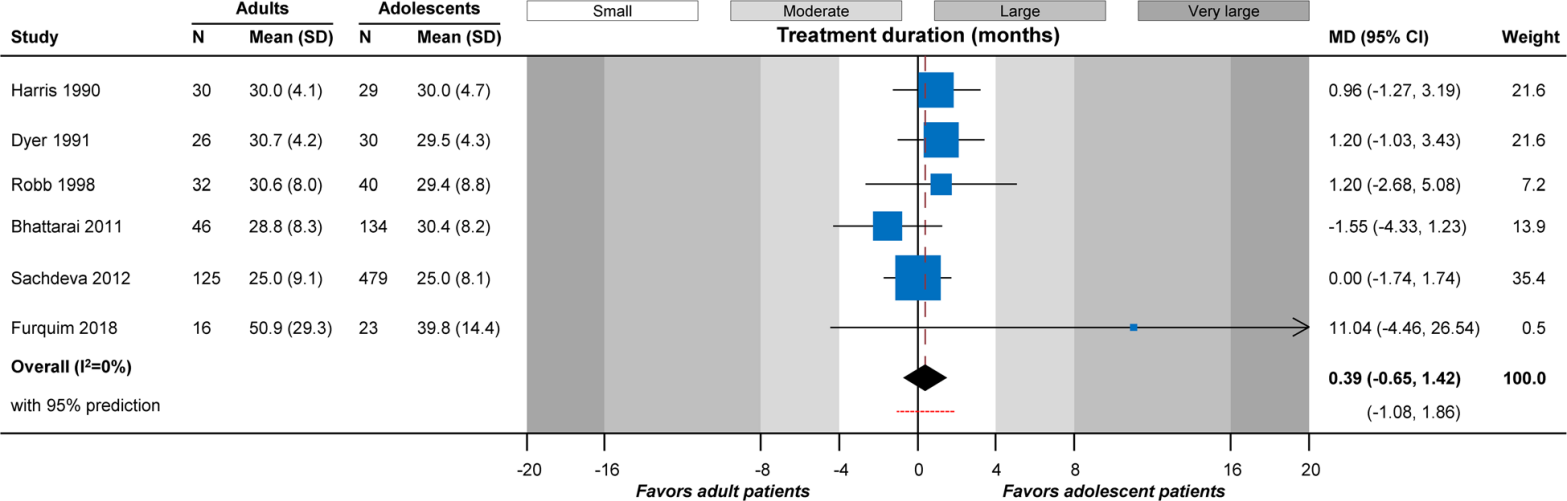
Contour-enhanced forest plot for the duration of comprehensive treatment among adolescents and adult patients. N, number of patients; SD, standard deviation; MD, mean difference; CI, confidence interval

A total of 7 studies with 1150 patients comparing the duration of comprehensive treatment with fixed appliances among adolescents and adults were eligible for meta-analysis, the results of which indicated no statistically significant difference (7 studies; MD = − 0.8 months; 95% CI = − 4.2 to 2.6 months; *P* = 0.65; Table 3). However, extreme heterogeneity was observed among studies both in absolute (tau^2^ = 17.05) and relative terms (*I*^2^ = 92%), which might render data synthesis problematic. Therefore, the most extreme study of Shim et al. [40] was excluded in order to achieve a homogeneous data synthesis. The results of this updated meta-analysis still indicated no difference in treatment duration between adolescents and adults (6 studies; MD = 0.4 months; 95% CI = − 0.7 to 1.4 months; *P* = 0.47; Fig. 2) with minimal absolute and relative homogeneity (tau^2^ = 0 and *I*^2^ = 0%).

**Table 3:** Details of performed analyses

Treatment	Analysis	Studies (patients)	MD (95% CI)	***P*** value	***I***^**2**^ (95% CI)	tau^**2**^ (95% CI)	95% prediction
Complete treatment (conventional appliances)^a^	Original	7 (1150)	− 0.79 (− 4.18, 2.61)	0.65	92% (77%, 99%)	17.05 (5.19, 134.34)	− 12.30, 10.72
	Sensitivity; omitting Shim 2011	6 (1010)	0.39 (− 0.65, 1.42)	0.47	0% (0%, 98%)	0 (0, 73.07)	− 1.08, 1.86
Alignment of displaced canine	Original	1 (30)	3.79 (1.42, 6.16)	0.002	–	–	–
Distalization of 1st molar	Original	1 (51)	0.06 (− 1.66, 1.78)	0.95	–	–	–
Retraction of canine	Original	1 (18)	2.02 (− 0.49, 4.53)	0.12	–	–	–

*MD* mean difference, *CI* confidence interval

^a^Original analysis gives a very heterogeneous picture, which is probably incompatible with synthesis of the studies; the sensitivity analysis probably gives a more stable image and should be preferred

### Results of individual studies

In single studies, no statistically significant differences between adolescents and adults in treatment times either for upper first molar distalization with skeletal anchorage (1 study; MD = 0.1 month; 95% CI = − 1.7 to 1.8 months; *P* = 0.95) or retraction of canine into the extraction space of the first premolar (1 study; MD = 2.0 months; 95% CI = − 0.5 to 4.5 months; *P* = 0.12) were found. However, alignment of palatally displaced canines lasted an average of 3.8 months longer in adults compared with adolescents (1 study; 95% CI = 1.4 to 6.2 months; *P* = 0.002).

### Additional analyses, risk of bias across studies, and quality of evidence

Several subgroup analyses, meta-regressions, and assessments for reporting biases were originally planned in the review’s protocol, but they could not be eventually performed due to limited data and inadequate reporting (Appendix 1). One subgroup analysis could be performed according to the inclusion of tooth extractions, where three studies consistently extracted teeth in all patients (MD = 1.1 month; 95% CI = − 0.4 to 2.6 months; *P* = 0.14) and three studies did not report at all on extractions (MD = − 0.3 months; 95% CI = − 1.8 to 1.1 months; *P* = 0.66), with no significant between subgroup difference (*P* = 0.25).

The quality of evidence (Table 4) for the main meta-analysis of comprehensive treatment duration of six studies was very low, due to the inclusion of non-randomized studies with considerable risk of bias. The quality of evidence for the two meta-analyses on the duration of first upper molar distalization (1 study) and canine retraction (1 study) was low to very low, due to the inclusion of non-randomized studies and imprecision from limited analyzed samples. Finally, the quality of evidence of the meta-analysis that reported significantly longer alignment duration for palatally displaced canines in adult patients (1 study) was similarly very low due to bias and imprecision. Overall, the low to very low GRADE for all analyzed comparisons means that further research in terms of well-designed studies is very likely to have an important impact, which is likely to change our current estimates of effect.

**Table 4:** Summary of findings table according to the GRADE approach

	Anticipated absolute effects (95% CI)			
Outcome Studies (patients)	Adolescents	Difference in adults	Quality of the evidence (GRADE)^**b**^	What happens with adults
Full Tx duration 1010 patients (6 studies)	30.1 months ^a^	0.4 months more (0.7 months less to 1.4 months more)	⊕◯◯◯ very low^c^ due to bias	Little to no difference in overall treatment duration
Duration of PDC alignment 30 patients (1 study)	3.0 months	3.8 months more (1.4 to 6.2 months more)	⊕◯◯◯ very low^c,d^ due to bias, imprecision	Might be associated with longer alignment of PDCs
Duration of 1^st^ molar distalization 51 patients (1 study)	7.4 months	0.1 month more (1.7 months less to 1.8 months more)	⊕⊕◯◯ very low^d,e^ due to bias, imprecision	Little to no difference in duration of 1st molar distalization
Duration of canine retraction 18 patients (1 study)	4.0 months	2.0 months more (0.5 month less to 4.5 months more)	⊕◯◯◯ very low^c,d^ due to bias, imprecision	Little to no difference in duration of canine retraction

Intervention: comprehensive orthodontic treatment with fixed appliances/population: adolescents or adult patients with any kind of malocclusion/setting: university clinics, private practices (Brazil, Germany, Italy, Malaysia, Nepal, South Korea, USA)

*CI* confidence interval, *GRADE* Grading of Recommendations Assessment, Development and Evaluation, *Tx* treatment, *PDC* palatally displaced canine, *mo* month

^a^Response in the control group is based on random-effects meta-analysis of the adolescent groups of included studies

^b^Starts from “high”

^c^Downgraded by two to three levels for bias due to the inclusion of non-randomized studies with serious risk of bias

^d^Downgraded by one level for imprecision due to the inclusion of an inadequate sample

^e^Downgraded by one level for bias due to the inclusion of non-randomized studies with moderate risk of bias

### Sensitivity analysis

No sensitivity analysis could be performed by omitting non-randomized studies, as only non-randomized studies were included. Sensitivity analysis according to the risk of bias by including only 2 of the 6 studies, which were in moderate risk of bias, still gave similar results (2 studies; MD = 1.1 months; 95% CI = − 0.5 to 2.7 months; *P* = 0.18) to the original analysis.

## Discussion

### Results in context

To our knowledge, this is the first study to systematically assess existing evidence on the duration of orthodontic treatment with fixed appliances in adult and adolescent patients. Eleven studies were finally included according to the review’s eligibility criteria and 7 (one prospective and six retrospective) with a total of 1150 patients were meta-analyzed.

As far as the review’s main scope is concerned, meta-analysis of the seven included studies found no statistically significant difference in treatment duration between adults and adolescents (*P* = 0.65; Table 3). Lower responsiveness to orthodontic forces as well as lower rates of tooth movement have been reported for adults compared to younger patients only with respect to the initial phase of tooth movement [40]. Moreover, it is important to note that all studies included here reported differences of very small magnitude (i.e., they were in the white portion of Fig. 2) and have probably limited clinical relevance. This might, therefore, indicate that any delays in tooth movement due to biological differences [6] might be counterbalanced by a potentially better compliance of adult patients in keeping their appointments and adhering to the orthodontist’s instructions, which have a direct effect on treatment duration [37].

On the other hand, the duration for the alignment of palatally displaced canines was significantly longer for adult patients compared to adolescents in one included study (MD = 3.8 months; Table 3). This is not in agreement with Stewart et al. [41], who found a positive association between young age and severity of displacement as well as longer treatment time. Yet, treatment of displaced canines presents considerable differences according to patients’ characteristics, tooth localization, and treatment methods [42]. Besides, older patients also have significantly higher odds for ankylosis of the impacted canines once orthodontic traction has been applied to them [43].

Finally, no statistically significant difference was found in the duration of either distalization of the maxillary first molars with skeletal anchorage or the retraction of upper canines after premolar extraction. As far as distalization of the maxillary first molars is concerned, some studies have reported that it is more difficult in older patients when the second molars have already erupted [44], which could indicate that longer distalization times might be expected in adults. However, the protocol in the included study [36] utilized forces in the upper third of the usual spectrum [45] in order to account for friction losses and forces were adapted constantly, which also provided adequate distalization for adults. Finally, as far as canine retraction is concerned, although the single identified study [34] found no statistically significant difference in duration of retraction, considerably higher root resorption for adult patients was reported, which is corroborated by previous data [46] and might indicate underlying differences in the physiology of tooth movement and the tissue response [47].

### Strengths and limitations

This systematic review has several strengths, comprising an a priori registered protocol [48], a comprehensive literature search, the use of modern analytic methods [19], the application of the GRADE approach to assess the strength of provided recommendations [22], and the transparent availability of all data [27].

However, some limitations do also exist at the same time. Firstly, methodological issues existed for all included studies that might influence results and that is especially the case for included retrospective non-randomized studies [49]. Inclusion of non-randomized studies in meta-analyses is not considered prohibitory, provided that robust bias appraisal has been performed and recent guidance has been provided on how to appropriately incorporate such designs [23]. Secondly, most meta-analyses were predominantly based on small trials, which might affect the precision of the estimates [50]. Thirdly, the small number of trials included in meta-analyses and their incomplete reporting of results and potential confounders, such as case severity, different cut-off ages for adulthood, treatment appliances/techniques, and treatment outcome quality, precluded from conducting many subgroup analyses and meta-regressions, which could enable identification of treatments that might take longer in adult patients. Finally, a potential overlap of age groups might exist at some point in-treatment due to the length of the comprehensive treatment, although mean ages for included adolescents and adults were 13.1 and 26.7 years, respectively.

## Conclusions

Based on available evidence from mostly retrospective non-randomized studies assessing adult and adolescent patients, no statistically and clinically significant difference in the duration of comprehensive orthodontic treatment with fixed appliances was found. However, existing studies on the topic have serious methodological limitations and future studies with transparent reporting of treatment procedures, objective outcome assessment, and adequate handling of confounders are needed to robustly tackle this topic.

## Supplementary information

**Additional file 1.** Appendix 1-4

## Data Availability

All data generated or analyzed during this study are included in this published article or its supplements, while its dataset is openly provided through Zenodo (10.5281/zenodo.3631110).

## Abbreviations

CI: Confidence interval
GRADE: Grading of Recommendations Assessment, Development, and Evaluation
MD: Mean difference
PICOS: Participants-Interventions-Comparisons-Outcome-Study design
