# Supplementary material for: Duration of orthodontic treatment with fixed appliances in adolescents and adults: a systematic review with meta-analysis
Source: Prog Orthod. 2020 Oct 5;21:37. doi: 10.1186/s40510-020-00334-4 (PMC7533275; doi:10.1186/s40510-020-00334-4)
Supplement: Supplementary file 1 — Additional file 1. Appendix 1-4 [file 40510_2020_334_MOESM1_ESM.pdf]

Supplementary material

Appendix 1. Additional review details and deviations from the protocol.

**Deviations from the protocol**
Several factors were planned to be assessed through subgroup analyses/meta-regressions in meta-analyses of at least 5 studies, but they could not be ultimately conducted due to limited material/reporting: (i) subsets according to the patient sample characteristics (age, sex, malocclusion type, malocclusion severity, skeletal configuration); (ii) subsets according to any additional appliances/adjuncts used in treatment; (iii) the operator's experience with each system. Additionally, sensitivity analyses regarding i) inclusion/exclusion of non-randomized studies and (ii) improvement of the GRADE classification were not performed.

Appendix 2. Eligibility criteria for the inclusion of primary studies.

	**Inclusion criteria**	**Exclusion criteria**
Patients	Human patients of any age, sex, ethnicity, or malocclusion	Systemic diseases, syndromes, disorders
Intervention	Any orthodontic treatment with fixed appliances	No fixed appliances
Control	Adolescent patients (up to 18 years, unless otherwise noted)	-
Outcome	Treatment duration in months	-
Studies	Randomized and prospective or retrospective non-randomized clinical trials	Animal studies, case reports/series, non-clinical studies, and cross-sectional studies

Appendix 3. Literature search (as of September 28th, 2019) for each database with the corresponding hits.

Databank	Search	Limits	Hits
MEDLINE (through PubMed)	orthodon* AND (therap* OR treatm*) AND (adult* OR overage* OR old* OR elder* OR senior* OR mature* OR “non-growing”) AND (adolesc* OR underage* OR young* OR child* OR teen* OR juvenile* OR immature* OR youth* OR growing) AND (duration* OR "treatment time")	Humans	479
Embase	Same as MEDLINE	Human	191
Web of Knowledge	Same as MEDLINE	DENTISTRY ORAL SURGERY MEDICINE	562
Scopus	TITLE-ABS-KEY (orthodon* AND (therap* OR treatm*) AND (adult* OR overage* OR old* OR elder* OR senior* OR mature* OR "non-growing") AND (adolesc* OR underage* OR young* OR child* OR teen* OR juvenile* OR immature* OR youth* OR growing) AND (duration* OR "treatment time")) AND (LIMIT-TO (SUBJAREA , "DENT"))	Dentistry	375
CDSR	Same as MEDLINE		4
DARE	Same as MEDLINE		0
CENTRAL	Same as MEDLINE		71
Virtual Health Library	Same as MEDLINE		36
**SUM (with overlaps)**			1718
**SUM (without overlaps)**			800

CDSR, Cochrane Database of Systematic Reviews; DARE, Cochrane Database of Abstracts of Reviews of Effects;CENTRAL, Cochrane Central Register of Controlled Trials.

Appendix 4. List of studies identified from the literature search and their inclusion/exclusion status with reasons.

Nr	Paper	Status
1	{ACTRN12615000341527} Efficacy of oral probiotics in improving oral hygiene for orthodontic patients. 2015.	Excluded by title
2	{CTRI/2018/12/016793} New Approach For Faster Tooth Movement. 2018.	Excluded by title
3	{DRKS00012463} Duration of tooth brushing with a manual and an electric toothbrush in children and adults with fixed appliances. 2017.	Excluded by title
4	{ISRCTN05771195} An investigation of two methods of orthodontic space closure: nickel titanium versus stainless steel springs. 2013.	Excluded by title
5	{JPRN-UMIN000022182} Comprehensive clinical evaluation of indirect bonding method in orthodontic treatment. 2016.	Excluded by title
6	{NCT02659813} Orthodontic Archwire Effectiveness Trial. 2016.	Excluded by title
7	{NCT03251807} Effect of Low-Intensity Pulsed Ultrasound on Functional Treatment of Class II Malocclusion. 2017.	Excluded by title
8	{NCT03547531} Comparison of Modified Circular and Natural Tooth Brushing Methods in Effectiveness of Dental Plaque Removal. 2018.	Excluded by title
9	{NCT0364119} Orthodontic Approaches to Correct Deep Bite in Mixed Dentition Patients. 2018.	Excluded by title
10	Abellán R, Gómez C, Iglesias-Linares A, Palma JC. Impact of photodynamic therapy versus ultrasonic scaler on gingival health during treatment with orthodontic fixed appliances. Lasers in Surgery and Medicine. 2019;51(3):256-67.	Excluded by title
11	Aboul-Ela SMBED, El-Beialy AR, El-Sayed KMF, Selim EMN, El-Mangoury NH, Mostafa YA. Miniscrew implant-supported maxillary canine retraction with and without corticotomy-facilitated orthodontics. Am J Orthod Dentofacial Orthop 2011;139(2):252-9.	Excluded by title
12	Abtahi M, Jahanbin A, Yaghoubi M, Esmaily H, Zare H. Are more nickel ions accumulated in the hair of fixed orthodontic patients? Indian J Dent Res. 2013;24(3):298-301.	Excluded by title
13	Afzal A, Qamruddin I. Relation between centric slide and Angle's classification. J Coll Physicians Surg Pak. 2005;15(8):481-4.	Excluded by title
14	Agaoglu G, Arun T, Izgi B, Yarat A. Nickel and chromium levels in the saliva and serum of patients with fixed orthodontic appliances. Angle Orthod. 2001;71(5):375-9.	Excluded by title
15	Ahmed I, Saif ul H, Nazir R. Carious lesions in patients undergoing orthodontic treatment. Journal of the Pakistan Medical Association. 2011;61(12):1176-9.	Excluded by title
16	Aileni KR, Rachala MR. Early treatment of class III malocclusion with Petit facemask therapy. Int J Orthod Milwaukee. 2011;22(4):41-5.	Excluded by title
17	Akbulut N, Altan A, Akbulut S, Atakan C. Evaluation of the 3 mm Thickness Splint Therapy on Temporomandibular Joint Disorders (TMDs). Pain Res Manag. 2018;2018:3756587.	Excluded by title
18	Akhare PJ, Daga AM, Pharande S. Rapid canine retraction and orthodontic treatment with dentoalveolar distraction osteogenesis. Journal of Clinical and Diagnostic Research. 2012;5(7):1473-7.	Excluded by title
19	Al-Ainawi KI, Al-Mdalal Y, Hajeer MY. The Effect of Using a Modified Dentoalveolar Distractor on Canine Angulation following Rapid Canine Retraction: A Split-mouth Design Randomized Controlled Trial. J Contemp Dent Pract. 2016;17(1):49-57.	Excluded by title
20	Alam MK, Imran A, Enezei HH, Shahid F, Nowrin SA, Rahman SA. Inter disciplinary management of maxillary lateral incisors agenesis with implant prostheses: A case report. International Journal of Pharma and Bio Sciences. 2015;6(3):B1185-B93.	Excluded by title
21	Albaker BK, Wong RWK. Diagnosis and management of root resorption by erupting canines using cone-beam computed tomography and fixed palatal appliance: A case report. Journal of Medical Case Reports. 2010;4.	Excluded by title
22	Alberconi TF, Siqueira GLC, Sathler R, Kelly KA, Garib DG. Assessment of Orthodontic Burden of Care in Patients With Unilateral Complete Cleft Lip and Palate. Cleft Palate Craniofac J. 2018;55(1):74-8.	Excluded by title
23	Alessandri-Bonetti G, D'Anto V, Stipa C, Rongo R, Incerti-Parenti S, Michelotti A. Dentoskeletal effects of oral appliance wear in obstructive sleep apnoea and snoring patients. Eur J Orthod. 2017;39(5):482-8.	Excluded by title
24	Alfaro-Moctezuma P, Osorno-Escareno MD, Nuno-Licona A, Leiva-Cartes F, Angeles-Medina F. Effects of orthodontic treatment on the inhibitory masseteric reflex. Revista De Investigacion Clinica-Clinical and Translational Investigation. 2003;55(3):289-96.	Excluded by title
25	Alfaro-Moctezuma P, Osorno-Escareno Mdel C, Nuno-Licona A, Leiva-Cartes F, Angeles-Medina F. (Effects of orthodontic treatment on the masseter muscle inhibitory reflex). Rev Invest Clin. 2003;55(3):289-96.	Excluded by title
26	Alfawal AMH, Hajeer MY, Ajaj MA, Hamadah O, Brad B. Evaluation of piezocision and laser-assisted flapless corticotomy in the acceleration of canine retraction: a randomized controlled trial. Head Face Med. 2018;14(1):4.	Excluded by title
27	Alhaija ESA, Al-Saif EM, Taani DQ. Periodontal health knowledge and awareness among subjects with fixed orthodontic appliance. Dental Press J Orthod. 2018;23(5):40.e1-.e9.	Excluded by title
28	Ali Z, Shafique S, Sheikh AA, Hussain SS. Three years audit of maxillofacial trauma at Abbasi Shaheed Hospital, Karachi. Medical Forum Monthly. 2014;25(5):73-6.	Excluded by title
29	Allereau B, Sabouni W. (Perception of pain in orthodontic treatment with thermoformed aligners]. Orthod Fr. 2017;88(4):383-9.	Excluded by title

30	Al-Melh MA, Andersson L. The effect of a lidocaine/prilocaine topical anesthetic on pain and discomfort associated with orthodontic elastomeric separator placement. Prog Orthod. 2017;18(1):1.	Excluded by title
31	Alqerban A, Willems G, Bernaerts C, Vangastel J, Politis C, Jacobs R. Orthodontic treatment planning for impacted maxillary canines using conventional records versus 3D CBCT. Eur J Orthod. 2014;36(6):698-707.	Excluded by title
32	Al-Saleem AI, Al-Jobair AM. Possible association between acetazolamide administration during pregnancy and multiple congenital malformations. Drug Design, Development and Therapy. 2016;10:1471-6.	Excluded by title
33	Altug Z, Akcam OU. Treatment of a young adult with Class III malocclusion using a modified mini maxillary protractor: a case report. J Oral Sci. 2010;52(1):155-9.	Excluded by title
34	Alwas-Danowska HM. The effect of direct and alternating electrical currents on the vessel walls of the tooth pulp - TEM studies. Folia Morphologica. 2004;63(1):137-9.	Excluded by title
35	Amin M. Postpubertal cherubism with noonan syndrome. Journal of the College of Physicians and Surgeons Pakistan. 2014;24:S39-S40.	Excluded by title
36	Antoszewska J, Papadopoulos MA, Park HS, Ludwig B. Five-year experience with orthodontic miniscrew implants: a retrospective investigation of factors influencing success rates. Am J Orthod Dentofacial Orthop. 2009;136(2):158.e1-10; discussion -9.	Excluded by title
37	Anwar N, Fida M. Clinical applicability of variations in arch dimensions and arch forms among various vertical facial patterns. J Coll Physicians Surg Pak. 2011;21(11):685-90.	Excluded by title
38	Anwar N, Fida M. Evaluation of dentoalveolar compensation in skeletal class II malocclusion in a Pakistani University Hospital setting. J Coll Physicians Surg Pak. 2009;19(1):11-6.	Excluded by title
39	Anwar N, Fida M. Variability of arch forms in various vertical facial patterns. J Coll Physicians Surg Pak. 2010;20(9):565-70.	Excluded by title
40	Arad I, Jandu J, Bassett P, Fleming PS. Influence of single-jaw surgery vs bimaxillary surgery on the outcome and duration of combined orthodontic-surgical treatment. Angle Orthod. 2011;81(6):983-7.	Excluded by title
41	Araujo EA, Araujo CV, Tanaka OM. Apicotomy: Surgical management of maxillary dilacerated or ankylosed canines. Am J Orthod Dentofacial Orthop 2013;144(6):909-15.	Excluded by title
42	Ari-Demirkaya A, Masry MA, Erverdi N. Apical root resorption of maxillary first molars after intrusion with zygomatic skeletal anchorage. Angle Orthod. 2005;75(5):761-7.	Excluded by title
43	Aristizabal JF, Martinez-Smit R, Diaz C, Pereira Filho VA. Surgery-first approach with 3D customized passive self-ligating brackets and 3D surgical planning: Case report. Dental Press J Orthod. 2018;23(3):47-57.	Excluded by title
44	Arponen H, Vuorimies I, Haukka J, Valta H, Waltimo-Siren J, Makitie O. Cranial base pathology in pediatric osteogenesis imperfecta patients treated with bisphosphonates. J Neurosurg Pediatr. 2015;15(3):313-20.	Excluded by title
45	Ashraf I, Ashraf S, Mohammad N, Alam MK. Pharmacist-strengthen adherence to antiretroviral therapy and the contributing factors among HIV-infected paediatric patients in Nigeria. Bangladesh Journal of Medical Science. 2017;16(2):281-8.	Excluded by title
46	Asiry MA. Anterior open bite treated with myofunctional therapy and palatal crib. J Contemp Dent Pract. 2015;16(3):243-7.	Excluded by title
47	Askar J. European College of Orthodontics: Commission of affiliation and titularisation. Int Orthod. 2018;16(4):776-89.	Excluded by title
48	Austin DF, Chaiyongsirisern A, Yang Y, Wong RWK, Rabie ABM. A protocol for improved stability with Herbst Appliance Treatment for adults. Progress in Orthodontics. 2010;11(2):151-6.	Excluded by title
49	Awasthi E, Sanjay K, Bhonghade ML, Shrivastav S. Alveolar bone housing- A modified wilkodontics approach- A case report. Journal of Clinical and Diagnostic Research. 2016;10(8):ZD12-ZD5.	Excluded by title
50	Baccetti T, Franchi L, McNamara Jr JA. Growth in the Untreated Class III Subject. Seminars in Orthodontics. 2007;13(3):130-42.	Excluded by title
51	Baek SH, Kim BM, Kyung SH, Lim JK, Kim YH. Success rate and risk factors associated with mini-implants reinstalled in the maxilla. Angle Orthod. 2008;78(5):895-901.	Excluded by title
52	Baek SH, Kim KW, Choi JY. New treatment modality for maxillary hypoplasia in cleft patients. Protraction facemask with miniplate anchorage. Angle Orthod. 2010;80(4):783-91.	Excluded by title
53	Baek SH, Park YH, Chung JH, Kim S, Choi JY. Orthodontic and orthopedic treatment for a growing patient with Tessier number 0 cleft. Korean Journal of Orthodontics. 2018;48(2):113-24.	Excluded by title
54	Bahammam MA. Effectiveness of bovine-derived xenograft versus bioactive glass with periodontally accelerated osteogenic orthodontics in adults: a randomized, controlled clinical trial. BMC Oral Health. 2016;16(1):126.	Excluded by title
55	Baik UB, Kim MR, Yoon KH, Kook YA, Park JH. Orthodontic uprighting of a horizontally impacted third molar and protraction of mandibular second and third molars into the missing first molar space for a patient with posterior crossbites. Am J Orthod Dentofacial Orthop. 2017;151(3):572-82.	Excluded by title
56	Baik UB, Kook YA, Bayomec M, Park JU, Park JH. Vertical eruption patterns of impacted mandibular third molars after the mesialization of second molars using miniscrews. Angle Orthodontist. 2016;86(4):565-70.	Excluded by title
57	Bain C, Sayed AA, Kaklamanos EG, Kazi HA. Toothbrushing-Should We Advise Mechanical or Power Brushes? Results of an International Delphi Conference. The journal of contemporary dental practice. 2018;19(10):1169-73.	Excluded by title
58	Baker RS, Fields HW, Jr., Beck FM, Firestone AR, Rosenstiel SF. Objective assessment of the contribution of dental esthetics and facial attractiveness in men via eye tracking. Am J Orthod Dentofacial Orthop. 2018;153(4):523-33.	Excluded by title
59	Barbagallo LJ, Jones AS, Petocz P, Darendeliler MA. Physical properties of root cementum: Part 10.	Excluded by title

	Comparison of the effects of invisible removable thermoplastic appliances with light and heavy orthodontic forces on premolar cementum. A microcomputed-tomography study. Am J Orthod Dentofacial Orthop. 2008;133(2):218-27.	
60	Barlow ST, Moore MB, Sherriff M, Ireland AJ, Sandy JR. Palatally impacted canines and the modified index of orthodontic treatment need. Eur J Orthod 2009;31(4):362-6.	Excluded by title
61	Bartella AK, Ghassemi M, Holzle F, Ghassemi A. Reconstruction of facial soft tissue: comparison between conventional procedures and the facelift technique. Br J Oral Maxillofac Surg. 2016;54(9):1006-11.	Excluded by title
62	Bauer W, Wehrbein H, Schulte-Luenzum H, Diedrich P. Germ Transplantation or Space Closure a Comparative Study of the Loss of First Molars. Fortschritte der Kieferorthopaedie. 1991;52(2):84-92.	Excluded by title
63	Bauer W, Wehrbein H, Schulte-Lunzum H, Diedrich P. [Tooth germ transplantation or gap closure--a comparative study in the loss of the first molar]. Fortschr Kieferorthop. 1991;52(2):84-92.	Excluded by title
64	Becker A, Abramovitz I, Chaushu S. Failure of treatment of impacted canines associated with invasive cervical root resorption. Angle Orthodontist. 2013;83(5):870-6.	Excluded by title
65	Becker A, Chaushu S. Success rate and duration of orthodontic treatment for adult patients with palatally impacted maxillary canines. Am J Orthod Dentofacial Orthop. 2003;124(5):509-14.	Excluded by title
66	Bell WH, Dann Iii JJ. Correction of dentofacial deformities by surgery in the anterior part of the jaws. A study of stability and soft-tissue changes. American Journal of Orthodontics. 1973;64(2):162-87.	Excluded by title
67	Bell WH, Yamaguchi Y, Poor MR. Treatment of temporomandibular joint dysfunction by intraoral vertical ramus osteotomy. Int J Adult Orthodon Orthognath Surg. 1990;5(1):9-27.	Excluded by title
68	Bencini AC, Bencini LE. Técnica de ortodoncia osteogénica periodontalmente acelerada: principios biológicos y etapa quirúrgica. Rev Soc Odontol La Plata. 2018;28(55):7-18.	Excluded by title
69	Bengi AO, Karacay S, Akin E, Olmez H, Okcu KM, Mermut S. Use of zygomatic anchors during rapid canine distalization: a preliminary case report. Angle Orthod. 2006;76(1):137-47.	Excluded by title
70	Benic GZ, Farella M, Morgan XC, Viswam J, Heng NC, Cannon RD, et al. Oral probiotics reduce halitosis in patients wearing orthodontic braces: A randomized, triple-blind, placebo-controlled trial. Journal of Breath Research. 2019;13(3).	Excluded by title
71	Bergstrom K, Halling A, Huggare J. Orthodontic treatment demand--differences between urban and rural areas. Community Dent Health. 1998;15(4):272-6.	Excluded by title
72	Berneburg M, Zeyher C, Merkle T, Moller M, Schaupp E, Goz G. Orthodontic findings in 4- to 6-year-old kindergarten children from southwest Germany. J Orofac Orthop. 2010;71(3):174-86.	Excluded by title
73	Bessette R, Bishop B, Mohl N. Duration of Masseteric Silent Period in Patients with Tmj Syndrome. Journal of Applied Physiology. 1971;30(6):864-&.	Excluded by title
74	Bhandari R, Thakur S, Singhal P, Chauhan D, Jayam C, Jain T. Fixed hexa-helix: An amended quad helix for a compliance driven pediatric patient- a innovative case approach. International Journal of Pharmaceutical Sciences and Research. 2018;9(8):3535-7.	Excluded by title
75	Bianchi J, Pinto ADS, Ignacio J, Obelenis Ryan DP, Goncalves JR. Effect of temporomandibular joint articular disc repositioning on anterior open-bite malocclusion: An orthodontic-surgical approach. Am J Orthod Dentofacial Orthop. 2017;152(6):848-58.	Excluded by title
76	Bindayel NA. Simple removable appliances to correct anterior and posterior crossbite in mixed dentition: Case report. Saudi Dental Journal. 2012;24(2):105-13.	Excluded by title
77	Birlutiu V, Birlutiu RM, Costache VS. Viridans streptococcal infective endocarditis associated with fixed orthodontic appliance managed surgically by mitral valve plasty. Medicine (United States). 2018;97(27).	Excluded by title
78	Boboc G, Tolea M, Oltean D, Gaucan C, Tanasescu D, Dragoi E, et al. [Contribution of immediate orthopedic-orthodontic treatment in maxillo-palatine-labial clefts]. Rev Chir Oncol Radiol O R L Oftalmol Stomatol Ser Stomatol. 1988;35(3):227-40.	Excluded by title
79	Bock NC, Ruf S. Dentoskeletal changes in adult Class II division 1 Herbst treatment-how much is left after the retention period? Eur J Orthod 2012;34(6):747-53.	Excluded by title
80	Bockow R, Korostoff J, Pinto A, Hutcheson M, Secreto SA, Bodner L, et al. Characterization and treatment of postsurgical dental implant pain employing intranasal ketorolac. Compend Contin Educ Dent. 2013;34(8):570-6.	Excluded by title
81	Bonetti GA, Parenti SI, Daprile G, Montevecchi M. Failure after closed traction of an unerupted maxillary permanent canine: Diagnosis and treatment planning. Am J Orthod Dentofacial Orthop 2011;140(1):121-5.	Excluded by title
82	Bonnet E. [Proper procedure for adhesive reconstructions with an emphasis on maintaining aesthetics: treating people from 7 to 77 years]. Orthod Fr. 2012;83(2):143-52.	Excluded by title
83	Borzabadi-Farahani A. Effect of low-level laser irradiation on proliferation of human dental mesenchymal stem cells; a systemic review. Journal of Photochemistry and Photobiology B: Biology. 2016;162:577-82.	Excluded by title
84	Bourzgui F, Sebbar M, Nadour A, Hamza M. Prevalence of temporomandibular dysfunction in orthodontic treatment. Int Orthod. 2010;8(4):386-98.	Excluded by title
85	Breuning KH, van Strijen PJ, Prahl-Andersen B, Tuinzing DB. Duration of orthodontic treatment and mandibular lengthening by means of distraction or bilateral sagittal split osteotomy in patients with Angle Class II malocclusions. Am J Orthod Dentofacial Orthop. 2005;127(1):25-9.	Excluded by title
86	Brooks JK, Schwartz KG, Basile JR. Superficial mucocele of the ventral tongue: Presentation of a rare case and literature review. Journal of Oral and Maxillofacial Surgery. 2016;74(6):1175-9.	Excluded by title
87	Brugnami F, Caiazzo A, Dibart S. Lingual orthodontics: accelerated realignment of the "social six" with piezocision. Compend Contin Educ Dent. 2013;34(8):608-10.	Excluded by title
88	Burki S, Sheraz S. Skeletal relapse following orthognathic surgery in angle's class-III cases. Journal of	Excluded by title

	the College of Physicians and Surgeons Pakistan. 2002;12(2):92-6.	
89	Burns B, Grieg V, Bissell V, Savarrio L. A review of implant provision for hypodontia patients within a Scottish referral centre. Br Dent J. 2017;223(2):96-9.	Excluded by title
90	Buttke TM, Proffit WR. Referring adult patients for orthodontic treatment. J Am Dent Assoc. 1999;130(1):73-9.	Excluded by title
91	Caccianiga G, Crestale C, Cozzani M, Piras A, Mutinelli S, Lo Giudice A, et al. Low level laser therapy and invisible removal aligners. Journal of Biological Regulators and Homeostatic Agents. 2016;30(2):107-13.	Excluded by title
92	Cakmak F, Turk T, Sumer M. Advancement of the premaxilla with distraction osteogenesis. Eur J Orthod. 2014;36(3):321-30.	Excluded by title
93	Camacho M, Chang ET, Song SA, Abdullatif J, Zaghi S, Pirelli P, et al. Rapid Maxillary Expansion for Pediatric Obstructive Sleep Apnea: A Systematic Review and Meta-Analysis. Laryngoscope. 2017;127(7):1712-9.	Excluded by title
94	Capistrano A, Cordeiro A, Siqueira DF, Capelozza Filho L, Cardoso Mde A, Almeida-Pedrin RR. From conventional to self-ligating bracket systems: is it possible to aggregate the experience with the former to the use of the latter? Dental Press J Orthod. 2014;19(3):139-57.	Excluded by title
95	Caprioglio A, Vanni A, Bolamperti L. Long-term periodontal response to orthodontic treatment of palatally impacted maxillary canines. Eur J Orthod. 2013;35(3):323-8.	Excluded by title
96	Cartwright G, Wright NS, Vasuvadev J, Akram S, Huppa C, Matthews NS, et al. Outcome of combined orthodontic-surgical treatment in a United Kingdom university dental institute. J Orthod. 2016;43(2):94-101.	Excluded by title
97	Cassetta M, Giansanti M, Di Mambro A, Calasso S, Barbato E. Minimally invasive corticotomy in orthodontics using a three-dimensional printed CAD/CAM surgical guide. Int J Oral Maxillofac Surg. 2016;45(9):1059-64.	Excluded by title
98	Cedströmer AL, Ahlqwist M, Andlin-Sobocki A, Berntson L, Hedenberg-Magnusson B, Dahlström L. Temporomandibular condylar alterations in juvenile idiopathic arthritis most common in longitudinally severe disease despite medical treatment. Pediatric Rheumatology. 2014;12(1).	Excluded by title
99	Chaushu S, Becker A, Chaushu G. Lingual orthodontic treatment and absolute anchorage to correct an impacted maxillary canine in an adult. Am J Orthod Dentofacial Orthop. 2008;134(6):811-9.	Excluded by title
100	Chaushu S, Casap N, Becker A, Tzur B, Chaushu G. [Orthodontic anchorage in the era of osseointegration]. Refuat Hapeh Vehashinayim (1993). 2006;24(3):32-45, 92.	Excluded by title
101	Chen YR, Yeow VK. Multiple-segment osteotomy in maxillofacial surgery. Plast Reconstr Surg. 1999;104(2):381-8.	Excluded by title
102	Chiu GSC, Chang CHN, Roberts WE. Bimaxillary protrusion with an atrophic alveolar defect: Orthodontics, autogenous chin-block graft, soft tissue augmentation, and an implant. Am J Orthod Dentofacial Orthop 2015;147(1):97-113.	Excluded by title
103	Cho I-S, Shin H-K, Back S-H. Preliminary study of Korean orthodontic residents' current concepts and knowledge of cleft lip and palate management. Korean Journal of Orthodontics. 2012;42(3):100-9.	Excluded by title
104	Choi SH, Cha JY, Lee KJ, Yu HS, Hwang CJ. Changes in psychological health, subjective food intake ability and oral health-related quality of life during orthodontic treatment. J Oral Rehabil. 2017;44(11):860-9.	Excluded by title
105	Choi YJ, Chung CJ, Kim KH. Periodontal consequences of mandibular incisor proclination during presurgical orthodontic treatment in Class III malocclusion patients. Angle Orthod. 2015;85(3):427-33.	Excluded by title
106	Choo H, Heo HA, Yoon HJ, Chung KR, Kim SH. Treatment outcome analysis of speedy surgical orthodontics for adults with maxillary protrusion. Am J Orthod Dentofacial Orthop. 2011;140(6):e251-62.	Excluded by title
107	Chu CH, Choy BH, Lo EC. Occlusion and orthodontic treatment demand among Chinese young adults in Hong Kong. Oral Health Prev Dent. 2009;7(1):83-91.	Excluded by title
108	Cioffi I, Michelotti A, Perrotta S, Chiodini P, Ohrbach R. Effect of somatosensory amplification and trait anxiety on experimentally induced orthodontic pain. Eur J Oral Sci. 2016;124(2):127-34.	Excluded by title
109	Cohen SC, Chase C. Human pulpal response to bleaching procedures on vital teeth. Journal of Endodontics. 1979;5(5):134-8.	Excluded by title
110	Condo R, Costacurta M, Perugia C, Docimo R. Atypical deglutition: diagnosis and interceptive treatment. A clinical study. Eur J Paediatr Dent. 2012;13(3):209-14.	Excluded by title
111	Conley RS, Boyd SB, Legan HL, Jernigan CC, Starling C, Potts C. Treatment of a patient with multiple impacted teeth. Angle Orthodontist. 2007;77(4):735-41.	Excluded by title
112	Cornelis MA, Scheffler NR, Mahy P, Siciliano S, De Clerck HJ, Tulloch JFC. Modified Miniplates for Temporary Skeletal Anchorage in Orthodontics: Placement and Removal Surgeries. Journal of Oral and Maxillofacial Surgery. 2008;66(7):1439-45.	Excluded by title
113	Costa JG, Galindo TM, Mattos CT, Cury-Saramago AdA. Retention period after treatment of posterior crossbite with maxillary expansion: a systematic review. Dental press j orthod (Impr). 2017;22(2):35-44.	Excluded by title
114	Cota R, Lucamba A, Henderson K. Third ectopic molar in orbit floor with associated dentigerous cyst. International Journal of Oral and Maxillofacial Surgery. 2019;48:222-3.	Excluded by title
115	Crescini A, Nieri M, Buti J, Baccetti T, Prato GPP. Pre-treatment radiographic features for the periodontal prognosis of treated impacted canines. Journal of Clinical Periodontology. 2007;34(7):581-7.	Excluded by title
116	Cros P, Freidel M, Borie J, Henry B, Bouvier P, Dumas P. [15 years of treatment of temporomandibular joint algo-dysfunctional syndromes]. Rev Stomatol Chir Maxillofac. 1989;90(6):409-14.	Excluded by title

117	Cruz DR, Kohara EK, Ribeiro MS, Wetter NU. Effects of low-intensity laser therapy on the orthodontic movement velocity of human teeth: A preliminary study. Lasers in Surgery and Medicine. 2004;35(2):117-20.	Excluded by title
118	Cutrera A, Allareddy V, Azami N, Nanda R, Uribe F. Is Short Root Anomaly (SRA) a risk factor for increased external apical root resorption in orthodontic patients? A retrospective case control study using cone beam computerized tomography. Orthod Craniofac Res. 2019;22(1):32-7.	Excluded by title
119	Dai F, Yu J, Chen G, Xu T, Jiang R. Changes in buccal facial depth of female patients after extraction and nonextraction orthodontic treatments: A preliminary study. Korean Journal of Orthodontics. 2018;48(3):172-81.	Excluded by title
120	Dardengo CdS. Comparação tridimensional dos efeitos dentários de duas mecânicas para fechamento de espaço: estudo preliminar. 2013.	Excluded by title
121	de Freitas MR, Beltrao RT, Janson G, Henriques JF, Chiqueto K. Evaluation of root resorption after open bite treatment with and without extractions. Am J Orthod Dentofacial Orthop. 2007;132(2):143.e15-22.	Excluded by title
122	de Lima DV, de Freitas KM, de Freitas MR, Janson G, Henriques JF, Pinzan A. Stability of molar relationship after non-extraction Class II malocclusion treatment. Dental Press J Orthod. 2013;18(2):42-54.	Excluded by title
123	de Menezes LM, de Oliveira RB, Weissheimer A, Avelar RL. Midfacial Protraction With Skeletal Anchorage After Pterygomaxillary Separation. J Craniofac Surg. 2016;27(6):1561-4.	Excluded by title
124	de Menezes VA, Cavalcanti LL, de Albuquerque TC, Garcia AFG, Leal RB. Mouth breathing within a multidisciplinary approach: Perception of orthodontists in the city of Recife, Brazil. Dental Press Journal of Orthodontics. 2012;16(6):84-92.	Excluded by title
125	Deguchi T, Imai M, Sugawara Y, Ando R, Kushima K, Takano-Yamamoto T. Clinical evaluation of a low-friction attachment device during canine retraction. Angle Orthodontist. 2007;77(6):968-72.	Excluded by title
126	Dermaut LR, De Munck A. Apical root resorption of upper incisors caused by intrusive tooth movement: a radiographic study. Am J Orthod Dentofacial Orthop. 1986;90(4):321-6.	Excluded by title
127	Dersot JM. Periodontal surgery of the maxillary impacted canine for orthodontic purposes: Proposal for a surgical decision tree. Int Orthod. 2017;15(2):221-37.	Excluded by title
128	Deshpande A, Deshpande N. Flexible Wire Composite Splinting for Root Fracture of Immature Permanent Incisors: A Case Report. Pediatric Dentistry. 2011;33(1):63-6.	Excluded by title
129	Dewinter G, Quirynen M, Heidbuchel K, Verdonck A, Willems G, Carels C. Dental abnormalities, bone graft quality, and periodontal conditions in patients with unilateral cleft lip and palate at different phases of orthodontic treatment. Cleft Palate Craniofac J. 2003;40(4):343-50.	Excluded by title
130	Dhole PM, Maheshwari DO. Two-phase orthodontic treatment in a unilateral cleft lip and palate patient with 1-year follow-up results. Apos Trends in Orthodontics. 2017;7(2):101-7.	Excluded by title
131	Diallo B, Ba AA, Dia-Tine S, Diagne F. [Surgical and orthodontic treatment of inclusions and dental retentions: report on the upper incisives and canines block]. Dakar Med. 2003;48(2):95-8.	Excluded by title
132	Diaz PM, Garcia RG, Gias LN, Aguirre-Jaime A, Perez JS, de la Plata MM, et al. Time used for orthodontic surgical treatment of dentofacial deformities in white patients. J Oral Maxillofac Surg. 2010;68(1):88-92.	Excluded by title
133	Dibart S, Surmenian J, Sebaoun JD, Montesani L. Rapid treatment of Class II malocclusion with piezocision: two case reports. Int J Periodontics Restorative Dent. 2010;30(5):487-93.	Excluded by title
134	Dindaroglu F, Dogan S, Yalcin A, Turkan N, Yuvruk E. How are faces with increased and decreased lower facial height perceived visually? International Journal of Computerized Dentistry. 2017;20(4):393-407.	Excluded by title
135	Dixit S, Singh A, Gs M, S Desai R, Jaju P. Apert's Syndrome: Report of a New Case and its Management. International journal of clinical pediatric dentistry. 2008;1(1):48-53.	Excluded by title
136	D'Onofrio L. Oral dysfunction as a cause of malocclusion. Orthodontics & Craniofacial Research. 2019;22:43-8.	Excluded by title
137	Dos Santos AA, Pithon MM, Carlo FG, Carlo HL, de Lima BA, Dos Passos TA, et al. Effect of time and pH on physical-chemical properties of orthodontic brackets and wires. Angle Orthod. 2015;85(2):298-304.	Excluded by title
138	Dowling PA, Espeland L, Krogstad O, Stenvik A, Kelly A. Duration of orthodontic treatment involving orthognathic surgery. Int J Adult Orthodon Orthognath Surg. 1999;14(2):146-52.	Excluded by title
139	Droschl H, Bantleon HP, Permann I. [Orthodontic treatment of adults]. Z Stomatol. 1989;86(1):13-22.	Excluded by title
140	Dugoni SA, Lee JS. Mixed dentition case report. Am J Orthod Dentofacial Orthop 1995;107(3):239-44.	Excluded by title
141	Durgekar SG, Kumar PS, Kolur N. Rapid canine retraction with periodontal distraction in Class II division 1 malocclusion: a case report. Int J Orthod Milwaukee. 2012;23(4):21-7.	Excluded by title
142	Echchadi ME, Benchikh B, Bellamine M, Kim SH. Corticotomy-assisted rapid maxillary expansion: A novel approach with a 3-year follow-up. Am J Orthod Dentofacial Orthop. 2015;148(1):138-53.	Excluded by title
143	Einy S, Horwitz J, Aizenbud D. Wilckodontics--an alternative adult orthodontic treatment method: rational and application. Alpha Omegan. 2011;104(3-4):102-11.	Excluded by title
144	El-Angbawi A, McIntyre GT, Fleming PS, Bearn DR. Non-surgical adjunctive interventions for accelerating tooth movement in patients undergoing fixed orthodontic treatment. Cochrane Database Syst Rev. 2015(11):Cd010887.	Excluded by title
145	Elkhadem A, Sheba M. Unclear if non-surgical adjuncts accelerate orthodontic treatment. Evid Based Dent. 2017;18(1):26-7.	Excluded by title
146	Enerback H, Moller M, Nylen C, Odman Bresin C, Ostman Ros I, Westerlund A. Effects of orthodontic treatment and different fluoride regimens on numbers of cariogenic bacteria and caries risk: a randomized controlled trial. Eur J Orthod. 2019;41(1):59-66.	Excluded by title

147	Enkling N, Marwinski G, Johren P. Dental anxiety in a representative sample of residents of a large German city. Clin Oral Investig. 2006;10(1):84-91.	Excluded by title
148	Ersahan S, Sabuncuoglu FA. Effect of age on pulpal blood flow in human teeth during orthodontic movement. J Oral Sci. 2018;60(3):446-52.	Excluded by title
149	Erverdi N, Keles A, Nanda R. The use of skeletal anchorage in open bite treatment: a cephalometric evaluation. Angle Orthod. 2004;74(3):381-90.	Excluded by title
150	Fah R, Schatzle M. Complications and adverse patient reactions associated with the surgical insertion and removal of palatal implants: a retrospective study. Clin Oral Implants Res. 2014;25(6):653-8.	Excluded by title
151	Falcini F, Melchiorre D, Cappelli S, Carnesecchi G, Biondi K, Bosco M, et al. Temporomandibular joints (TMJ) involvement in juvenile idiopathic arthritis (JIA): Longitudinal evaluation after orthopaedic treatment. Annals of the Rheumatic Disease. 2013;71.	Excluded by title
152	Falcini F, Melchiorre D, Carnesecchi G, Bertini F, Biondi K, Bosco M, et al. Orthopaedic treatment of temporomandibular joint (TMJ) damage in adolescents with juvenile idiopathic arthritis (JIA): Longitudinal evaluation. Arthritis and Rheumatism. 2012;64:S855.	Excluded by title
153	Farronato G, Lucchese A, Gherlone E, Bertossi D, Nocini PF, Rovati M, et al. Effect of thermosetting gel with doxycycline hyclate 3% on postoperative discomfort after third molar surgery: A prospective study. European Journal of Inflammation. 2013;11(2):553-8.	Excluded by title
154	Felicita AS. Orthodontic extrusion of Ellis Class VIII fracture of maxillary lateral incisor – The sling shot method. Saudi Dental Journal. 2018;30(3):265-9.	Excluded by title
155	Fernanda F, Daniela M, Lorenzo C, Serena C, Valentina D, Katia B, et al. Temporomandibular joint involvement (TMJ), a silent disease with severe alterations in young adulthood patients affected by juvenile idiophatic arthritis (JIA). Pediatric Rheumatology. 2011;9.	Excluded by title
156	Fernandes LPdS, Carvalho MSPd, Peixoto RCCP. Prevalência da gengivite e hábitos de higiene bucal de pacientes atendidos no curso de Especialização em Ortodontia da Universidade de Itaúna-MG. Ortho Sci, Orthod sci pract. 2011;4(16):757-64.	Excluded by title
157	Ferro R, Besostri A, Olivieri A, Quinzi V, Scibetta D. Prevalence of cross-bite in a sample of Italian preschoolers. European Journal of Paediatric Dentistry. 2016;17(4):307-9.	Excluded by title
158	Fish LC, Wolford LM, Epker BN. Surgical-orthodontic correction of vertical maxillary excess. Am J Orthod. 1978;73(3):241-57.	Excluded by title
159	Fontana M, Cozzani M, Mutinelli S, Spena R, Caprioglio A. Maxillary molar distalization therapy in adult patients: a multicentre study. Orthod Craniofac Res. 2015;18(4):221-31.	Excluded by title
160	Fornaini C, Merigo E, Vescovi P, Lagori G, Rocca JP. Use of laser in orthodontics: Applications and perspectives. Laser Therapy. 2013;22(2):115-24.	Excluded by title
161	Fornaini C, Rocca JP, Bertrand MF, Merigo E, Nammour S, Vescovi P. Nd:YAG and diode laser in the surgical management of soft tissues related to orthodontic treatment. Photomedicine and Laser Surgery. 2007;25(5):381-92.	Excluded by title
162	Foroughiasl P. Cautery versus laser excision of oral mucocele. Journal of Pediatric Surgery Case Reports. 2019;47.	Excluded by title
163	Fowler EB, Francis PO, Goho C. Use of acellular dermal matrix allograft for management of inadequate attached gingiva in a young patient. Mil Med. 2003;168(3):261-5.	Excluded by title
164	Freitas KMS, Guirro WJG, de Freitas DS, de Freitas MR, Janson G. Relapse of anterior crowding 3 and 33 years postretention. Am J Orthod Dentofacial Orthop. 2017;152(6):798-810.	Excluded by title
165	Freitas KMSd, Crepaldi A, Freitas MRd, Fonseca RC, Crepaldi MV. Estudo da recidiva da sobremordida relacionada com a curva de Spee, em pacientes Classe II, divisão 1, na fase pós-contenção. Rev dent press ortodon ortopedi facial. 2006;11(5):138-50.	Excluded by title
166	Freudenthaler JW, Haas R, Bantleon HP. Bicortical titanium screws for critical orthodontic anchorage in the mandible: a preliminary report on clinical applications. Clin Oral Implants Res. 2001;12(4):358-63.	Excluded by title
167	Fritz U, Diedrich P, Wiechmann D. Apical root resorption after lingual orthodontic therapy. J Orofac Orthop. 2003;64(6):434-42.	Excluded by title
168	Ganzer N, Feldmann I, Petren S, Bondemark L. A cost-effectiveness analysis of anchorage reinforcement with miniscrews and molar blocks in adolescents: a randomized controlled trial. Eur J Orthod. 2019;41(2):180-7.	Excluded by title
169	Garlock DT, Buschang PH, Araujo EA, Behrents RG, Kim KB. Evaluation of marginal alveolar bone in the anterior mandible with pretreatment and posttreatment computed tomography in nonextraction patients. Am J Orthod Dentofacial Orthop. 2016;149(2):192-201.	Excluded by title
170	Garnett MJ, Wassell RW, Jepson NJ, Nohl FS. Survival of resin-bonded bridgework provided for post-orthodontic hypodontia patients with missing maxillary lateral incisors. Br Dent J. 2006;201(8):527-34.	Excluded by title
171	Geisthoff UW, Heckmann K, D'Amelio R, Grünewald S, Knöbber D, Falkai P, et al. Health-related quality of life in hereditary hemorrhagic telangiectasia. Otolaryngology - Head and Neck Surgery. 2007;136(5):726.e1-.e10.	Excluded by title
172	Gerber B, Dhariwal D. Orthognathic Surgery in the Older patient - Are the complications higher? British Journal of Oral and Maxillofacial Surgery. 2017;55(10):e170.	Excluded by title
173	Ghassemi M, Jamilian A, Fritz U, Riediger D, Ghassemi A. Orthodontic treatment after autotransplantation. Angle Orthod. 2011;81(4):721-5.	Excluded by title
174	Gill DS, Naini FB, Jones A, Tredwin CJ. Part-time versus full-time retainer wear following fixed appliance therapy: a randomized prospective controlled trial. World J Orthod. 2007;8(3):300-6.	Excluded by title
175	Gillis I, Redlich M. The effect of different porcelain conditioning techniques on shear bond strength of stainless steel brackets. American journal of orthodontics and dentofacial orthopedics : official publication of the American Association of Orthodontists, its constituent societies, and the American	Excluded by title

	Board of Orthodontics. 1998;114(4):387-92.	
176	Giordano M, Turatti G, Parodi G, Luciani M, Lagana D. The maxillary protraction treatment: description of a laser Er:Yag-assisted surgical technique. Case report. Minerva Stomatol. 2009;58(6):307-15.	Excluded by title
177	Gizani S, Petsi G, Twetman S, Caroni C, Makou M, Papagianoulis L. Effect of the probiotic bacterium Lactobacillus reuteri on white spot lesion development in orthodontic patients. Eur J Orthod. 2016;38(1):85-9.	Excluded by title
178	Goerigk B, Diedrich P, Wehrbein H. [Intrusion of the anterior teeth with the segmented-arch technic of Burstone--a clinical study]. Fortschr Kieferorthop. 1992;53(1):16-25.	Excluded by title
179	Gong X, Yu M, Li WR, Gao XM. [Effect of oral appliance treatment on age-related changes of sleep respiratory function in patients with obstructive sleep apnea hypopnea syndrome]. Zhonghua Er Bi Yan Hou Tou Jing Wai Ke Za Zhi. 2019;54(6):410-5.	Excluded by title
180	Górska A, Przystupa W, Rutkowska-Sak L, Kwiatkowska M, Chlabicz S, Szarmach I. Temporomandibular joint dysfunction and disorders in the development of the mandible in patients with juvenile idiopathic arthritis - Preliminary study. Advances in Clinical and Experimental Medicine. 2014;23(5):797-804.	Excluded by title
181	Grande T, Stolze A, Goldbecher H, Kahl-Nieke B. The displaced maxillary canine--a retrospective study. J Orofac Orthop. 2006;67(6):441-9.	Excluded by title
182	Greenleaf S, Mink J. A retrospective study of the use of the Bluegrass appliance in the cessation of thumb habits. Pediatr Dent. 2003;25(6):587-90.	Excluded by title
183	Guedes FP, Araujo MCd, Medeiros RFB, Capelozza Filho L, Cardoso MdA. Metas terapêuticas individualizadas no tratamento ortodôntico compensatório das más oclusões do Padrão III: relato de caso. Ortho Sci, Orthod sci pract. 2012;5(17):60-71.	Excluded by title
184	Guerrero CA. Intraoral bone transport in clefting. Oral and Maxillofacial Surgery Clinics of North America. 2002;14(4):509-23.	Excluded by title
185	Gunduz E, Schneider-Del Savio TT, Kucher G, Schneider B, Bantleon HP. Acceptance rate of palatal implants: a questionnaire study. Am J Orthod Dentofacial Orthop. 2004;126(5):623-6.	Excluded by title
186	Gupta E, Sidhu MS, Grover S, Dabas A, Malik V, Dogra N. Measurement of perioral pressures at rest and its correlation with dental parameters in orthodontic patients with different occlusions. Journal of Clinical and Diagnostic Research. 2019;13(6):ZC13-ZC8.	Excluded by title
187	Gupta S, Kumar A, Sharma AK, Purohit J, Narula JS. "Sodium bicarbonate": an adjunct to painless palatal anesthesia. Oral Maxillofac Surg. 2018;22(4):451-5.	Excluded by title
188	Habib G, Un Nisa Z, Un Nisa Memon Q, Ul Hassan Q, Shams S. Mandibular fracture fixation with miniplate and MMF for up to two weeks. A prospective study. Medical Forum Monthly. 2014;25(10):3-5.	Excluded by title
189	Hagberg E, Flodin S, Granqvist S, Karsten A, Neovius E, Lohmander A. The Impact of Maxillary Advancement on Consonant Proficiency in Patients With Cleft Lip and Palate, Lay Listeners' Opinion, and Patients' Satisfaction With Speech. Cleft Palate-Craniofacial Journal. 2019;56(4):454-61.	Excluded by title
190	Hall B, Jamsa T, Soukka T, Peltomaki T. Duration of surgical-orthodontic treatment. Acta Odontol Scand. 2008;66(5):274-7.	Excluded by title
191	Hamersky PA, Weimer AD, Taintor JF. The effect of orthodontic force application on the pulpal tissue respiration rate in the human premolar. Am J Orthod. 1980;77(4):368-78.	Excluded by title
192	Han IS, Kim JH, Jin SB. [A case report of premolar autotransplantation]. Taehan Chikkwa Uisa Hyophoe Chi. 1991;29(5):389-94.	Excluded by title
193	Han J, Hwang S, Nguyen T, Proffit WR, Soma K, Choi YJ, et al. Periodontal and root changes after orthodontic treatment in middle-aged adults are similar to those in young adults. Am J Orthod Dentofacial Orthop. 2019;155(5):650-5.e2.	Excluded by title
194	Haque S, Alam MK, Arshad AI. An Overview of Indices Used to Measure Treatment Effectiveness in Patients with Cleft Lip and Palate. Malaysian Journal of Medical Sciences. 2015;22(1):4-11.	Excluded by title
195	Hariharan S, Narayanan V, Soh CL. Split-mouth comparison of Physics forceps and extraction forceps in orthodontic extraction of upper premolars. British Journal of Oral and Maxillofacial Surgery. 2014;52(10):e137-e40.	Excluded by title
196	Hazan-Molina H, Levin L, Einy S, Aizenbud D. Aggressive periodontitis diagnosed during or before orthodontic treatment. Acta Odontologica Scandinavica. 2013;71(5):1023-31.	Excluded by title
197	Hegde C, Hegde M. Mandibular incisor extractions in orthodontics: pitfalls and triumphs: a report of three cases. Int J Orthod Milwaukee. 2014;25(2):17-20.	Excluded by title
198	Heggie AAC, West RA, McNeill RW. Co-ordinated treatment of secondary cleft deformities. Australian Dental Journal. 1988;33(2):116-28.	Excluded by title
199	Heikinheimo K, Nystrom M, Heikinheimo T, Pirttiniemi P, Pirinen S. Dental arch width, overbite, and overjet in a Finnish population with normal occlusion between the ages of 7 and 32 years. Eur J Orthod. 2012;34(4):418-26.	Excluded by title
200	Hellal US, Fayed N, Elsharkawy R, Abdelrahmen M. Rapid Anterior Segmental Maxillary Retraction by Compression Osteogenesis. J Craniofac Surg. 2018;29(2):315-21.	Excluded by title
201	Hernandez-Alfaro F, Guijarro-Martinez R, Peiro-Guijarro MA. Surgery first in orthognathic surgery: what have we learned? A comprehensive workflow based on 45 consecutive cases. J Oral Maxillofac Surg. 2014;72(2):376-90.	Excluded by title
202	Hernandez-Alfaro F, Nieto MJ, Ruiz-Magaz V, Valls-Ontanon A, Mendez-Manjon I, Guijarro-Martinez R. Inferior subapical osteotomy for dentoalveolar decompensation of class III malocclusion in 'surgery-first' and 'surgery-early' orthognathic treatment. Int J Oral Maxillofac Surg. 2017;46(1):80-5.	Excluded by title
203	Hickman L, Firestone AR, Beck FM, Speer S. Eye fixations when viewing faces. J Am Dent Assoc.	Excluded by title

	2010;141(1):40-6.	
204	Hines FB, Jr. A radiographic evaluation of the response of previously avulsed teeth and partially avulsed teeth to orthodontic movement. Am J Orthod. 1979;75(1):1-19.	Excluded by title
205	Ho KH, Liao YF. Pre-treatment radiographic features predict root resorption of treated impacted maxillary central incisors. Orthod Craniofac Res. 2012;15(3):198-205.	Excluded by title
206	Hoffmann S, Papadopoulos N, Visel D, Visel T, Jost-Brinkmann PG, Prager TM. Influence of piezotomy and osteoperforation of the alveolar process on the rate of orthodontic tooth movement: a systematic review. J Orofac Orthop. 2017;78(4):301-11.	Excluded by title
207	Hohoff A, Joos U, Meyer U, Ehmer U, Stamm T. The spectrum of Apert syndrome: phenotype, particularities in orthodontic treatment, and characteristics of orthognathic surgery. Head Face Med. 2007;3:10.	Excluded by title
208	Homem RM, Freitas KMS, Valarelli FP, Cançado RH. Avaliação das alterações das dimensões dos arcos dentários pós-nivelamento com a utilização de aparelhos autoligáveis. Ortodontia. 2015;48(1):61-6.	Excluded by title
209	Hoogeveen EJ, Jansma J, Ren Y. Surgically facilitated orthodontic treatment: a systematic review. Am J Orthod Dentofacial Orthop. 2014;145(4 Suppl):S51-64.	Excluded by title
210	Huaman ET, Juvet LM, Nastri A, Denman WT, Kaban LB, Dodson TB. Changing patterns of hospital length of stay after orthognathic surgery. J Oral Maxillofac Surg. 2008;66(3):492-7.	Excluded by title
211	Huang J, Yao Y, Jiang J, Li C. Effects of motivational methods on oral hygiene of orthodontic patients A systematic review and meta-analysis. Medicine. 2018;97(47).	Excluded by title
212	Huang TT, Chang CJ, Chen KC, Lo JB, Chen MY, Huang JS. Outcome Analysis and Unexpected-Scenario Prediction in 2-Stage Orthodontic Lower Third Molar Extraction. J Oral Maxillofac Surg. 2018;76(3):503.e1-.e8.	Excluded by title
213	Hügle B, Spiegel L, Hotte J, Wiens S, Herlin T, Cron RQ, et al. Isolated arthritis of the temporomandibular joint as the initial manifestation of juvenile idiopathic arthritis. Journal of Rheumatology. 2017;44(11):1632-5.	Excluded by title
214	Ierardo G, Luzzi V, Nardacci G, Di Carlo G, Guaragna M, Covello F, et al. A modified rapid maxillary expander in a case of amelogenesis imperfect. Dental Cadmos. 2017;85(8):352-8.	Excluded by title
215	Ioannidou-Marathiotou I, Pistevou-Gompaki K, Eleftheriadis N, Papaloukas C. Long term chemoradiotherapy-related dental and skeletal complications in a young female with nasopharyngeal carcinoma. International Journal of General Medicine. 2010;3:187-96.	Excluded by title
216	Isaac A, Major M, Witmans M, Alrajhi Y, Flores-Mir C, Major P, et al. Correlations between acoustic rhinometry, subjective symptoms, and endoscopic findings in symptomatic children with nasal obstruction. JAMA Otolaryngology - Head and Neck Surgery. 2015;141(6):550-5.	Excluded by title
217	Isola G, Matarese G, Cordasco G, Perillo L, Ramaglia L. Mechanobiology of the tooth movement during the orthodontic treatment: a literature review. Minerva Stomatol. 2016;65(5):299-327.	Excluded by title
218	Ize-Iyamu IN, Saheeb BD, Edetanlen BE. Comparing the 810nm diode laser with conventional surgery in orthodontic soft tissue procedures. Ghana Med J. 2013;47(3):107-11.	Excluded by title
219	Jackson A, Lemke R, Hatch J, Salome N, Gakunga P, Cochran D. A comparison of stability between delayed versus immediately loaded orthodontic palatal implants. J Esthet Restor Dent. 2008;20(3):174-84.	Excluded by title
220	Jacobs JD, Bell WH. Combined surgical and orthodontic treatment of bimaxillary protrusion. Am J Orthod. 1983;83(4):321-33.	Excluded by title
221	Jain RK, Kumar SP, Manjula WS. Comparison of intrusion effects on maxillary incisors among mini implant anchorage, J-hook headgear and utility arch. Journal of Clinical and Diagnostic Research. 2014;8(7):21-4.	Excluded by title
222	Jambi S, Walsh T, Sandler J, Benson PE, Skeggs RM, O'Brien KD. Reinforcement of anchorage during orthodontic brace treatment with implants or other surgical methods. Cochrane Database Syst Rev. 2014(8):Cd005098.	Excluded by title
223	Janson G, Putrick LM, Henriques JF, de Freitas MR, Henriques RP. Maxillary third molar position in Class II malocclusions: the effect of treatment with and without maxillary premolar extractions. Eur J Orthod. 2006;28(6):573-9.	Excluded by title
224	Jeong JH, Choi SH, Kim KD, Hwang CJ, Lee SH, Yu HS. Long-Term Stability of Pre-Orthodontic Orthognathic Bimaxillary Surgery Using Intraoral Vertical Ramus Osteotomy Versus Conventional Surgery. Journal of Oral and Maxillofacial Surgery. 2018;76(8):1753-62.	Excluded by title
225	Jeong WS, Choi JW, Kim DY, Lee JY, Kwon SM. Can a surgery-first orthognathic approach reduce the total treatment time? Int J Oral Maxillofac Surg. 2017;46(4):473-82.	Excluded by title
226	Jiang C, Liu Y, Cheng Q, He W, Fang S, Lan T, et al. Chin remodeling in a patient with bimaxillary protrusion and open bite by using mini-implants for temporary anchorage. Am J Orthod Dentofacial Orthop. 2018;153(3):436-44.	Excluded by title
227	Jiang RP, Zhang D, Fu MK. [A factors study of root resorption after orthodontic treatment]. Zhonghua Kou Qiang Yi Xue Za Zhi. 2003;38(6):455-7.	Excluded by title
228	Joh B, Bayome M, Park JH, Park JU, Kim Y, Kook YA. Evaluation of minimal versus conventional presurgical orthodontics in skeletal class III patients treated with two-jaw surgery. J Oral Maxillofac Surg. 2013;71(10):1733-41.	Excluded by title
229	Johal A, Ashari AB, Alamiri N, Fleming PS, Qureshi U, Cox S, et al. Pain experience in adults undergoing treatment: A longitudinal evaluation. Angle Orthodontist. 2018;88(3):292-8.	Excluded by title
230	Johnson EK, Fields HW, Jr., Beck FM, Firestone AR, Rosenstiel SF. Role of facial attractiveness in patients with slight-to-borderline treatment need according to the Aesthetic Component of the Index of Orthodontic Treatment Need as judged by eye tracking. Am J Orthod Dentofacial Orthop.	Excluded by title

	2017;151(2):297-310.	
231	Josefsson E, Karlander EL. Traumatic injuries to permanent teeth among Swedish school children living in a rural area. Swed Dent J. 1994;18(3):87-94.	Excluded by title
232	Jung BA, Yildizhan F, Wehrbein H. Bone-to-implant contact of orthodontic implants in humans--a histomorphometric investigation. Eur J Orthod. 2008;30(6):552-7.	Excluded by title
233	Kalladka M. Dental sleep medicine. Sleep and Vigilance. 2017;1(2):137-8.	Excluded by title
234	Kang S-H, Kim M-K, Park S-Y, Lee J-Y, Park W, Lee S-H. Early Orthognathic Surgery With Three-Dimensional Image Simulation During Presurgical Orthodontics in Adults. Journal of Craniofacial Surgery. 2011;22(2):473-81.	Excluded by title
235	Kanwal S, Ul Hameed W. Frequency of dental caries in patients undergoing orthodontic treatment. Pakistan Journal of Medical and Health Sciences. 2014;8(1):219-20.	Excluded by title
236	Karkhanechi M, Chow D, Sipkin J, Sherman D, Boylan RJ, Norman RG, et al. Periodontal status of adult patients treated with fixed buccal appliances and removable aligners over one year of active orthodontic therapy. Angle Orthod. 2013;83(1):146-51.	Excluded by title
237	Karthik R, Hafila MIF, Saravanan C, Vivek N, Priyadarsini P, Ashwath B. Assessing Prevalence of Temporomandibular Disorders among University Students: A Questionnaire Study. Journal of International Society of Preventive and Community Dentistry. 2017;7:24-9.	Excluded by title
238	Kau CH, Kantarci A, Shaughnessy T, Vachiramon A, Santiwong P, de la Fuente A, et al. Photobiomodulation accelerates orthodontic alignment in the early phase of treatment. Prog Orthod. 2013;14:30.	Excluded by title
239	Kehoe JC. Splinting and replantation after traumatic avulsion. J Am Dent Assoc. 1986;112(2):224-30.	Excluded by title
240	Keles A. Unilateral distalization of a maxillary molar with sliding mechanics: a case report. J Orthod. 2002;29(2):97-100.	Excluded by title
241	Khan M, Fida M. Assessment of Psychosocial Impact of Dental Aesthetics. Jcpsp-Journal of the College of Physicians and Surgeons Pakistan. 2008;18(9):559-64.	Excluded by title
242	Khan RMS, Hassan KR, Rizwan M, Ashraf J. Prevalence and type of oral mucosal lesions in patients with fixed orthodontic appliances. Medical Forum Monthly. 2016;27(4):12-5.	Excluded by title
243	Kharkar VR, Kotrashetti SM, Kulkarni P. Comparative evaluation of dento-alveolar distraction and periodontal distraction assisted rapid retraction of the maxillary canine: a pilot study. Int J Oral Maxillofac Surg. 2010;39(11):1074-9.	Excluded by title
244	Kharkar VR, Kotrashetti SM. Transport dentoalveolar distraction osteogenesis-assisted rapid orthodontic canine retraction. Oral Surg Oral Med Oral Pathol Oral Radiol Endod. 2010;109(5):687-93.	Excluded by title
245	Kim HH, Ha HR, Ahn HW, Kim SJ. Anterior Decompensation Using Segmental Osteotomy for Patients With Mandibular Asymmetry. J Oral Maxillofac Surg. 2015;73(7):1392.e1-22.	Excluded by title
246	Kim JW, Lee NK, Yun PY, Moon SW, Kim YK. Postsurgical stability after mandibular setback surgery with minimal orthodontic preparation following upper premolar extraction. J Oral Maxillofac Surg. 2013;71(11):1968.e1-.e11.	Excluded by title
247	Kim JY, Jung HD, Kim SY, Park HS, Jung YS. Postoperative stability for surgery-first approach using intraoral vertical ramus osteotomy: 12 month follow-up. Br J Oral Maxillofac Surg. 2014;52(6):539-44.	Excluded by title
248	King PA. Management of hypodontia. British Dental Journal. 2006;201(8):525.	Excluded by title
249	Kinici R, Ieri H, Tz HH, Altug AT. Dentoalveolar distraction osteogenesis for rapid orthodontic canine retraction. Journal of Oral and Maxillofacial Surgery. 2002;60(4):389-94.	Excluded by title
250	Kinzinger G, Frye L, Diedrich P. Class II treatment in adults: Comparing camouflage orthodontics, dentofacial orthopedics and orthognathic surgery - A cephalometric study to evaluate various therapeutic effects. Journal of Orofacial Orthopedics. 2009;70(1):63-91.	Excluded by title
251	Kinzinger G, Savvaidis S, Gulden N, Ludwig B, Knosel M, Lisson J. Effects of two different functional appliances on root development of posterior teeth: activator vs. bite-jumping appliance. J Orofac Orthop. 2010;71(3):235-45.	Excluded by title
252	Kisely S, Howell K, Green J. Pathways to orthodontic care. J Public Health Med. 1997;19(2):148-55.	Excluded by title
253	Kiyak HA, McNeill RW, West RA. The emotional impact of orthognathic surgery and conventional orthodontics. Am J Orthod. 1985;88(3):224-34.	Excluded by title
254	Kjellberg H. Craniofacial growth in juvenile chronic arthritis. Acta Odontologica Scandinavica. 1998;56(6):360-5.	Excluded by title
255	Klages U, Rost F, Wehrbein H, Zentner A. Perception of occlusion, psychological impact of dental esthetics, history of orthodontic treatment and their relation to oral health in naval recruits. Angle Orthod. 2007;77(4):675-80.	Excluded by title
256	Klocke A, Korbmacher H, Kahl-Nieke B. The current status of interdisciplinary cooperation in myofunctional therapy - The speech therapist's point of view. Sprache Stimme Gehor. 2000;24(1):38-43.	Excluded by title
257	Knosel M, Eckstein A, Helms HJ. Durability of esthetic improvement following Icon resin infiltration of multibracket-induced white spot lesions compared with no therapy over 6 months: a single-center, split-mouth, randomized clinical trial. Am J Orthod Dentofacial Orthop. 2013;144(1):86-96.	Excluded by title
258	Knosel M, Jung K, Kinzinger G, Bauss O, Engelke W. A controlled evaluation of oral screen effects on intra-oral pressure curve characteristics. Eur J Orthod. 2010;32(5):535-41.	Excluded by title
259	Knosel M, Klang E, Helms HJ, Wiechmann D. Occurrence and severity of enamel decalcification adjacent to bracket bases and sub-bracket lesions during orthodontic treatment with two different lingual appliances. Eur J Orthod. 2016;38(5):485-92.	Excluded by title
260	Ko EW, Hsu SS, Hsieh HY, Wang YC, Huang CS, Chen YR. Comparison of progressive cephalometric changes and postsurgical stability of skeletal Class III correction with and without presurgical orthodontic treatment. J Oral Maxillofac Surg. 2011;69(5):1469-77.	Excluded by title

261	Ko EW, Huang CS, Chen YR. Characteristics and corrective outcome of face asymmetry by orthognathic surgery. J Oral Maxillofac Surg. 2009;67(10):2201-9.	Excluded by title
262	Ko EW, Lin SC, Chen YR, Huang CS. Skeletal and dental variables related to the stability of orthognathic surgery in skeletal Class III malocclusion with a surgery-first approach. J Oral Maxillofac Surg. 2013;71(5):e215-23.	Excluded by title
263	Kochar GD, Chakranarayan A, Londhe SM, Varghese B, Jayan B, Chopra SS, et al. Management of Skeletal Class II Malocclusion by Surgery-First Approach. Journal of Craniofacial Surgery. 2017;28(1):E40-E3.	Excluded by title
264	Konopka T, Lella A, Stankiewicz-Szałapska A, Zapała J. Tobacco smoking among dentists in Poland. Polish Annals of Medicine. 2017;24(1):24-30.	Excluded by title
265	Kucera J, Marek I. Unexpected complications associated with mandibular fixed retainers: A retrospective study. Am J Orthod Dentofacial Orthop. 2016;149(2):202-11.	Excluded by title
266	Kuhlefelt M, Laine P, Suominen AL, Lindqvist C, Thoren H. Smoking as a significant risk factor for infections after orthognathic surgery. J Oral Maxillofac Surg. 2012;70(7):1643-7.	Excluded by title
267	Kumar S, Srivastava A, Sharma A, Garg A, Kumar S. Periodontal intervention in Speedy orthodontics-a case report. Journal of Clinical and Diagnostic Research. 2016;10(1):15-6.	Excluded by title
268	Kuroda S, Murakami K, Morishige Y, Takano-Yamamoto T. Severe Class II malocclusion with facial asymmetry treated with intraoral vertico-sagittal ramus osteotomy and LeFort I osteotomy. Am J Orthod Dentofacial Orthop. 2009;135(6):809-19.	Excluded by title
269	Kuroedova VD, Kuroedova KL, Karasiunok AE. [Improvement of orthodontic treatment outcomes in 6-9 years old children]. Stomatologiia (Mosk). 2014;93(4):55-7.	Excluded by title
270	Kurt G, Iseri H, Kisnisci R. Rapid tooth movement and orthodontic treatment using dentoalveolar distraction (DAD). Long-term (5 years) follow-up of a Class II case. Angle Orthod. 2010;80(3):597-606.	Excluded by title
271	Kushimoto K, Endo M, Mineta M, Mabuchi H, Hioki S, Niwa K. A case of Class II malocclusion associated with a deeply impacted maxillary central incisor. Gifu Shika Gakkai Zasshi. 1990;17(1):310-9.	Excluded by title
272	Kwon Y-W, Bayome M, Park JU. Stability After Bilateral Sagittal Split Osteotomy With Rigid Internal Fixation in Surgery-First Approach. Journal of Oral and Maxillofacial Surgery. 2016;74(4).	Excluded by title
273	Lang G, Alfter G, Goz G, Lang GH. Retention and stability--taking various treatment parameters into account. J Orofac Orthop. 2002;63(1):26-41.	Excluded by title
274	Lang R, Ramaciotti D. [Functional bucco-dental restoration cost for adolescents aged 16 to 20 in the Geneva resident population (survey 1970-72). I. -- Description of estimates (author's transl)]. Rev Epidemiol Sante Publique. 1977;25(1):41-65.	Excluded by title
275	Larson BE, Lee NK, Jang MJ, Yun PY, Kim JW, Kim YK. Comparing Stability of Mandibular Setback Versus 2-Jaw Surgery in Class III Patients With Minimal Presurgical Orthodontics. J Oral Maxillofac Surg. 2017;75(6):1240-8.	Excluded by title
276	Leandro de Oliveira W, Saga AY, Ignacio SA, Rodrigues Justino EJ, Tanaka OM. Comparative study between different groups of esthetic component of the Index of Orthodontic Treatment Need and eye tracking. Am J Orthod Dentofacial Orthop. 2019;156(1):67-74.	Excluded by title
277	Lee CH, Mo JH, Choi IJ, Lee HJ, Seo BS, Kim DY, et al. The mandibular advancement device and patient selection in the treatment of obstructive sleep apnea. Arch Otolaryngol Head Neck Surg. 2009;135(5):439-44.	Excluded by title
278	Lee CH, Park HH, Seo BM, Lee SJ. Modern trends in Class III orthognathic treatment: A time series analysis. Angle Orthod. 2017;87(2):269-78.	Excluded by title
279	Lee J, Miyazawa K, Tabuchi M, Kawaguchi M, Shibata M, Goto S. Midpalatal miniscrews and high-pull headgear for anteroposterior and vertical anchorage control: cephalometric comparisons of treatment changes. Am J Orthod Dentofacial Orthop. 2013;144(2):238-50.	Excluded by title
280	Lee JK, Chung KR, Baek SH. Treatment outcomes of orthodontic treatment, corticotomy-assisted orthodontic treatment, and anterior segmental osteotomy for bimaxillary dentoalveolar protrusion. Plastic and Reconstructive Surgery. 2007;120(4):1027-36.	Excluded by title
281	Lee M, Strand M. Ehlers–Danlos syndrome in a young woman with anorexia nervosa and complex somatic symptoms. International Journal of Eating Disorders. 2018;51(3):281-4.	Excluded by title
282	Lee RY, Artun J, Alonzo TA. Are dental anomalies risk factors for apical root resorption in orthodontic patients? Am J Orthod Dentofacial Orthop. 1999;116(2):187-95.	Excluded by title
283	Lee SJ, Jang SY, Chun YS, Lim WH. Three-dimensional analysis of tooth movement after intrusion of a supraerupted molar using a mini-implant with partial-fixed orthodontic appliances. Angle Orthod. 2013;83(2):274-9.	Excluded by title
284	Leite FPP, Devito KL, Chandretti PCdS, Ribeiro WAL. Reabsorção radicular apical: relato de caso clínico. Odonto (Säo Bernardo do Campo). 2011;19(37):125-33.	Excluded by title
285	Lekic P, Kenny D, Moe HK, Barrett E, McCulloch CAG. Relationship of clonogenic capacity to plating efficiency and vital dye staining of human periodontal ligament cells: Implications for tooth replantation. Journal of Periodontal Research. 1996;31(4):294-300.	Excluded by title
286	Lempesi E, Pandis N, Fleming PS, Mavragani M. A comparison of apical root resorption after orthodontic treatment with surgical exposure and traction of maxillary impacted canines versus that without impactions. Eur J Orthod 2014;36(6):690-7.	Excluded by title
287	Levander E, Malmgren O, Stenback K. Apical root resorption during orthodontic treatment of patients with multiple aplasia: a study of maxillary incisors. Eur J Orthod. 1998;20(4):427-34.	Excluded by title
288	Li Y-Y, Zhou Y-H, Lin J-X. [Intruding upper incisors using mini-screw anchorage in patients with gummy smile]. Zhonghua kou qiang yi xue za zhi = Zhonghua kouqiang yixue zazhi = Chinese journal of stomatology. 2009;44(8):449-53.	Excluded by title

289	Liao YF, Chiu YT, Huang CS, Ko EW, Chen YR. Presurgical orthodontics versus no presurgical orthodontics: treatment outcome of surgical-orthodontic correction for skeletal class III open bite. Plast Reconstr Surg. 2010;126(6):2074-83.	Excluded by title
290	Lin M-H. A cephalometric analysis of the craniofacial growth in females with unilateral cleft lip and palate. Shikwa Gakuho. 2001;101(8):755-74.	Excluded by title
291	Liou EJ, Chang PM. Apical root resorption in orthodontic patients with en-masse maxillary anterior retraction and intrusion with miniscrews. Am J Orthod Dentofacial Orthop. 2010;137(2):207-12.	Excluded by title
292	Littlewood SJ, Millett DT, Doubleday B, Bearn DR, Worthington HV. Retention procedures for stabilising tooth position after treatment with orthodontic braces. Cochrane Database of Systematic Reviews. 2016(1).	Excluded by title
293	Liu JK, Hsiao CK, Chen HA, Tsai MY. Orthodontic correction of a mandibular first molar deeply impacted by an odontoma: a case report. Quintessence Int. 1997;28(6):381-5.	Excluded by title
294	Liu W, Zhou Y, Wang X, Liu D, Zhou S. Effect of maxillary protraction with alternating rapid palatal expansion and constriction vs expansion alone in maxillary retrusive patients: a single-center, randomized controlled trial. Am J Orthod Dentofacial Orthop. 2015;148(4):641-51.	Excluded by title
295	Lossdorfer S, Gotz W, Jager A. Parathyroid hormone modifies human periodontal ligament cell proliferation and survival in vitro. J Periodontal Res. 2006;41(6):519-26.	Excluded by title
296	Luo QY, Liang Y, Huang GX. [Clinical research of Bite-bumper combined with fixed appliance in treatment of lingual tipping deep bite]. Hua Xi Kou Qiang Yi Xue Za Zhi. 2009;27(1):64-7.	Excluded by title
297	Luther F, Morris DO, Hart C. Orthodontic preparation for orthognathic surgery: how long does it take and why? A retrospective study. Br J Oral Maxillofac Surg. 2003;41(6):401-6.	Excluded by title
298	Luther F, Morris DO, Karnezi K. Orthodontic treatment following orthognathic surgery: how long does it take and why? A retrospective study. J Oral Maxillofac Surg. 2007;65(10):1969-76.	Excluded by title
299	Ma Z, Xu G, Yang C, Xie Q, Shen Y, Zhang S. Efficacy of the technique of piezoelectric corticotomy for orthodontic traction of impacted mandibular third molars. Br J Oral Maxillofac Surg. 2015;53(4):326-31.	Excluded by title
300	Madani AS, Abdollahian E, Khiavi HA, Radvar M, Foroughipour M, Asadpour H, et al. The efficacy of gabapentin versus stabilization splint in management of sleep bruxism. J Prosthodont. 2013;22(2):126-31.	Excluded by title
301	Madlena M, Banoczy J, Gotz G, Marton S, Kaan M, Jr., Nagy G. Effects of amine and stannous fluorides on plaque accumulation and gingival health in orthodontic patients treated with fixed appliances: a pilot study. Oral Health Dent Manag. 2012;11(2):57-61.	Excluded by title
302	Makiguchi M, Funaki Y, Kato C, Okihara H, Ishida T, Yabushita T, et al. Effects of increased occlusal vertical dimension on the jaw-opening reflex in adult rats. Archives of Oral Biology. 2016;72:39-46.	Excluded by title
303	Mallineni SK, Yiu CK. A retrospective review of outcomes of dental treatment performed for special needs patients under general anaesthesia: 2-year follow-up. ScientificWorldJournal. 2014;2014:748353.	Excluded by title
304	Maltha JC, van Leeuwen EJ, Dijkman GEHM, Kuijpers-Jagtman AM. Incidence and severity of root resorption in orthodontically moved premolars in dogs. Orthodontics and Craniofacial Research. 2004;7(2):115-21.	Excluded by title
305	Marini I, Bartolucci ML, Bortolotti F, Innocenti G, Gatto MR, Alessandri Bonetti G. The effect of diode superpulsed low-level laser therapy on experimental orthodontic pain caused by elastomeric separators: a randomized controlled clinical trial. Lasers in medical science. 2015;30(1):35-41.	Excluded by title
306	Marino SD, Schiavone L, La Mendola FMC, Timpanaro T, Cucuzza ME, Greco F, et al. Hypoglossal nerve paralysis in a child after a dental procedure. Neurologia i Neurochirurgia Polska. 2018;52(3):406-9.	Excluded by title
307	Matsunaka E, Ueki S, Makimoto K. Impact of breastfeeding or bottle-feeding on surgical wound dehiscence after cleft lip repair in infants: a systematic review protocol. JBI Database System Rev Implement Rep. 2015;13(10):3-11.	Excluded by title
308	Mavragani M, Apisariyakul J, Brudvik P, Selvig KA. Is mild dental invagination a risk factor for apical root resorption in orthodontic patients? Eur J Orthod. 2006;28(4):307-12.	Excluded by title
309	McNamara JA, Hinton RJ, Hoffman DL. Histologic Analysis of Temporomandibular-Joint Adaptation to Protrusive Function in Young-Adult Rhesus-Monkeys (Macaca-Mulatta). Am J Orthod Dentofacial Orthop 1982;82(4):288-98.	Excluded by title
310	Mehdi H, Mohsin Girach M, Lakhani MJ. Anxiety levels in dental patients. Medical Forum Monthly. 2014;25(2):33-6.	Excluded by title
311	Mehrotra D, Dhasmanaa S, Kumar S. Management of temporomandibular ankylosis with temporal fascia inter-positional arthroplasty and distraction osteogenesis: report of 30 cases. J Long Term Eff Med Implants. 2009;19(2):139-48.	Excluded by title
312	Memon S, Fida M, Shaikh A. Comparison of Different Craniofacial Patterns with Pharyngeal Widths. Journal of the College of Physicians and Surgeons Pakistan. 2012;22(5):302-6.	Excluded by title
313	Menezes VAd, Cavalcanti LL, Albuquerque TCd, Garcia AFG, Leal RB. Respiração bucal no contexto multidisciplinar: percepção de ortodontistas da cidade do Recife orthodontists in the city of Recife, Brazil. Dental Press Journal of Orthodontics. 2011;16(6):84-92.	Excluded by title
314	Mengel R, Peleska B. A double-crown concept for restorations in patients with generalized aggressive periodontitis: Two case reports with 22- and 25-year follow-ups. International Journal of Periodontics and Restorative Dentistry. 2019;39(2):203-11.	Excluded by title
315	Mercado-Mamani S, Ríos-Villasis K. Tratamiento ortodóntlco quirúrglco de canino maxilar impactado con reabsorción radicular bilateral: reporte de caso. Rev estomatol Hered. 2013;23(2):83-8.	Excluded by title
316	Mercier S. European College of Orthodontics: Commission of affiliation and titularization. Int Orthod.	Excluded by title

	2017;15(2):278-96.	
317	Metalwala Z, Okunseri C, Fletcher S, Allareddy V. Orthognathic Surgical Outcomes in Patients With and Without Craniofacial Anomalies. J Oral Maxillofac Surg. 2018;76(2):436.e1-.e8.	Excluded by title
318	Miguel JAM, Gava ECB. Surgery first: An alternative approach to ortho-surgical patients. Progress in Orthodontics. 2012;13(3):246-59.	Excluded by title
319	Miller CC, Burnside G, Higham SM, Flannigan NL. Quantitative Light-induced Fluorescence-Digital as an oral hygiene evaluation tool to assess plaque accumulation and enamel demineralization in orthodontics. Angle Orthod. 2016;86(6):991-7.	Excluded by title
320	Milnes AR. Breastfeeding duration may be related to lower prevalence for posterior crossbite in the deciduous dentition. Journal of Evidence-Based Dental Practice. 2011;11(1):67-8.	Excluded by title
321	Min BK, Choi JY, Baek SH. Comparison of treatment duration between conventional three-stage method and surgery-first approach in patients with skeletal Class III malocclusion. J Craniofac Surg. 2014;25(5):1752-6.	Excluded by title
322	Miyao E, Nakayama M, Noda A, Miyao M, Arasaki H. Oral appliance therapy for a child with sleep apnea syndrome due to palatine tonsil hypertrophy. Sleep and Biological Rhythms. 2007;5(4):288-90.	Excluded by title
323	Moawad SG, Bouserhal J, Al-Munajed MK. Assessment of the efficiency of Erbium-YAG laser as an assistant method to rapid maxillary expansion: An in vivo study. Int Orthod. 2016;14(4):462-75.	Excluded by title
324	Mohammed H, Rizk MZ, Wafaie K, Ulhaq A, Almuzian M. Reminders improve oral hygiene and adherence to appointments in orthodontic patients: a systematic review and meta-analysis. Eur J Orthod 2019;41(2):204-13.	Excluded by title
325	Mohandesan H, Ravanmehr H, Valaei N. A radiographic analysis of external apical root resorption of maxillary incisors during active orthodontic treatment. Eur J Orthod. 2007;29(2):134-9.	Excluded by title
326	Moin K, Bishara SE. An evaluation of buccal shield treatment. A clinical and cephalometric study. Angle Orthod. 2007;77(1):57-63.	Excluded by title
327	Monga N, Kharbanda OP, Samrit V. Quantitative and qualitative assessment of anchorage loss during en-masse retraction with indirectly loaded miniscrews in patients with bimaxillary protrusion. Am J Orthod Dentofacial Orthop. 2016;150(2):274-82.	Excluded by title
328	Morelon JB, Meyer C, Parmentier J, Prost G, Weber E, Louvrier A. [Treatment of a unilateral Brodie's syndrome by surgical contraction of the maxillae]. J Stomatol Oral Maxillofac Surg. 2017;118(1):57-62.	Excluded by title
329	Muntean R, Komposch G, Steegmayer-Gilde G. Long-term stability of extraction therapy in anterior open bite. A case report. Journal of Orofacial Orthopedics. 2007;68(5):413-22.	Excluded by title
330	Murdock S, Lee JY, Guckes A, Wright JT. A costs analysis of dental treatment for ectodermal dysplasia. J Am Dent Assoc. 2005;136(9):1273-6.	Excluded by title
331	Nagalakshmi S, Sathish R, Priya K, Dhayanithi D. Changes in quality of life during orthodontic correction of midline diastema. Journal of Pharmacy and Bioallied Sciences. 2014;6(SUPPL. 1):S162-S4.	Excluded by title
332	Nahin J, Arshad F, Srinivas BV, Kumar S, Lokesh NK. The efficacy of low-level laser therapy on pain caused by placement of the first orthodontic archwire: A clinical study. Journal of Contemporary Dental Practice. 2018;19(4):450-5.	Excluded by title
333	Nair A, Prithviraj DR, Regish KM, Prithvi S. Custom milled zirconia implant supporting an ceramic zirconia restoration: A clinical report. Kathmandu University Medical Journal. 2013;11(44):328-31.	Excluded by title
334	Najt P, Nicoletti M, Chen HH, Hatch JP, Caetano SC, Sassi RB, et al. Anatomical measurements of the orbitofrontal cortex in child and adolescent patients with bipolar disorder. Neuroscience Letters. 2007;413(3):183-6.	Excluded by title
335	Nakamura M, Yanagita T, Matsumura T, Yamashiro T, Iida S, Kamioka H. A case of severe mandibular retrognathism with bilateral condylar deformities treated with Le Fort I osteotomy and two advancement genioplasty procedures. Korean Journal of Orthodontics. 2016;46(6):395-408.	Excluded by title
336	Nanda RS, Nanda SK. Considerations of Dentofacial Growth in Long-Term Retention and Stability - Is Active Retention Needed. Am J Orthod Dentofacial Orthop 1992;101(4):297-302.	Excluded by title
337	Nanjannawar LG, Girme TS, Agrawal JM, Agrawal MS, Fulari SG, Shetti SS, et al. Effect of mobile phone usage on nickel ions release and pH of saliva in patients undergoing fixed orthodontic treatment. Journal of Clinical and Diagnostic Research. 2017;11(9):ZC84-ZC7.	Excluded by title
338	Nedeljkovic N, Scepan I, Glisic B, Markovic E. [Dentaoalveolar changes in young adult patients with class II/1 malocclusion treated with the herbst appliance and an activator]. Vojnosanit Pregl. 2010;67(2):170-5.	Excluded by title
339	Ngan P, Yiu C. Evaluation of treatment and posttreatment changes of protraction facemask treatment using the PAR index. Am J Orthod Dentofacial Orthop. 2000;118(4):414-20.	Excluded by title
340	Nieri M, Crescini A, Rotundo R, Baccetti T, Cortellini P, Pini Prato GP. Factors affecting the clinical approach to impacted maxillary canines: A Bayesian network analysis. Am J Orthod Dentofacial Orthop. 2010;137(6):755-62.	Excluded by title
341	Nihtila A, Widstrom E. Heavy use of dental services among Finnish children and adolescents. Eur J Paediatr Dent. 2009;10(1):7-12.	Excluded by title
342	Nishimura F, Nojima K, Sueishi K, Yamaguchi H, Ikumoto H, Uchiyama T. Longitudinal evaluation and orthognathic surgical treatment of a patient with hemifacial microsomia. Shikwa Gakuho. 2004;104(1):93-102.	Excluded by title
343	Noori H, Hill DL, Shugars DA, Phillips C, White Jr RP. Third Molar Root Development and Recovery from Third Molar Surgery. Journal of Oral and Maxillofacial Surgery. 2007;65(4):680-5.	Excluded by title
344	Nouri M, Farzan A. Nonsurgical treatment of hemifacial microsomia: A case report. Iranian Red Crescent Medical Journal. 2015;17(11).	Excluded by title
345	O'Brien K, Wright J, Conboy F, Appelbe P, Bearn D, Caldwell S, et al. Prospective, multi-center study	Excluded by title

	of the effectiveness of orthodontic/orthognathic surgery care in the United Kingdom. Am J Orthod Dentofacial Orthop. 2009;135(6):709-14.	
346	Oh YH, Park HS, Kwon TG. Treatment effects of microimplant-aided sliding mechanics on distal retraction of posterior teeth. Am J Orthod Dentofacial Orthop. 2011;139(4):470-81.	Excluded by title
347	Ohba S, Tasaki H, Tobita T, Minamizato T, Kawasaki T, Motooka N, et al. Assessment of skeletal stability of intraoral vertical ramus osteotomy with one-day maxillary-mandibular fixation followed by early jaw exercise. Journal of Cranio-Maxillofacial Surgery. 2013;41(7):586-92.	Excluded by title
348	Okada W, Fukui T, Saito T, Ohkubo C, Hamada Y, Nakamura Y. Interdisciplinary treatment of an adult with complete bilateral cleft lip and palate. Am J Orthod Dentofacial Orthop. 2012;141(4 Suppl):S149-58.	Excluded by title
349	Oliveira FJd. Eficácia e segurança do laser cirúrgico de diodo em incisões circunvestibulares para osteotomia Le Fort I: ensaio clínico randomizado triplo cego. 2017:94-.	Excluded by title
350	Omidkhoda M, Radvar M, Azizi M, Hasanzadeh N. Piezopuncture-Assisted Canine Distalization in Orthodontic Patients: Two Case Reports. Journal of dentistry (Shiraz, Iran). 2018;19(1):74-82.	Excluded by title
351	Oncag G, Akyalcin S, Arikan F. The effectiveness of a single osteointegrated implant combined with pendulum springs for molar distalization. Am J Orthod Dentofacial Orthop. 2007;131(2):277-84.	Excluded by title
352	O'Reilly MT, De Jesus Vinas J, Hatch JP. Effectiveness of a sealant compared with no sealant in preventing enamel demineralization in patients with fixed orthodontic appliances: a prospective clinical trial. Am J Orthod Dentofacial Orthop. 2013;143(6):837-44.	Excluded by title
353	Ozdemir R, Baran CN, Karagoz MA, Dogan S. Place of sagittal split osteotomy in mandibular surgery. Journal of Craniofacial Surgery. 2009;20(2):349-55.	Excluded by title
354	Padmaprabha BP, Ponnambathayil SA, Aynipully H, Vinod M, Reghunathan DP. A precise method of measuring simultaneous intrusion and uprighting of mandibular molar using denta scan – A case report. Journal of Clinical and Diagnostic Research. 2015;9(7).	Excluded by title
355	Paim S, Modesto A, Cury JA, Thylstrup A. Development and control of caries lesions on the occlusal surface using a new in vivo caries model. Pesqui odontol bras. 2003;17(2):189-95.	Excluded by title
356	Pan J, Duan YZ, Zhang J, Tian MY, Lin Y. [Treatment of skeletal Class III adult patients with facemask protraction]. Zhonghua Kou Qiang Yi Xue Za Zhi. 2010;45(12):741-4.	Excluded by title
357	Papadimitriou A, Mousoulea S, Gkantidis N, Kloukos D. Clinical effectiveness of Invisalign® orthodontic treatment: a systematic review. Progress in Orthodontics. 2018;19(1).	Excluded by title
358	Park JH, Tai K, Sato Y. Management of Klippel-Feil syndrome combined with Turner syndrome: a case report. Int J Orthod Milwaukee. 2013;24(1):37-42.	Excluded by title
359	Park JH, Tai K, Sato Y. Orthodontic treatment of a patient with severe crowding and unilateral fracture of the mandibular condyle. Am J Orthod Dentofacial Orthop. 2016;149(6):899-911.	Excluded by title
360	Park KR, Kim SY, Park HS, Jung YS. Surgery-first approach on patients with temporomandibular joint disease by intraoral vertical ramus osteotomy. Oral Surg Oral Med Oral Pathol Oral Radiol. 2013;116(6):e429-36.	Excluded by title
361	Parkin NA, Deery C, Smith AM, Tinsley D, Sandler J, Benson PE. No difference in surgical outcomes between open and closed exposure of palatally displaced maxillary canines. Journal of Oral and Maxillofacial Surgery. 2012;70(9):2026-34.	Excluded by title
362	Passos JA, Siqueira K, Carelli J, Morais ND, Santana E, Topolski F, et al. Tratamento orto-cirúrgico da mordida aberta anterior relato de caso. Ortho Sci, Orthod sci pract. 2019;45(12):31-43.	Excluded by title
363	Patel HS, Managutti AM, Menat S, Agarwal A, Shah D, Patel J. Comparative evaluation of efficacy of physics forceps versus conventional forceps in orthodontic extractions: A prospective randomized split mouth study. Journal of Clinical and Diagnostic Research. 2016;10(7):ZC41-ZC5.	Excluded by title
364	Patel S, Fanshawe T, Bister D, Cobourne MT. Survival and success of maxillary canine autotransplantation: a retrospective investigation. Eur J Orthod. 2011;33(3):298-304.	Excluded by title
365	Pativetpinyo D, Supronsinchai W, Changsiripun C. Immediate effects of temporary bite-raising with light-cured orthodontic band cement on the electromyographic response of masticatory muscles. J Appl Oral Sci. 2018;26:e20170214.	Excluded by title
366	Paunonen J, Helminen M, Peltomaki T. Duration of orthognathic-surgical treatment. Acta Odontol Scand. 2017;75(5):372-5.	Excluded by title
367	Pereira S, Lavado N, Nogueira L, Lopez M, Abreu J, Silva H. Polymorphisms of genes encoding P2X7R, IL-1B, OPG and RANK in orthodontic-induced apical root resorption. Oral Diseases. 2014;20(7):659-67.	Excluded by title
368	Perović T, Aleksić I, Blažej Z. Orthodontic treatment of a severe unilateral open bite and crossbite, by palatal appliance with monolateral screw (By veltri). A case report. Vojnosanitetski Pregled. 2018;75(5):504-11.	Excluded by title
369	Pervin S, Rolland S, Taylor G. En masse versus two-step retraction of the anterior segment. Evid Based Dent. 2018;19(4):111-2.	Excluded by title
370	Phillips C, Essick G, Preisser JS, Turvey TA, Tucker M, Lin D. Sensory retraining after orthognathic surgery: effect on patients' perception of altered sensation. J Oral Maxillofac Surg. 2007;65(6):1162-73.	Excluded by title
371	Phillips C, Essick G, Zuniga J, Tucker M, Blakey IG. Qualitative Descriptors Used by Patients Following Orthognathic Surgery to Portray Altered Sensation. Journal of Oral and Maxillofacial Surgery. 2006;64(12):1751-60.	Excluded by title
372	Pietila I, Pietila T, Svedstrom-Oristo AL, Varrela J, Alanen P. Comparison of treatment costs and outcome in public orthodontic services in Finland. Eur J Orthod 2013;35(1):22-8.	Excluded by title
373	Pigatto PD, Ferrucci SM, Guzzi G, Sforza C. Rapid resolution of allergic contact dermatitis to nickel after intermaxillary fixation removal. British Journal of Oral and Maxillofacial Surgery. 2010;48(4):322.	Excluded by title

374	Pisek P, Manosudprasit M, Wangsrimongkol T, Keinprasit C, Wongpetch R. Treatment of a severe Class II Division 1 malocclusion combined with surgical miniscrew anchorage. Am J Orthod Dentofacial Orthop 2019;155(4):572-83.	Excluded by title
375	Postnikov MA, Stepanov GV, Malkina VD, Sharlanova SA, Ulyanova LG, inventors; Malkina V D, assignee. Method for orthodontic treatment of patients with congenital edentia of permanent teeth with application of basal "biomed" implants patent RU2638286-C1.	Excluded by title
376	Pouchain EC, Costa FWG, Bezerra TP, Soares ECS. Comparative efficacy of nimesulide and ketoprofen on inflammatory events in third molar surgery: A split-mouth, prospective, randomized, double-blind study. International Journal of Oral and Maxillofacial Surgery. 2015;44(7):876-84.	Excluded by title
377	Prakash S, Naik V, Dhanavel J. Multiple low flow vascular malformation with phleboliths - A case report. Journal of Young Pharmacists. 2017;9(3):446-50.	Excluded by title
378	Prasad GL, Hegde A, Divya S. Spinal Intramedullary Abscess Secondary to Dermal Sinus in Children. European Journal of Pediatric Surgery. 2019;29(3):229-38.	Excluded by title
379	Prasad GL, Kini P, Divya S. Central nervous system melioidosis in the pediatric age group: review. Childs Nerv Syst. 2017;33(6):1-6.	Excluded by title
380	Proc P, Szczepańska J, Herud A, Zubowska M, Fendler W, Młynarski W, et al. Dental caries among childhood cancer survivors. Medicine (United States). 2019;98(6).	Excluded by title
381	Proothi M, Grazina VJR, Gold AR. Chronic insomnia remitting after maxillomandibular advancement for mild obstructive sleep apnea: A case series. Journal of Medical Case Reports. 2019;13(1).	Excluded by title
382	Qafmolla A, Qafmolla R. Treatment of Malocclusion Class Ii with Functional Aparatus. International Journal of Ecosystems and Ecology Science-Ijees. 2016;6(1):123-6.	Excluded by title
383	Rabasco J, Vitelli O, Pietropaoli N, Rizzoli A, Castaldo R, Paolino M, et al. The duration of obstructive sleep apnea disease is predictive of efficacy or othodontic therapy in children. European Respiratory Journal. 2014;44.	Excluded by title
384	Rahimi-Nedjat RK, Sagheb K, Pabst A, Foersch M, Jacobs C, Vollandt L, et al. Diabetes and hyperglycemia as risk factors for postoperative outcome in maxillofacial surgery. J Surg Res. 2017;217:170-6.	Excluded by title
385	Rakhshan H, Rakhshan V. Pain and discomfort perceived during the initial stage of active fixed orthodontic treatment. Saudi Dental Journal. 2015;27(2):81-7.	Excluded by title
386	Rana M, Gellrich NC, Rana M, Piffko J, Kater W. Evaluation of surgically assisted rapid maxillary expansion with piezosurgery versus oscillating saw and chisel osteotomy - a randomized prospective trial. Trials. 2013;14:49.	Excluded by title
387	Ranta R. Forward Traction of the Maxilla with Cleft-Lip and Palate in Mixed and Permanent Dentitions. Journal of Cranio-Maxillofacial Surgery. 1989;17:20-2.	Excluded by title
388	Ranta R. Protraction of the cleft maxilla. Eur J Orthod 1988;10(1):215-22.	Excluded by title
389	Rashid H, Hussain SS. Frequency of impacted canines in orthodontic patients visiting KMDC. Medical Forum Monthly. 2016;27(7):18-21.	Excluded by title
390	Rashid S, Abidi YA, Hosein T. Success rate of resin bonded restorative dentistry bridges. Journal of the College of Physicians and Surgeons Pakistan. 2003;13(12):684-7.	Excluded by title
391	Ravera S, Castroflorio T, Garino F, Daher S, Cugliari G, Deregibus A. Maxillary molar distalization with aligners in adult patients: a multicenter retrospective study. Prog Orthod. 2016;17:12.	Excluded by title
392	Reinhart E, Reuther J, Michel C, Kubler N, Ordung R, Bosebeck H. [Perioperative antibiotic prophylaxis in orthodontic bone operations of the facial skull]. Mund Kiefer Gesichtschir. 1998;2(4):194-201.	Excluded by title
393	Ren Y, Maltha JC, Kuijpers-Jagtman AM. Tooth movement characteristics in relation to root resorption in young and adult rats. European Journal of Oral Sciences. 2007;115(6):449-53.	Excluded by title
394	Rennick LA, Campbell PM, Naidu A, Taylor RW, Buschang PH. Effectiveness of a novel topical powder on the treatment of traumatic oral ulcers in orthodontic patients: A randomized controlled trial. Angle Orthod. 2016;86(3):351-7.	Excluded by title
395	Ribeiro FV, Hirata DY, Reis AF, Santos VR, Miranda TS, Faveri M, et al. Open-flap versus flapless esthetic crown lengthening: 12-month clinical outcomes of a randomized controlled clinical trial. Journal of Periodontology. 2014;85(4):536-44.	Excluded by title
396	Richards MR, Fields HW, Jr., Beck FM, Firestone AR, Walther DB, Rosenstiel S, et al. Contribution of malocclusion and female facial attractiveness to smile esthetics evaluated by eye tracking. Am J Orthod Dentofacial Orthop. 2015;147(4):472-82.	Excluded by title
397	Richardson S, Selvaraj D, Khandeparker RV, Seelan NS, Richardson S. Tooth-Borne Anterior Maxillary Distraction for Cleft Maxillary Hypoplasia: Our Experience With 147 Patients. Journal of Oral and Maxillofacial Surgery. 2016;74(12):2504.e1-.e14.	Excluded by title
398	Richman CS. Dental Space Deficiency Syndrome: An Anthropological Perspective. Compend Contin Educ Dent. 2017;38(3):180-6.	Excluded by title
399	Riley P, Moore D, Ahmed F, Sharif MO, Worthington HV. Xylitol-containing products for preventing dental caries in children and adults. Cochrane Database of Systematic Reviews. 2015;2015(3).	Excluded by title
400	Rizzuti N, Scotti S. A case of hyperodontia with twenty-two supernumeraries: its surgical-orthodontic treatment. Am J Orthod Dentofacial Orthop. 1997;111(5):471-80.	Excluded by title
401	Roberts WE, Viecilli RF, Chang C, Katona TR, Paydar NH. Biology of biomechanics: Finite element analysis of a statically determinate system to rotate the occlusal plane for correction of a skeletal Class III open-bite malocclusion. Am J Orthod Dentofacial Orthop. 2015;148(6):943-55.	Excluded by title
402	Robiony M, Costa F, Politi M. Ultrasound endoscopic bone cutting for rapid maxillary expansion. Journal of Oral and Maxillofacial Surgery. 2014;72(5):980-90.	Excluded by title
403	Rodriguez Chessa J, Olate S, Chaves Netto HDdM, Barbosa JRdA, Mazzonetto R, Moreira RWF.	Excluded by title

	Hiperplasia fibrosa traumática asociada a implante ortodóncico: reporte de caso. Int j odontostomatol (Print). 2007;1(1):47-52.	
404	Rozendaal AM, Van Essen AJ, Te Meerman GJ, Bakker MK, Van Der Biezen JJ, Goorhuis-Brouwer SM, et al. Periconceptional folic acid associated with an increased risk of oral clefts relative to non-folate related malformations in the Northern Netherlands: A population based case-control study. European Journal of Epidemiology. 2013;28(11):875-87.	Excluded by title
405	Sabuncuoglu FA, Olmez H. Orthodontic treatment of a patient with unerupted maxillary central and lateral incisors and canine: a case report. Australian Orthodontic Journal. 2012;28(1):80-5.	Excluded by title
406	Saeed TB, Ashfaq M, Ahmed S, Qureshi R, Hussain SS, Pervez S. Frequency of hypodontia in a tertiary care hospital of Karachi. Medical Forum Monthly. 2014;25(5):42-5.	Excluded by title
407	Saglam-Aydinatay B, Taner T. Oral appliance therapy in obstructive sleep apnea: Long-term adherence and patients experiences. Med Oral Patol Oral Cir Bucal. 2018;23(1):e72-e7.	Excluded by title
408	Saito S, Ngan P, Saito M, Lanese R, Shanfeld J, Davidovitch Z. Interactive effects between cytokines on PGE production by human periodontal ligament fibroblasts in vitro. J Dent Res. 1990;69(8):1456-62.	Excluded by title
409	Sakamoto T, Sakamoto S, Harazaki M, Isshiki Y, Yamaguchi H. Orthodontic treatment for jaw deformities in cleft lip and palate patients with the combined use of an external-expansion arch and a facial mask. Bull Tokyo Dent Coll. 2002;43(4):223-9.	Excluded by title
410	Sakthi SV, Vikraman B, Shobana VR, Iyer SK, Krishnaswamy NR. Corticotomy-assisted retraction: an outcome assessment. Indian J Dent Res. 2014;25(6):748-54.	Excluded by title
411	Sarap LR, Dobrygina YV, Levchenko OG, Mansimov AV. Clinical rationale for choice of oral hygiene products for 6-12-year-old children on orthodontic treatment. Rossiiskii Vestnik Perinatologii i Pediatrii. 2011;56(3):75-8.	Excluded by title
412	Sari E, Kadioglu O, Ucar C, Altug HA. Prostaglandin E2 levels in gingival crevicular fluid during tooth- and bone-borne expansion. Eur J Orthod. 2010;32(3):336-41.	Excluded by title
413	Sathyapriya B, Srinivasan KR, Lakshmanan P, Selvi P. Facial nerve injury following TMJ surgery and its management by electrical stimulation - a case study. Biomedical and Pharmacology Journal. 2017;10(4):1855-61.	Excluded by title
414	Satoh K, Tsukagoshi T, Shimizu Y. Surgical refinement of the operative procedure for a minor degree of mandibular prognathism. Plast Reconstr Surg. 1996;98(4):740-6.	Excluded by title
415	Schendel SA, Linck DW. Mandibular distraction osteogenesis by sagittal split osteotomy and intraoral curvilinear distraction. Journal of Craniofacial Surgery. 2004;15(4):631-5.	Excluded by title
416	Schubert M, Proff P, Kirschneck C. Successful treatment of multiple bilateral impactions - a case report. Head Face Med. 2016;12(1):24.	Excluded by title
417	Schult H, Ziroldo S, Tocolini DG. Correção da sobremordida com aparelho autoligado potencializado pela toxina botulínica A - relato de caso. Ortho Sci, Orthod sci pract. 2015;8(29):66-73.	Excluded by title
418	Schuster G, Giese R. Retrospective clinical investigation of the impact of early treatment of children with Down's syndrome according to Castillo-Morales. J Orofac Orthop. 2001;62(4):255-63.	Excluded by title
419	Schuster G, Reiss-Ponitz U. The complex case--unforeseeable findings and interdisciplinary treatment. J Orofac Orthop. 2001;62(4):305-19.	Excluded by title
420	Sehgal A, Shetty S, Ashith MV, Jose NP, Mangal U. Efficacy of chlorhexidine varnish in patients undergoing multibracket fixed orthodontic treatment: A controlled clinical study. Biomedical and Pharmacology Journal. 2018;11(2):945-50.	Excluded by title
421	Semb G, Brattström V, Mølsted K, Prahl-Andersen B, Shaw WC. The Eurocleft study: Intercenter study of treatment outcome in patients with complete cleft lip and palate. Part 1: Introduction and treatment experience. Cleft Palate-Craniofacial Journal. 2005;42(1):64-8.	Excluded by title
422	Seo W, Kim SH, Chung KR, Nelson G. A pilot study of the osseointegration potential of a surface-treated mini-implant: bone contact of implants retrieved from patients. World J Orthod. 2009;10(3):202-10.	Excluded by title
423	Settineri S, Rizzo A, Ottanà A, Liotta M, Mento C. Dental aesthetics perception and eating behavior in adolescence. International Journal of Adolescent Medicine and Health. 2015;27(3):311-7.	Excluded by title
424	Shah M, Paramshivam G, Mehta A, Singh S, Chugh A, Prashar A, et al. Comparative assessment of conventional and light-curable fluoride varnish in the prevention of enamel demineralization during fixed appliance therapy: a split-mouth randomized controlled trial. Eur J Orthod. 2018;40(2):132-9.	Excluded by title
425	Shaikh AJ, Alvi AR. Comparison of cephalometric norms of esthetically pleasing faces. J Coll Physicians Surg Pak. 2009;19(12):754-8.	Excluded by title
426	Shen P, Chen X, Xie Q, Zhang S, Yang C. Assessment of Occlusal Appliance for the Reposition of Temporomandibular Joint Anterior Disc Displacement With Reduction. J Craniofac Surg. 2019;30(4):1140-3.	Excluded by title
427	Shi Z, Xie H, Wang P, Zhang Q, Wu Y, Chen E, et al. Oral hygiene care for critically ill patients to prevent ventilator-associated pneumonia. Cochrane Database of Systematic Reviews. 2013;2013(8).	Excluded by title
428	Shoreibah EA, Ibrahim SA, Attia MS, Diab MM. Clinical and radiographic evaluation of bone grafting in corticotomy-facilitated orthodontics in adults. J Int Acad Periodontol. 2012;14(4):105-13.	Excluded by title
429	Sikora T, Strzałkowska A. Orthodontic treatment of an adult patient with left-sided cleft lip and palate and a congenitally missing lateral incisor. Dental and Medical Problems. 2013;50(1):96-105.	Excluded by title
430	Sikorska-Bochinska J, Jamroszczyk K, Lagocka R, Lipski M, Nowicka A. [Dentinal hypersensivity after vertical stripping of enamel]. Ann Acad Med Stetin. 2009;55(2):65-7.	Excluded by title
431	Simon JS. European College of Orthodontics: Commission of Affiliation and Titularisation. Int Orthod. 2015;13(2):245-60.	Excluded by title
432	Singer E, Daskalogiannakis J, Russell KA, Mercado AM, Hathaway RR, Stoutland A, et al. Burden of	Excluded by title

	care of various infant orthopedic protocols for improvement of nasolabial esthetics in patients with CUCLP. Cleft Palate-Craniofacial Journal. 2018;55(9):1236-43.	
433	Singh P, Pandey A, Singh A, Ahuja T, Sharma S, Bhagalia SR, et al. Efficacy of intralesional placental extract, dexamethasone and hyaluronidase in treatment of oral submucous fibrosis:a comparative study. JK Practitioner. 2016;21(1-2):29-34.	Excluded by title
434	Singh V, Thepra M, Kirti S, Kumar P, Priya K. Dexmedetomidine as an Additive to Local Anesthesia: A Step to Development in Dentistry. Journal of Oral and Maxillofacial Surgery. 2018;76(10):2091.e1-.e7.	Excluded by title
435	Slavnic S, Marcusson A. Duration of orthodontic treatment in conjunction with orthognathic surgery. Swed Dent J. 2010;34(3):159-66.	Excluded by title
436	Smailiene D, Kavaliauskiene A, Pacauskiene I, Zasciurinskiene E, Bjerklin K. Palatally impacted maxillary canines: choice of surgical-orthodontic treatment method does not influence post-treatment periodontal status. A controlled prospective study. Eur J Orthod. 2013;35(6):803-10.	Excluded by title
437	Smailiene D, Kavaliauskiene A, Pacauskiene I. Posttreatment Status of Palatally Impacted Maxillary Canines Treated Applying 2 Different Surgical-Orthodontic Methods. Medicina-Lithuania. 2013;49(8):354-60.	Excluded by title
438	Sohn DS, Lee JK, An KM. Minor tooth movements using microimplant anchorage: case reports. Implant Dent. 2008;17(1):32-9.	Excluded by title
439	Soliman S, Ahmed M. The effect of orthognathic surgery on osteoprotegerin as immunological caliper of bone healing. Open Access Macedonian Journal of Medical Sciences. 2016;4(4):705-8.	Excluded by title
440	Sonesson M, Twetman S, Bondemark L. Effectiveness of high-fluoride toothpaste on enamel demineralization during orthodontic treatment-a multicenter randomized controlled trial. Eur J Orthod. 2014;36(6):678-82.	Excluded by title
441	Song GY, Li WR, Geng Z, Xu TM. [Agreement analysis of subjective evaluation of orthodontic treatment outcome]. Beijing Da Xue Xue Bao Yi Xue Ban. 2012;44(1):103-7.	Excluded by title
442	Stocker B, Willmann JH, Wilmes B, Vasudavan S, Drescher D. Wear-time recording during early Class III facemask treatment using TheraMon chip technology. Am J Orthod Dentofacial Orthop. 2016;150(3):533-40.	Excluded by title
443	Stoustrup P, Kristensen KD, Küseler A, Pedersen TK, Herlin T. Temporomandibular joint steroid injections in patients with juvenile idiopathic arthritis: An observational pilot study on the long-term effect on signs and symptoms. Pediatric Rheumatology. 2015;13(1).	Excluded by title
444	Stringer DE, Gilbert DH, Herford AS, Boyne PJ. A method of treating the patient with postpubescent juvenile rheumatoid arthritis. J Oral Maxillofac Surg. 2007;65(10):1998-2004.	Excluded by title
445	Suba Z, Hauser P, Garami M, Martonffy K, Szabó G, Szende B, et al. Skull base chordoma mimicking a preauricular neoplasm in a child: Clinicopathological features and biological behaviour. Journal of Cranio-Maxillofacial Surgery. 2007;35(1):35-8.	Excluded by title
446	Sudhakar V, Vinodhini TS, Mathan Mohan A, Srinivasan B, Rajkumar BK. The efficacy of different pre- and post-operative analgesics in the management of pain after orthodontic separator placement: A randomized clinical trial. Journal of Pharmacy and Bioallied Sciences. 2014;6(SUPPL. 1):S80-S4.	Excluded by title
447	Sun F, Ahmed A, Wang L, Dong M, Niu W. Comparison of oral microbiota in orthodontic patients and healthy individuals. Microbial Pathogenesis. 2018;123:473-7.	Excluded by title
448	Suzuki EY, Buranastidporn B, Ishii M. New Fixation Method for Maxillary Distraction Osteogenesis Using Locking Attachments. Journal of Oral and Maxillofacial Surgery. 2006;64(10):1553-60.	Excluded by title
449	Suzuki EY, Suzuki B. Removable splint with locking attachments for maxillary distraction osteogenesis with the RED system. International Journal of Oral and Maxillofacial Surgery. 2007;36(12):1153-7.	Excluded by title
450	Szarmach IJ, Kasacka I, Buczko P, Tankiewicz A, Pawlak D. Oral cavity status and IgE level in orthodontic patients. Adv Med Sci. 2006;51 Suppl 1:210-2.	Excluded by title
451	Szibor A, Jutila T, Makitie A, Aarnisalo A. Clinical Characteristics of Troublesome Pediatric Tinnitus. Clinical medicine insights Ear, nose and throat. 2017;10:1179550617736521-.	Excluded by title
452	Tai K, Park JH, Ikeda K, Nishiyama A, Sato Y. Severe facial asymmetry and unilateral lingual crossbite treated with orthodontics and 2-jaw surgery: 5-year follow-up. Am J Orthod Dentofacial Orthop 2012;142(4):509-23.	Excluded by title
453	Tai K, Park JH. Improvement of facial profile by nonextraction orthodontic treatment with temporary skeletal anchorage devices and visual treatment objectives. Am J Orthod Dentofacial Orthop. 2018;154(5):708-17.	Excluded by title
454	Takeuchi M, Tanaka E, Nonoyama D, Aoyama J, Tanne K. An adult case of skeletal open bite with a severely narrowed maxillary dental arch. Angle Orthod. 2002;72(4):362-70.	Excluded by title
455	Tanaka E, Iwabe T, Watanabe M, Kato M, Tanne K. An adolescent case of anterior open bite with masticatory muscle dysfunction. Angle Orthod. 2003;73(5):608-13.	Excluded by title
456	Tanaka E, Kawazoe A, Nakamura S, Ito G, Hirose N, Tanne Y, et al. An Adolescent Patient with Multiple Impacted Teeth. Angle Orthodontist. 2008;78(6):1110-8.	Excluded by title
457	Tanaka E, Ueki K, Kikuzaki M, Yamada E, Takeuchi M, Dalla-Bona D, et al. Longitudinal measurements of tooth mobility during orthodontic treatment using a Periotest. Angle Orthodontist. 2005;75(1):101-5.	Excluded by title
458	Tang AT, Bjorkman L, Lindback KF, Andlin-Sobocki A, Ekstrand J. Retrospective study of orthodontic bonding without liquid resin. Am J Orthod Dentofacial Orthop. 2000;118(3):300-6.	Excluded by title
459	Tapia CV, Batarce C, Amaro J, Hermosilla G, Rodas PI, Magne F. Microbiological characterisation of the colonisation by Candida sp in patients with orthodontic fixed appliances and evaluation of host responses in saliva. Mycoses. 2019;62(3):247-51.	Excluded by title
460	Teubner S, Schmidlin PR, Menghini G, Attin T, Baumgartner S. The Impact of Orthodontic Bands on the Marginal Periodontium of Maxillary First Molars: A Retrospective Cross-Sectional Radiographic	Excluded by title

	Analysis. Open Dentistry Journal. 2018;12:312-21.	
461	Throckmorton GS, Buschang PH, Hayasaki H, Pinto AS. Changes in the masticatory cycle following treatment of posterior unilateral crossbite in children. Am J Orthod Dentofacial Orthop 2001;120(5):521-9.	Excluded by title
462	Tome W, Yashiro K, Takada K. Orthodontic treatment of malocclusion improves impaired skillfulness of masticatory jaw movements. Angle Orthod. 2009;79(6):1078-83.	Excluded by title
463	Tsui VWK, Alkhal HA, Hou HM, Wong RWK, Rabie ABM. The modified two-by-one fixed orthodontic appliance for bodily movement of canine: A case report. Cases Journal. 2009;2(11).	Excluded by title
464	Tucker MR. Orthognathic surgery versus orthodontic camouflage in the treatment of mandibular deficiency. J Oral Maxillofac Surg. 1995;53(5):572-8.	Excluded by title
465	Tuncer C, Atac MS, Tuncer BB, Kaan E. Osteotomy assisted maxillary posterior impaction with miniplate anchorage. Angle Orthod. 2008;78(4):737-44.	Excluded by title
466	Tuncer NI, Koseoglu-Secgin C, Arman-Ozcirpici A. An unusual case of invasive cervical resorption after piezosurgery-assisted en masse retraction. Am J Orthod Dentofacial Orthop. 2019;156(1):137-47.	Excluded by title
467	Tuomilehto H, Bach N, Papadakis A, Remise C, Lavigne F, Rompre P, et al. The Effect of Orthodontic Expansion Treatment on Sleep Architecture in Healthy Young Adults. Sleep. 2009;32:A291-A.	Excluded by title
468	Uckan S, Soydan S, Veziroglu F, Ozcirpici AA. Transverse Reduction Genioplasty to Reduce Width of the Chin: Indications, Technique, and Results. Journal of Oral and Maxillofacial Surgery. 2010;68(6):1432-7.	Excluded by title
469	Ueki K, Marukawa K, Hashiba Y, Nakagawa K, Degerliyurt K, Yamamoto E. Changes in the duration of the chewing cycle in patients with skeletal class III with and without asymmetry before and after orthognathic surgery. J Oral Maxillofac Surg. 2009;67(1):67-72.	Excluded by title
470	Urban SD, Rebellato J, Keller EE. Intraoral maxillary quadrangular Le Fort II osteotomy: A long-term follow-up study. Journal of Oral and Maxillofacial Surgery. 2004;62(8):943-52.	Excluded by title
471	Uribe F, Adabi S, Janakiraman N, Allareddy V, Steinbacher D, Shafer D, et al. Treatment duration and factors associated with the surgery-first approach: a two-center study. Prog Orthod. 2015;16:29.	Excluded by title
472	Uribe F, Janakiraman N, Shafer D, Nanda R. Three-dimensional cone-beam computed tomography-based virtual treatment planning and fabrication of a surgical splint for asymmetric patients: surgery first approach. Am J Orthod Dentofacial Orthop. 2013;144(5):748-58.	Excluded by title
473	Uslu O, Erdem D. Report of a patient with a Class II occlusion using the Begg technique to move the first molars distally. World J Orthod. 2009;10(3):252-6.	Excluded by title
474	Uzuner FD, Darendeliler N. Dentoalveolar surgery techniques combined with orthodontic treatment: A literature review. European Journal of Dentistry. 2013;7(2):257-65.	Excluded by title
475	Vallon D, Nilner M, Söderfeldt B. Treatment Outcome in Patients with Craniomandibular Disorders of Muscular Origin: A 7-Year Follow-up. Journal of Orofacial Pain. 1998;12(3):210-8.	Excluded by title
476	Vardimon AD, Graber TM, Drescher D, Bourauel C. Rare earth magnets and impaction. Am J Orthod Dentofacial Orthop. 1991;100(6):494-512.	Excluded by title
477	Verdenik M, Ihan Hren N. Three-dimensional facial changes correlated with sagittal jaw movements in patients with class III skeletal deformities. British Journal of Oral and Maxillofacial Surgery. 2017;55(5):517-23.	Excluded by title
478	Vidqvist KL, Malin M, Varjolahti-Lehtinen T, Korpela MM. Disease activity of idiopathic juvenile arthritis continues through adolescence despite the use of biologic therapies. Rheumatology (United Kingdom). 2013;52(11):1999-2003.	Excluded by title
479	Villa MP, Castaldo R, Miano S, Paolino MC, Vitelli O, Tabarrini A, et al. Adenotonsillectomy and orthodontic therapy in pediatric obstructive sleep apnea. Sleep and Breathing. 2013:1-7.	Excluded by title
480	Villa MP, Castaldo R, Miano S, Paolino MC, Vitelli O, Tabarrini A, et al. Adenotonsillectomy and orthodontic therapy in pediatric obstructive sleep apnea. Sleep Breath. 2014;18(3):533-9.	Excluded by title
481	Villani S, Stellzig A, Komposch G. [Hypodontia: considerations on orthodontic therapy in agenesis of the permanent upper lateral incisor]. Minerva Stomatol. 1995;44(5):211-22.	Excluded by title
482	Villard NM, Patcas R. Does the decision to extract influence the development of gingival recessions? A retrospective long-term evaluation. J Orofac Orthop. 2015;76(6):476-92.	Excluded by title
483	Vishwanath M, Janakiraman N, Vaziri H, Nanda R, Uribe F. Autotransplantation: A biological treatment alternative for a patient after traumatic dental injury. Korean Journal of Orthodontics. 2018;48(2):125-30.	Excluded by title
484	Vitale MC, Modaffari C, Decembrino N, Zhou FX, Zecca M, Defabianis P. Preliminary study in a new protocol for the treatment of oral mucositis in pediatric patients undergoing hematopoietic stem cell transplantation (HSCT) and chemotherapy (CT). Lasers in Medical Science. 2017;32(6):1423-8.	Excluded by title
485	Vitalievichaveryanov S, Khairzamanova KA, Kudashkina NV, Hasanova SR, Tuygunov M. Efficiency of clinical application of phytofilm in treating patients with traumatic lesions of oral mucosa. International Journal of Pharmaceutical Research. 2018;10(4):611-5.	Excluded by title
486	Volk J, Kadivec M, Music MM, Ovsenik M. Three-dimensional ultrasound diagnostics of tongue posture in children with unilateral posterior crossbite. Am J Orthod Dentofacial Orthop 2010;138(5):608-12.	Excluded by title
487	von Arx T, Filippi A, Buser D. Splinting of traumatized teeth with a new device: TTS (Titanium Trauma Splint). Dental Traumatology. 2001;17(4):180-4.	Excluded by title
488	von Bremen J, Ruf S. Juvenile idiopathic arthritis-and now?: a systematic literature review of changes in craniofacial morphology. J Orofac Orthop. 2012;73(4):265-76.	Excluded by title
489	Vura N, Gaddipati R, Palla Y, Kumar P. An intraoral appliance to retract the protrusive premaxilla in bilateral cleft lip patients presenting late for primary lip repair. Cleft Palate-Craniofacial Journal.	Excluded by title

	2018;55(4):622-5.	
490	Wang Q, Chen W, Smales RJ, Peng H, Hu X, Yin L. Apical root resorption in maxillary incisors when employing micro-implant and J-hook headgear anchorage: a 4-month radiographic study. J Huazhong Univ Sci Technolog Med Sci. 2012;32(5):767-73.	Excluded by title
491	Wang YC, Ko EW, Huang CS, Chen YR, Takano-Yamamoto T. Comparison of transverse dimensional changes in surgical skeletal Class III patients with and without presurgical orthodontics. J Oral Maxillofac Surg. 2010;68(8):1807-12.	Excluded by title
492	Warren VT, Fisher AG, Rivera EM, Saha PT, Turner B, Reside G, et al. Buffered 1% Lidocaine With Epinephrine Is as Effective as Non-Buffered 2% Lidocaine With Epinephrine for Mandibular Nerve Block. J Oral Maxillofac Surg. 2017;75(7):1363-6.	Excluded by title
493	Watanabe Y, Sasaki R, Matsuno I, Akizuki T. Surgery-First Orthognathic Surgery for Severe Facial Asymmetry Combined With Mandibular Distraction Osteogenesis Using a Three-Dimensional Internal Distractor. J Craniofac Surg. 2019;30(1):39-46.	Excluded by title
494	Wendl B, Stampfl M, Muchitsch AP, Droschl H, Winsauer H, Walter A, et al. Long-term skeletal and dental effects of facemask versus chincup treatment in Class III patients: A retrospective study. Journal of Orofacial Orthopedics. 2017;78(4):293-9.	Excluded by title
495	Wey MC, Loh S, Doss JG, Abu Bakar AK, Kisely S. The oral health of people with chronic schizophrenia: A neglected public health burden. Aust N Z J Psychiatry. 2016;50(7):685-94.	Excluded by title
496	Whitehouse JA. Everyday uses of adult orthodontics. Dent Today. 2004;23(9):116, 8, 20.	Excluded by title
497	Wilcko WM. Rapid orthodontics with alveolar reshaping: Two case reports of decrowding. International Journal of Periodontics and Restorative Dentistry. 2001;21(1):9-19.	Excluded by title
498	Wilson KE, Welbury RR, Girdler NM. A randomised, controlled, crossover trial of oral midazolam and nitrous oxide for paediatric dental sedation. Anaesthesia. 2002;57(9):860-7.	Excluded by title
499	Won JH, Chun JS, Park YH, Kim SJ, Won YH. Treatment of pincer nail deformity using dental correction principles. Journal of the American Academy of Dermatology. 2018;78(5):1002-4.	Excluded by title
500	Wozniak K, Piatkowska D, Lipski M, Mehr K. Surface electromyography in orthodontics - a literature review. Medical Science Monitor. 2013;19:416-23.	Excluded by title
501	Wu J, Xu L, Liang C, Jiang J. Class III orthognathic surgical cases facilitated by accelerated osteogenic orthodontics: a preliminary report. Aust Orthod J. 2015;31(2):226-35.	Excluded by title
502	Yamaguchi K, Lonic D, Ko EWC, Lo LJ. An integrated surgical protocol for adult patients with hemifacial microsomia: Methods and outcome. PLoS ONE. 2017;12(8).	Excluded by title
503	Yao CC, Lai EH, Chang JZ, Chen I, Chen YJ. Comparison of treatment outcomes between skeletal anchorage and extraoral anchorage in adults with maxillary dentoalveolar protrusion. Am J Orthod Dentofacial Orthop. 2008;134(5):615-24.	Excluded by title
504	Yashiro K, Takada K. Improvements in smoothness of chewing cycles in adults with mandibular prognathism after surgery: a longitudinal study. J Oral Rehabil. 2013;40(6):418-28.	Excluded by title
505	Yeow VK, Chen YR, Su CP. Combining single- and double-tooth osteotomies with traditional orthognathic surgery. J Craniofac Surg. 1999;10(5):447-53.	Excluded by title
506	Yildirim D, Turkkahraman H, Yilmaz HH, Gungor AY, Ugan Y. Dentofacial characteristics of patients with rheumatoid arthritis. Clinical Oral Investigations. 2013;17(7):1677-83.	Excluded by title
507	Yilmaz HN, Karabiber G, Erverdi N. A Novel Approach for the Reconstruction of Premaxilla by Archwise Distraction in Bilateral Cleft Lip and Palate. J Craniofac Surg. 2019;30(1):e40-e3.	Excluded by title
508	Yoda T, Sato T, Abe T, Sakamoto I, Tomaru Y, Omura K, et al. Long-term results of surgical therapy for masticatory muscle tendon-aponeurosis hyperplasia accompanied by limited mouth opening. International Journal of Oral and Maxillofacial Surgery. 2009;38(11):1143-7.	Excluded by title
509	Yoshino S. The Changes of Mandibular Movement before and after Treatment of Mal Occlusion the Standardized Analysis on Lateral X-Ray Cephalogram in Orthodontic and Surgical Treatment Cases. Shikwa Gakuho. 1983;83(8):1055-97.	Excluded by title
510	Yu CC, Chen PH, Liou EJ, Huang CS, Chen YR. A Surgery-first approach in surgical-orthodontic treatment of mandibular prognathism--a case report. Chang Gung Med J. 2010;33(6):699-705.	Excluded by title
511	Yu HB, Mao LX, Wang XD, Fang B, Shen SG. The surgery-first approach in orthognathic surgery: a retrospective study of 50 cases. Int J Oral Maxillofac Surg. 2015;44(12):1463-7.	Excluded by title
512	Zain M, Rehman Khattak SU, Sikandar H, Shah SA, Fayyaz. Comparison of Anaesthetic Efficacy of 4% Articaine Primary Buccal Infiltration Versus 2% Lidocaine Inferior Alveolar Nerve Block in Symptomatic Mandibular First Molar Teeth. J Coll Physicians Surg Pak. 2016;26(1):4-8.	Excluded by title
513	Zanatta FB, Ardenghi TM, Antoniazzi RP, Pinto TM, Rosing CK. Association between gingivitis and anterior gingival enlargement in subjects undergoing fixed orthodontic treatment. Dental Press J Orthod. 2014;19(3):59-66.	Excluded by title
514	Zhao Y, Su YC, Jiang XY, Du J. [Clinical study on the stability of palatal implant anchorage]. Zhonghua Kou Qiang Yi Xue Za Zhi. 2005;40(6):463-7.	Excluded by title
515	Zheng X. [Use of interproximal enamel reduction in adult malocclusion patients with periodontitis]. Shanghai Kou Qiang Yi Xue. 2010;19(5):485-9.	Excluded by title
516	Zhou Y, Hu W, Fu M. [Pre- and post surgical orthodontic treatment of mandibular prognathism]. Zhonghua Kou Qiang Yi Xue Za Zhi. 1999;34(6):357-60.	Excluded by title
517	Zhou Y, Hu W, Sun Y. [Pre- and post-surgical orthodontic treatment for skeletal open bite]. Zhonghua Kou Qiang Yi Xue Za Zhi. 2001;36(3):225-8.	Excluded by title
518	Zhou YH, Sun YN, Hu W, Fu MK. [Application of straight wire appliance for pre- and post-surgical orthodontics]. Zhonghua Kou Qiang Yi Xue Za Zhi. 2004;39(6):509-12.	Excluded by title
519	Zhu SL, Wang Y, Wang DW. [First molar extraction in patients with crowding: cases analysis]. Zhonghua Kou Qiang Yi Xue Za Zhi. 2006;41(1):15-8.	Excluded by title

520	Zhu Y, Zou Y, Yu Q, Sun H, Mou S, Xu S, et al. Combined surgical-orthodontic treatment of patients with cleidocranial dysplasia: case report and review of the literature. Orphanet J Rare Dis. 2018;13(1):217.	Excluded by title
521	Alam MK. Laser assisted orthodontic tooth movement in saudi population: A randomized clinical trial. Bangladesh Journal of Medical Science. 2019;18(2):385-90.	Excluded by abstract
522	AlHammadi HA, Wilcko MT, Ferguson DJ. Severe mandibular crowding treated with nonextraction periodontally accelerated osteogenic orthodontics. International Journal of Periodontics and Restorative Dentistry. 2019;39(5):e188-e94.	Excluded by abstract
523	Al-Hasani NR, Al-Bustani AI, Ghareeb MM, Hussain SA. Clinical efficacy of locally injected calcitriol in orthodontic tooth movement. International Journal of Pharmacy and Pharmaceutical Sciences. 2011;3(SUPPL. 5):139-43.	Excluded by abstract
524	Alikhani M, Raptis M, Zoldan B, Sangsuwon C, Lee YB, Alyami B, et al. Effect of micro-osteoperforations on the rate of tooth movement. Am J Orthod Dentofacial Orthop. 2013;144(5):639-48.	Excluded by abstract
525	Al-Jundi A, Sakka S, Riba H, Ward T, Hanna R. Efficiency of er:Yag utilization in accelerating deep bite orthodontic treatment. Laser Therapy. 2018;27(3):193-202.	Excluded by abstract
526	Alkebsi A, Al-Maaitah E, Al-Shorman H, Abu Alhaija E. Three-dimensional assessment of the effect of micro-osteoperforations on the rate of tooth movement during canine retraction in adults with Class 11 malocclusion: A randomized controlled clinical trial. Am J Orthod Dentofacial Orthop 2018;153(6):771-85.	Excluded by abstract
527	Al-Omiri MK, Abu Alhaija ES. Factors affecting patient satisfaction after orthodontic treatment. Angle Orthod. 2006;76(3):422-31.	Excluded by abstract
528	AlSayed Hasan MMA, Sultan K, Hamadah O. Low-level laser therapy effectiveness in accelerating orthodontic tooth movement: A randomized controlled clinical trial. Angle Orthod. 2017;87(4):499-504.	Excluded by abstract
529	Amini F, Jafari A, Amini P, Sepasi S. Metal ion release from fixed orthodontic appliances - An in vivo study. Eur J Orthod 2012;34(1):126-30.	Excluded by abstract
530	Amini F, Rakhshan V, Sadeghi P. Effect of fixed orthodontic therapy on urinary nickel levels: a long-term retrospective cohort study. Biol Trace Elem Res. 2012;150(1-3):31-6.	Excluded by abstract
531	Artun J, Van 't Hullenaar R, Doppel D, Kuijpers-Jagtman AM. Identification of orthodontic patients at risk of severe apical root resorption. Am J Orthod Dentofacial Orthop. 2009;135(4):448-55.	Excluded by abstract
532	Baek SH, Ahn HW, Kwon YH, Choi JY. Surgery-first approach in skeletal class III malocclusion treated with 2-jaw surgery: evaluation of surgical movement and postoperative orthodontic treatment. J Craniofac Surg. 2010;21(2):332-8.	Excluded by abstract
533	Baldwin DK, King G, Ramsay DS, Huang G, Bollen A-M. Activation time and material stiffness of sequential removable orthodontic appliances. Part 3: Premolar extraction patients. Am J Orthod Dentofacial Orthop 2008;133(6):837-45.	Excluded by abstract
534	Barbosa IV, Ladewig VdM, Almeida-Pedrin RR, Cardoso MA, Santiago Junior JF, de Castro Ferreira Conti AC. The association between patient's compliance and age with the bonding failure of orthodontic brackets: a cross-sectional study. Progress in Orthodontics. 2018;19.	Excluded by abstract
535	Beck BW, Harris EF. Apical root resorption in orthodontically treated subjects: analysis of edgewise and light wire mechanics. Am J Orthod Dentofacial Orthop. 1994;105(4):350-61.	Excluded by abstract
536	Bilodeau JE. Nonsurgical treatment of a Class III patient with a lateral open-bite malocclusion. Am J Orthod Dentofacial Orthop. 2011;140(6):861-8.	Excluded by abstract
537	Bock NC, Reiser B, Ruf S. Class II subdivision treatment with the Herbst appliance. Angle Orthod. 2013;83(2):327-33.	Excluded by abstract
538	Bock NC, Santo C, Pancherz H. Facial profile and lip position changes in adult class II, division 2 subjects treated with the herbst-multi bracket appliance. A radiographic cephalometric pilot study. Journal of Orofacial Orthopedics. 2009;70(1):51-62.	Excluded by abstract
539	Bock NC, von Bremen J, Ruf S. Occlusal stability of adult Class II Division 1 treatment with the Herbst appliance. Am J Orthod Dentofacial Orthop. 2010;138(2):146-51.	Excluded by abstract
540	Boersma JG, Van Der Veen MH, Lagerweij MD, Bokhout B, Prahl-Andersen B. Caries prevalence measured with QLF after treatment with fixed orthodontic appliances: Influencing factors. Caries Research. 2005;39(1):41-7.	Excluded by abstract
541	Bos A, Kleverlaan CJ, Hoogstraten J, Prahl-Andersen B, Kuitert R. Comparing subjective and objective measures of headgear compliance. Am J Orthod Dentofacial Orthop. 2007;132(6):801-5.	Excluded by abstract
542	Boyd RL. Esthetic orthodontic treatment using the invisalign appliance for moderate to complex malocclusions. Journal of Dental Education. 2008;72(8):948-67.	Excluded by abstract
543	Bukhari OM, Sohrabi K, Tavares M. Factors affecting patients' adherence to orthodontic appointments. Am J Orthod Dentofacial Orthop. 2016;149(3):319-24.	Excluded by abstract
544	Caprioglio A, Bergamini C, Franchi L, Vercellini N, Zecca PA, Nucera R, et al. Prediction of Class II improvement after rapid maxillary expansion in early mixed dentition. Prog Orthod. 2017;18(1):9.	Excluded by abstract
545	Cassetta M, Altieri F. The influence of mandibular third molar germectomy on the treatment time of impacted mandibular second molars using brass wire: a prospective clinical pilot study. International Journal of Oral and Maxillofacial Surgery. 2017;46(7):905-11.	Excluded by abstract
546	Chen S, Chen YX, Hu J. [Pre- and post-surgical orthodontic treatment of mandibular asymmetry and prognathism]. Zhonghua Kou Qiang Yi Xue Za Zhi. 2005;40(1):38-41.	Excluded by abstract
547	Chen YJ, Chang HH, Huang CY, Hung HC, Lai EH, Yao CC. A retrospective analysis of the failure rate of three different orthodontic skeletal anchorage systems. Clin Oral Implants Res. 2007;18(6):768-75.	Excluded by abstract
548	Correia LP, Pinho MM, Manso MC. Motivation, perception of the impact and level of satisfaction with	Excluded by

	orthodontic treatment. Revista Portuguesa De Estomatologia Medicina Dentaria E Cirurgia Maxilofacial. 2016;57(4):247-51.	abstract
549	de Souza RA, de Oliveira AF, Pinheiro SM, Cardoso JP, Magnani MB. Expectations of orthodontic treatment in adults: the conduct in orthodontist/patient relationship. Dental Press J Orthod. 2013;18(2):88-94.	Excluded by abstract
550	Deguchi T, Honjo T, Fukunaga T, Miyawaki S, Roberts WE, Takano-Yamamoto T. Clinical assessment of orthodontic outcomes with the peer assessment rating, discrepancy index, objective grading system, and comprehensive clinical assessment. Am J Orthod Dentofacial Orthop 2005;127(4):434-43.	Excluded by abstract
551	Dehghani M, Fazeli F, Sattarzadeh AP. Efficiency and Duration of Orthodontic/Orthognathic Surgery Treatment. J Craniofac Surg. 2017;28(8):1997-2000.	Excluded by abstract
552	Delhaye S, Saba SB, Delatte M. [Prevention and treatment of dento-maxillary discrepancy]. Orthod Fr. 2006;77(2):267-81.	Excluded by abstract
553	Djeu G, Hayes C, Zawaideh S. Correlation between mandibular central incisor proclination and gingival recession during fixed appliance therapy. Angle Orthod. 2002;72(3):238-45.	Excluded by abstract
554	Ekizer A, Türker G, Uysal T, Güray E, Taşdemir Z. Light emitting diode mediated photobiomodulation therapy improves orthodontic tooth movement and miniscrew stability: A randomized controlled clinical trial. Lasers in Surgery and Medicine. 2016;48(10):936-43.	Excluded by abstract
555	El Namrawy MM, El Sharaby F, Bushnak M. Intrusive Arch versus Miniscrew-Supported Intrusion for Deep Bite Correction. Open Access Macedonian Journal of Medical Sciences. 2019;7(11):1841-6.	Excluded by abstract
556	El-Fateh T, Ruf S. Herbst treatment with mandibular cast splints--revisited. Angle Orthod. 2011;81(5):820-7.	Excluded by abstract
557	Elhaddaoui R, Benyahia H, Azeroual MF, Zaoui F, Razine R, Bahije L. Resorption of maxillary incisors after orthodontic treatment--clinical study of risk factors. Int Orthod. 2016;14(1):48-64.	Excluded by abstract
558	Feldmann I. Satisfaction with orthodontic treatment outcome. Angle Orthod. 2014;84(4):581-7.	Excluded by abstract
559	Finotti M, Del Torre M, Roberto M, Miotti FA. [Could the distalization of the mandibular molars be facilitated? A new therapeutic method]. Orthod Fr. 2009;80(4):371-8.	Excluded by abstract
560	Fontana M, Cozzani M, Caprioglio A. Soft tissue, skeletal and dentoalveolar changes following conventional anchorage molar distalization therapy in class II non-growing subjects: a multicentric retrospective study. Prog Orthod. 2012;13(1):30-41.	Excluded by abstract
561	Freitas BV, Abas Frazao MC, Dias L, Fernandes Dos Santos PC, Freitas HV, Bosio JA. Nonsurgical correction of a severe anterior open bite with mandibular molar intrusion using mini-implants and the multiloop edgewise archwire technique. Am J Orthod Dentofacial Orthop. 2018;153(4):577-87.	Excluded by abstract
562	Freitas DSd. Estabilidade das relações oclusais e da correção ortodôntica do apinhamento dentário anteroinferior: um estudo em curto e longo prazo. 2014:115-.	Excluded by abstract
563	Fu PS, Wang JC, Wu YM, Huang TK, Chen WC, Tseng YC, et al. Impacted mandibular second molars. Angle Orthod. 2012;82(4):670-5.	Excluded by abstract
564	Gandikota C, Venkata YP, Challa P, Juvvadi SR. Non-extraction treatment of severe crowding with pendulum appliance. Journal of Pharmacy and Bioallied Sciences. 2013;5(SUPPL. 2):S185-S9.	Excluded by abstract
565	Ghani S, Jabbar A, Shaikh IA, Memon AB, Naz E. Orthodontic treatment needs among population visiting the Liaquat University of Medical & Health Sciences Hospital. Medical Forum Monthly. 2015;26(4):2-4.	Excluded by abstract
566	Goddard R, Witherow H. Surgically assisted rapid palatal expansion (SARPE). British Journal of Oral and Maxillofacial Surgery. 2011;49(1):65-6.	Excluded by abstract
567	Göllner N, Winkler J, Göllner P, Gkantidis N. Effect of mandibular first molar mesialization on alveolar bone height: a split mouth study. Progress in Orthodontics. 2019;20(1).	Excluded by abstract
568	Gorman JC. Treatment of adults with lingual orthodontic appliances. Dent Clin North Am. 1988;32(3):589-620.	Excluded by abstract
569	Hägg U, Taranger J. Menarche and voice change as indicators of the pubertal growth spurt. Acta Odontologica Scandinavica. 1980;38(3):179-86.	Excluded by abstract
570	Hedayati Z, Hashemi SM, Zamiri B, Fattahi HR. Anchorage value of surgical titanium screws in orthodontic tooth movement. Int J Oral Maxillofac Surg. 2007;36(7):588-92.	Excluded by abstract
571	Hujoel P, Hollender L, Bollen A-M, Young JD, McGee M, Grosso A. Radiographs associated with one episode of orthodontic therapy. Journal of dental education. 2006;70(10):1061-5.	Excluded by abstract
572	Insoft M, King GJ, Keeling SD. The measurement of acid and alkaline phosphatase in gingival crevicular fluid during orthodontic tooth movement. Am J Orthod Dentofacial Orthop. 1996;109(3):287-96.	Excluded by abstract
573	Iosub Ciur MD, Zetu IN, Haba D, Viennot S, Bourgeois D, Andrian S. Evaluation of the Influence of Local Administration of Vitamin D on the Rate of Orthodontic Tooth Movement. Rev Med Chir Soc Med Nat Iasi. 2016;120(3):694-99.	Excluded by abstract
574	Islam ZU, Shaikh A, Fida M. Plaque index in multi-bracket fixed appliances. J Coll Physicians Surg Pak. 2014;24(11):791-5.	Excluded by abstract
575	Jaeken K, Cadenas De Llano-Pérula M, Lemiere J, Verdonck A, Fieuws S, Willems G. Difference and relation between adolescents' and their parents or caregivers' reported oral health-related quality of life related to orthodontic treatment: A prospective cohort study. Health and Quality of Life Outcomes. 2019;17(1).	Excluded by abstract
576	Jang SJ, Choi DS, Jang I, Jost-Brinkmann PG, Cha BK. Quantitative comparison of incisal tooth wear in patients receiving one-phase or two-phase treatment for skeletal Class III malocclusion with anterior crossbite. Angle Orthod. 2018;88(2):151-6.	Excluded by abstract

577	Janson G, Janson M, Nakamura A, de Freitas MR, Henriques JFC, Pinzan A. Influence of cephalometric characteristics on the occlusal success rate of Class II malocclusions treated with 2- and 4-premolar extraction protocols. Am J Orthod Dentofacial Orthop 2008;133(6):861-8.	Excluded by abstract
578	Janson G, Junqueira CH, Mendes LM, Garib DG. Influence of premolar extractions on long-term adult facial aesthetics and apparent age. Eur J Orthod. 2016;38(3):272-80.	Excluded by abstract
579	Janson M, Janson G, Santana E, de Castro RC, de Freitas MR. Orthodontic-surgical treatment of Class III malocclusion with extraction of an impacted canine and multi-segmented maxillary surgery. Am J Orthod Dentofacial Orthop. 2010;137(6):840-9.	Excluded by abstract
580	Janson M, Silva DAF. Mesialização de molares com ancoragem em mini-implantes. Rev dent press ortodon ortopedi facial. 2008;13(5):88-94.	Excluded by abstract
581	K AL-N, Abo-Zomor M, Alomari S. Changes in mandibular position in treated Class II division 2 malocclusions in growing and non-growing subjects. Aust Orthod J. 2016;32(1):73-81.	Excluded by abstract
582	Khanal A, Hu L, Chen L. Comparison of expression levels of RANKL and interleukin-17A in male and female orthodontic patients with and without appliances. Int J Periodontics Restorative Dent. 2015;35(2):e28-34.	Excluded by abstract
583	Kim SJ, Park KH, Park YG, Lee SW, Kang YG. Compressive stress induced the up-regulation of M-CSF, RANKL, TNF-alpha expression and the down-regulation of OPG expression in PDL cells via the integrin-FAK pathway. Arch Oral Biol. 2013;58(6):707-16.	Excluded by abstract
584	Kim SJ, Sung EH, Kim JW, Baik HS, Lee KJ. Mandibular molar protraction as an alternative treatment for edentulous spaces: Focus on changes in root length and alveolar bone height. J Am Dent Assoc. 2015;146(11):820-9.	Excluded by abstract
585	Kinzinger G, Czapka K, Ludwig B, Glasl B, Gross U, Lisson J. Effects of fixed appliances in correcting Angle Class II on the depth of the posterior airway space: FMA vs. Herbst appliance--a retrospective cephalometric study. J Orofac Orthop. 2011;72(4):301-20.	Excluded by abstract
586	Kleinerman V, Bergersen EO. Preventive and interceptive orthodontics for the 5 to 12 year-old. Functional appliances: the Nite-Guide and Occlus-o-Guide techniques. Refuat Hapeh Vehashinayim (1993). 2011;28(2):8-18, 72.	Excluded by abstract
587	Kook YA, Park JH, Bayome M, Sa'aed NL. Correction of severe bimaxillary protrusion with first premolar extractions and total arch distalization with palatal anchorage plates. Am J Orthod Dentofacial Orthop. 2015;148(2):310-20.	Excluded by abstract
588	Koutzoglou SI, Kostaki A. Effect of surgical exposure technique, age, and grade of impaction on ankylosis of an impacted canine, and the effect of rapid palatal expansion on eruption: A prospective clinical study. Am J Orthod Dentofacial Orthop 2013;143(3):342-52.	Excluded by abstract
589	Kukleva MP, Shetkova DG, Beev VH. Comparative age study of the risk of demineralization during orthodontic treatment with brackets. Folia Med (Plovdiv). 2002;44(1-2):56-9.	Excluded by abstract
590	Kumar KV, Umashankar K, Kumar DP, Kumar DP. Evaluation of canine retraction through distraction of the periodontal ligament: a clinical study. J Contemp Dent Pract. 2012;13(6):799-805.	Excluded by abstract
591	Kwon SY, Ahn HW, Kim SH, Park YG, Chung KR, Paik CH, et al. Antero-posterior lingual sliding retraction system for orthodontic correction of hyperdivergent Class II protrusion. Head Face Med. 2014;10:22.	Excluded by abstract
592	Laothong W, Cheng HC. Comparison of factors affecting orthodontic treatment motivation of Taiwanese and Thai patients in two hospitals. Journal of Dental Sciences. 2017;12(4):396-404.	Excluded by abstract
593	Levy PH. Clinical implications of mandibular repositioning and the concept of an alterable centric relation. Dental Clinics of North America. 1975;19(3):543-70.	Excluded by abstract
594	Leyder P, Altounian G, Chardain J, Quilichini J. Adjustable selective maxillary expansion combined with maxillomandibular surgery: A case report. Int Orthod. 2015;13(3):320-31.	Excluded by abstract
595	Linder-Aronson S. [The system and realization of orthodontic treatment in Sweden]. Stomatol DDR. 1977;27(12):808-15.	Excluded by abstract
596	Long H, Zhou Y, Xue J, Liao L, Ye N, Jian F, et al. The effectiveness of low-level laser therapy in accelerating orthodontic tooth movement: a meta-analysis. Lasers in Medical Science. 2015;30(3):1161-70.	Excluded by abstract
597	Maeda A, Soejima K, Ogura M, Ohmure H, Sugihara K, Miyawaki S. Orthodontic treatment combined with mandibular distraction osteogenesis and changes in stomatognathic function. Angle Orthod. 2008;78(6):1125-32.	Excluded by abstract
598	Mahmoudzadeh M, Farhadian M, Alijani S, Azizi F. Clinical comparison of two initial arch wires (A-NiTi and Heat Activated NiTi) for amount of tooth alignment and perception of pain: A randomized clinical trial. Int Orthod. 2018;16(1):60-72.	Excluded by abstract
599	Marshman Z, Eddaiki A, Bekker HL, Benson PE. Development and evaluation of a patient decision aid for young people and parents considering fixed orthodontic appliances. J Orthod. 2016;43(4):276-87.	Excluded by abstract
600	Martonffy AI. Oral health: orthodontic treatment. FP essentials. 2015;428:22-6.	Excluded by abstract
601	Mejare I, Bergman E, Grindefjord M. Hypomineralized molars and incisors of unknown origin: treatment outcome at age 18 years. Int J Paediatr Dent. 2005;15(1):20-8.	Excluded by abstract
602	Meuli S, Tecco S, Nota A, Gatto R, Caruso S. Clear aligners in pediatric age in a case of gingival recession due to malocclusion. Dental Cadmos. 2018;86(4):332-41.	Excluded by abstract
603	Miguel JAM, Zanardi G. Class III camouflage using skeletal anchorage and Pendex appliance. Progress in Orthodontics. 2011;12(1):73-83.	Excluded by abstract
604	Mills CM, McCulloch KJ. Posttreatment changes after successful correction of Class II malocclusions with the twin block appliance. Am J Orthod Dentofacial Orthop. 2000;118(1):24-33.	Excluded by abstract
605	Miresmaeili A, Basafa M, Shamsabadi RM, Farhadian N, Moghymbeigi A, Mollabashi V. Treatment	Excluded by

	decision analysis for palatally-displaced canines based on orthodontists' opinion and CBCT. International Orthodontics. 2017;15(4):625-39.	abstract
606	Mishra HA, Maurya RK. An approach with hybrid segmental mechanics. Journal of Clinical and Diagnostic Research. 2016;10(6):ZD18-ZD21.	Excluded by abstract
607	Mlynarska-Zduniak E, Pietrzak-Bilinska B, Kozlik D. [Extraction of permanent teeth in orthodontic treatment]. Czas Stomatol. 1990;43(9):561-6.	Excluded by abstract
608	Mommaerts MY, vande Vannet B. [Dental tours de force 5. Bimaxillary transverse distraction osteogenesis]. Ned Tijdschr Tandheelkd. 2004;111(2):40-3.	Excluded by abstract
609	Monea A, Monea M, Pop D, Beresescu G. The effect of low level laser therapy on orthodontic tooth movement. Optoelectronics and Advanced Materials-Rapid Communications. 2015;9(1-2):286-9.	Excluded by abstract
610	Moresca R, Fanderuff M, Fanderuff M, Casagrande C. Análise dos fatores que motivam pacientes jovens e adultos a buscarem tratamento ortodôntico. Ortho Sci, Orthod sci pract. 2017;10(39):273-82.	Excluded by abstract
611	Myrlund R, Dubland M, Keski-Nisula K, Kerosuo H. One year treatment effects of the eruption guidance appliance in 7- to 8-year-old children: a randomized clinical trial. Eur J Orthod. 2015;37(2):128-34.	Excluded by abstract
612	Nahhas RW, Valiathan M, Sherwood RJ. Variation in timing, duration, intensity, and direction of adolescent growth in the mandible, maxilla, and cranial base: the Fels longitudinal study. Anat Rec (Hoboken). 2014;297(7):1195-207.	Excluded by abstract
613	Navaneethan R, Sundari KKS, Ambika K. Periodontally accelerated osteogenic orthodontics (PAOO) assisted management of palatally impacted canine with five year follow up. Journal of Clinical and Diagnostic Research. 2017;11(10):ZD06-ZD8.	Excluded by abstract
614	Nedwed V, Miethke RR. Motivation, acceptance and problems of invisalign patients. J Orofac Orthop. 2005;66(2):162-73.	Excluded by abstract
615	Nishimura K, Nakao K, Aoki T, Fuyamada M, Saito K, Goto S. Orthodontic correction of a transposed maxillary canine and first premolar in the permanent dentition. Am J Orthod Dentofacial Orthop. 2012;142(4):524-33.	Excluded by abstract
616	Nobile CGA, Pavia M, Fortunato L, Angelillo IF. Prevalence and factors related to malocclusion and orthodontic treatment need in children and adolescents in Italy. European Journal of Public Health. 2007;17(6):637-41.	Excluded by abstract
617	Obilade OA, da Costa OO, Sanu OO. Patient/parent expectations of orthodontic treatment. Int Orthod. 2017;15(1):82-102.	Excluded by abstract
618	Orozco Estrada E, Gurrola Martínez B, Casasa Araujo A. Tracción de canino maxilar izquierdo impactado con botón bondeable, ligadura metálica y cadena elastomérica. Int j odontostomatol (Print). 2017;11(1):77-82.	Excluded by abstract
619	Parekh J, Counihan K, Fleming PS, Pandis N, Sharma PK. Effectiveness of part-time vs full-time wear protocols of Twin-block appliance on dental and skeletal changes: A randomized controlled trial. Am J Orthod Dentofacial Orthop. 2019;155(2):165-72.	Excluded by abstract
620	Pau-Bruchet L, Reynes C, Sabatier R, Galletti C. Statistical study on bracket debonding rate with the win lingual technique. Int Orthod. 2016;14(4):418-37.	Excluded by abstract
621	Pavoni C, Lombardo EC, Lione R, Faltin K, McNamara JA, Cozza P, et al. Treatment timing for functional jaw orthopaedics followed by fixed appliances: a controlled long-term study. Eur J Orthod 2018;40(4):430-6.	Excluded by abstract
622	Pender N, Samuels RH, Last KS. The monitoring of orthodontic tooth movement over a 2-year period by analysis of gingival crevicular fluid. Eur J Orthod. 1994;16(6):511-20.	Excluded by abstract
623	Pietila I, Pietila T, Svedstrom-Oristo AL, Varrela J, Alanen P. Acceptability of adolescents' occlusion in Finnish municipal health centres with differing timing of orthodontic treatment. Eur J Orthod. 2010;32(2):186-92.	Excluded by abstract
624	Rekka NCI, Sathiyawathie RS, Felcita S. Correlation between oral habits causing malocclusion in children. Drug Invention Today. 2019;11(4):822-4.	Excluded by abstract
625	Ren Y, Hazemeijer H, de Haan B, Qu N, de Vos P. Cytokine profiles in crevicular fluid during orthodontic tooth movement of short and long durations. J Periodontol. 2007;78(3):453-8.	Excluded by abstract
626	Ren Y, Vissink A. Cytokines in crevicular fluid and orthodontic tooth movement. European Journal of Oral Sciences. 2008;116(2):89-97.	Excluded by abstract
627	Sachan A, Chaturvedi TP. Orthodontic management of maxillary canine first premolar transposition - a conservative approach. Int J Orthod Milwaukee. 2013;24(4):59-62.	Excluded by abstract
628	Sadat-Marashi Z, Scolozzi P, Antonarakis GS. Perceptions of Young Adults Having Undergone Combined Orthodontic and Orthognathic Surgical Treatment: A Grounded Theory Approach. J Oral Maxillofac Surg. 2015;73(12):2391-8.	Excluded by abstract
629	Sandler J, Benson PE, Doyle P, Majumder A, O'Dwyer J, Speight P, et al. Palatal implants are a good alternative to headgear: a randomized trial. Am J Orthod Dentofacial Orthop. 2008;133(1):51-7.	Excluded by abstract
630	Savoldelli C, Chamorey E, Cizsek E, Lesne V, Manière-Ezvan A, Bettega G. Model to assess duration of distraction compared with degree of incisal crowding in symphyseal distraction osteogenesis. British Journal of Oral and Maxillofacial Surgery. 2013;51(8):887-91.	Excluded by abstract
631	Saxena R, Kumar PS, Upadhyay M, Naik V. A clinical evaluation of orthodontic mini-implants as intraoral anchorage for the intrusion of maxillary anterior teeth. World J Orthod. 2010;11(4):346-51.	Excluded by abstract
632	Scheurer PA, Firestone AR, Burgin WB. Perception of pain as a result of orthodontic treatment with fixed appliances. Eur J Orthod. 1996;18(4):349-57.	Excluded by abstract
633	Schott TC, Ludwig B. Quantification of wear-time adherence of removable appliances in young orthodontic patients in relation to their BMI: A preliminary study. Patient Preference and Adherence. 2014;8:1587-95.	Excluded by abstract

634	Seres L, Kocsis A. [Open-bite closure by intruding maxillary molars with skeletal anchorage]. Fogorv Sz. 2008;101(1):13-8.	Excluded by abstract
635	Shah N. Compliance with removable orthodontic appliances. Evid Based Dent. 2017;18(4):105-6.	Excluded by abstract
636	Shoreibah EA, Salama AE, Attia MS, Abu-Seida SM. Corticotomy-facilitated orthodontics in adults using a further modified technique. J Int Acad Periodontol. 2012;14(4):97-104.	Excluded by abstract
637	Shungin D, Olsson AI, Persson M. Orthodontic treatment-related white spot lesions: a 14-year prospective quantitative follow-up, including bonding material assessment. Am J Orthod Dentofacial Orthop. 2010;138(2):136.e1-8; discussion -7.	Excluded by abstract
638	Singh SP, Kumar V, Verma R, Singh S. Management of developing skeletal class III malocclusion in a prepubertal girl with prognathic mandible in late mixed dentition. Contemporary Clinical Dentistry. 2017;8(1):139-44.	Excluded by abstract
639	Singh SP, Utreja A, Chawla HS. Distribution of malocclusion types among thumb suckers seeking orthodontic treatment. J Indian Soc Pedod Prev Dent. 2008;26 Suppl 3:S114-7.	Excluded by abstract
640	Skomro P. [Orthodontic appliance made from elastic silicone: Clinical assessment and opinions of patients treated for malocclusion]. Roczniki Pomorskiej Akademii Medycznej w Szczecinie. 2000(46):293-304.	Excluded by abstract
641	Skomro P. [Orthodontic appliance made from silicone elastomer, evaluated clinically and from patient opinions after treatment for malocclusion]. Ann Acad Med Stetin. 2000;46:293-304.	Excluded by abstract
642	Smithpeter J, Covell D, Jr. Relapse of anterior open bites treated with orthodontic appliances with and without orofacial myofunctional therapy. Am J Orthod Dentofacial Orthop 2010;137(5):605-14.	Excluded by abstract
643	Sousa MV, Scanavini MA, Sannomiya EK, Velasco LG, Angelieri F. Influence of low-level laser on the speed of orthodontic movement. Photomed Laser Surg. 2011;29(3):191-6.	Excluded by abstract
644	Southard TE, Cohen ME, Ralls SA, Rouse LA. Effects of fixed-appliance orthodontic treatment on DMF indices. Am J Orthod Dentofacial Orthop. 1986;90(2):122-6.	Excluded by abstract
645	Struble BH, Huang GJ. Comparison of prospectively and retrospectively selected American Board of Orthodontics cases. Am J Orthod Dentofacial Orthop. 2010;137(1):6.e1-8; discussion 6-8.	Excluded by abstract
646	Sun XM, Teng L, Wang YH, Niu F, Tang Q, Wu GP, et al. Simultaneous occlusal orthodontics during mandibular distraction osteogenesis. Acta Academiae Medicinae Sinicae. 2006;28(3):399-401.	Excluded by abstract
647	Szarmach IJ, Szarmach J, Waszkiel D, Paniczko A. Assessment of periodontal status following the alignment of impacted permanent maxillary canine teeth. Adv Med Sci. 2006;51 Suppl 1:204-9.	Excluded by abstract
648	Talic NF, Alnahwi HH, Al-Faraj AS. Nickel and chromium levels in the saliva of a Saudi sample treated with fixed orthodontic appliances. Saudi Dental Journal. 2013;25(4):129-33.	Excluded by abstract
649	Tan TJ. Profile changes following orthodontic correction of bimaxillary protrusion with a preadjusted edgewise appliance. Int J Adult Orthodon Orthognath Surg. 1996;11(3):239-51.	Excluded by abstract
650	Tauheed S, Shaikh A, Fida M. Microaesthetics of The Smile: Extraction vs. Non-extraction. J Coll Physicians Surg Pak. 2012;22(4):230-4.	Excluded by abstract
651	Toroglu MS, Uzel E, Kayalioglu M, Uzel I. Asymmetric maxillary expansion (AMEX) appliance for treatment of true unilateral posterior crossbite. Am J Orthod Dentofacial Orthop 2002;122(2):164-73.	Excluded by abstract
652	Ulhaq A, Esmail Z, Kamaruddin A, Meadows S, Daus J, Vitale M, et al. Alignment efficiency and esthetic performance of 4 coated nickel-titanium archwires in orthodontic patients over 8 weeks: A multicenter randomized clinical trial. Am J Orthod Dentofacial Orthop. 2017;152(6):744-52.	Excluded by abstract
653	Ureturk SE, Sarac M, Firatli S, Can SB, Guven Y, Firatli E. The effect of low-level laser therapy on tooth movement during canine distalization. Lasers Med Sci. 2017;32(4):757-64.	Excluded by abstract
654	Uribe F, Agarwal S, Janakiraman N, Shafer D, Nanda R. Bidimensional dentoalveolar distraction osteogenesis for treatment efficiency. Am J Orthod Dentofacial Orthop. 2013;144(2):290-8.	Excluded by abstract
655	Uribe F, Padala S, Allareddy V, Nanda R. Patients', parents', and orthodontists' perceptions of the need for and costs of additional procedures to reduce treatment time. Am J Orthod Dentofacial Orthop. 2014;145(4 Suppl):S65-73.	Excluded by abstract
656	Vig PS, Vig KD. Decision analysis to optimize the outcomes for Class II Division 1 orthodontic treatment. Semin Orthod. 1995;1(3):139-48.	Excluded by abstract
657	Ward DE, Workman J, Brown R, Richmond S. Changes in arch width. A 20-year longitudinal study of orthodontic treatment. Angle Orthod. 2006;76(1):6-13.	Excluded by abstract
658	Wiechmann D, Vu J, Schwestka-Polly R, Helms HJ, Knosel M. Clinical complications during treatment with a modified Herbst appliance in combination with a lingual appliance. Head Face Med. 2015;11:31.	Excluded by abstract
659	Williams AC, Shah H, Sandy JR, Travess HC. Patients' motivations for treatment and their experiences of orthodontic preparation for orthognathic surgery. J Orthod. 2005;32(3):191-202.	Excluded by abstract
660	Woods M. Comprehensive treatment commenced in the mixed dentition and completed in the permanent dentition: an "early treatment" malocclusion. Aust Orthod J. 1999;15(4):251-9.	Excluded by abstract
661	Yildirim K, Saglam-Aydinatay B. Comparative assessment of treatment efficacy and adverse effects during nonextraction orthodontic treatment of Class I malocclusion patients with direct and indirect bonding: A parallel randomized clinical trial. Am J Orthod Dentofacial Orthop. 2018;154(1):26-34.e1.	Excluded by abstract
662	Yu H, Jiao F, Wang B, Shen SG. Piezoelectric decortication applied in periodontally accelerated osteogenic orthodontics. J Craniofac Surg. 2013;24(5):1750-2.	Excluded by abstract
663	Zafar Ul I, Shaikh A, Fida M. Dentoalveolar heights in skeletal class I normodivergent facial patterns. J Coll Physicians Surg Pak. 2012;22(1):5-9.	Excluded by abstract
664	Zhang J, Zhou S, Zheng H, Zhou Y, Chen F, Lin J. Magnetic bead-based salivary peptidome profiling analysis during orthodontic treatment durations. Biochem Biophys Res Commun. 2012;421(4):844-9.	Excluded by abstract
665	Amditis C, Smith LF. The duration of fixed orthodontic treatment: a comparison of two groups of	Excluded; missing

	patients treated using Edgewise brackets with 0.018" and 0.022" slots. Aust Orthod J. 2000;16(1):34-9.	fulltext
666	Baccetti T, Crescini A, Nieri M, Rotundo R, Pini Prato GP. Orthodontic treatment of impacted maxillary canines: an appraisal of prognostic factors. Prog Orthod. 2007;8(1):6-15.	Excluded; missing fulltext
667	Bai YX, Tian J, Zhou JM, Qi P, Yan YN, Wang BK. [Preliminary clinical application of Chinese-made invisible orthodontic technique]. Zhonghua Kou Qiang Yi Xue Za Zhi. 2008;43(8):464-7.	Excluded; missing fulltext
668	Duan Y, Zhang Y, Sun Y. [Treatment of Class II division 1 extraction cases by use of edgewise technique]. Zhonghua Kou Qiang Yi Xue Za Zhi. 2000;35(6):417-9.	Excluded; missing fulltext
669	Ikeda T, Yamaguchi M, Meguro D, Kasai K. Prediction and causes of open gingival embrasure spaces between the mandibular central incisors following orthodontic treatment. Aust Orthod J. 2004;20(2):87-92.	Excluded; missing fulltext
670	Ko-Kimura N, Kimura-Hayashi M, Yamaguchi M, Ikeda T, Meguro D, Kanekawa M, et al. Some factors associated with open gingival embrasures following orthodontic treatment. Aust Orthod J. 2003;19(1):19-24.	Excluded; missing fulltext
671	Kokitsawat S, Manosudprasit M, Godfrey K, Chatchaiwiwattana C. Clinical effects associated with miniscrews used as orthodontic anchorage. Australian Orthodontic Journal. 2008;24(2):134-9.	Excluded; missing fulltext
672	Koller S, Droschl H. [Orthodontic treatment from the point of view of the patients and their parents. 3. Attitude of patients and their parents toward orthodontic treatment after four weeks and after six months of treatment time]. Osterr Z Stomatol. 1977;74(12):428-40.	Excluded; missing fulltext
673	Lin JX, Gu Y. [Preliminary study of non-surgical treatment of severe Class III malocclusion in 18 patients of 12-20 years old]. Zhonghua Kou Qiang Yi Xue Za Zhi. 2004;39(2):91-6.	Excluded; missing fulltext
674	Liu X, Yao S, Yang S. [The clinical observation of treating Angle II (1) malocclusion in adults with Tip-Edge appliance]. Hua Xi Kou Qiang Yi Xue Za Zhi. 2003;21(2):121-3.	Excluded; missing fulltext
675	Lobb WK, Ismail AI, Andrews CL, Spracklin TE. Evaluation of orthodontic treatment using the Dental Aesthetic Index. Am J Orthod Dentofacial Orthop 1994;106(1):70-5.	Excluded; missing fulltext
676	Niwa K, Kushimoto K, Yamamaoto T. Mandibular first premolar teeth extraction in skeletal Class III malocclusion. Gifu Shika Gakkai Zasshi. 1990;17(1):330-8.	Excluded; missing fulltext
677	Otuyemi OD. Evaluation of orthodontic treatment outcome: a personal clinical audit using the PAR index (peer assessment rating). Afr Dent J. 1995;9:1-8.	Excluded; missing fulltext
678	Sakima MT, Davóglio AC, Oliveira CSBMd, Sakima AT, Sakima PRT, Sakima T. Meaw modificado: apresentação da técnica para a correção das mordidas abertas e na finalização dos tratamentos ortodônticos. Ortodontia. 2012;45(6):703-12.	Excluded; missing fulltext
679	Schmuth GP. [Treatment time--retention time--recurrence]. Fortschr Kieferorthop. 1966;27(1):22-31.	Excluded; missing fulltext
680	Wu JQ, Xu L, Liang C, Zou W, Bai YY, Jiang JH. [Class III surgical patients facilitated by accelerated osteogenic orthodontic treatment]. Zhonghua Kou Qiang Yi Xue Za Zhi. 2013;48(10):596-9.	Excluded; missing fulltext
681	Zhang J, Zhang WJ, Wang XX, Xu JG, Huang Y. [Orthodontic traction of impacted maxillary canine: a comparison of different ages]. Shanghai Kou Qiang Yi Xue. 2006;15(2):130-2.	Excluded; missing fulltext
682	Zhou Y, Zhang X, Xu T. [The effects of distalization of upper molars in Class II malocclusion by pendulum appliance]. Zhonghua Kou Qiang Yi Xue Za Zhi. 2000;35(6):413-6.	Excluded; missing fulltext
683	Fleming PS, Fedorowicz Z, Johal A, El-Angbawi A, Pandis N. Surgical adjunctive procedures for accelerating orthodontic treatment. Cochrane Database Syst Rev. 2015(6):Cd010572.	Excluded; review
684	Antelo OM, Meira TM, Iturralde A, Guimarães LK, Tanaka OM. Class ii, division 1 malocclusion treated with the andresen appliance followed by fixed orthodontics. World Journal of Dentistry. 2018;9(3):242-8.	Excluded; case report/ series
685	Ikegami T, Wong RW, Hagg U, Lee W, Hibino K. The Hybrid Orthodontic Treatment System (HOTS). World J Orthod. 2010;11(2):168-79.	Excluded; case report/ series
686	Jofre J, Montenegro J, Arroyo R. Rapid orthodontics with flapless piezoelectric corticotomies: first clinical experiences. Int j odontostomatol (Print). 2013;7(1):79-85.	Excluded; case report/ series
687	Juneja P, Chopra SS, Jayan BK. Self ligating lingual appliance. Medical journal, Armed Forces India. 2015;71(Suppl 2):S411-4.	Excluded; case report/ series
688	McNamara Jr JA. Dentofacial adaptations in adult patients following functional regulator therapy. American Journal of Orthodontics. 1984;85(1):57-71.	Excluded; case report/ series
689	Moresca R. Orthodontic treatment time: can it be shortened? Dental Press J Orthod. 2018;23(6):90-105.	Excluded; case report/ series
690	Roblee RD, Bolding SL, Landers JM. Surgically facilitated orthodontic therapy: a new tool for optimal interdisciplinary results. Compendium of continuing education in dentistry (Jamesburg, NJ : 1995). 2009;30(5):264-78.	Excluded; case report/ series
691	Tai K, Park JH, Tatamiya M, Kojima Y. Distal movement of the mandibular dentition with temporary skeletal anchorage devices to correct a Class III malocclusion. Am J Orthod Dentofacial Orthop. 2013;144(5):715-25.	Excluded; case report/ series
692	Yezdani AA. Alveolar bone shaping and augmentation -A prelude for rapid orthodontics. Biomedical and Pharmacology Journal. 2015;8SE:379-87.	Excluded; case report/ series
693	Meier B, Wiemer KB, Miethke RR. Invisalign--patient profiling. Analysis of a prospective survey. J Orofac Orthop. 2003;64(5):352-8.	Excluded; no fixed appliances
694	O'Brien K, Wright J, Conboy F, Appelbe P, Davies L, Connolly I, et al. Early treatment for Class II Division 1 malocclusion with the Twin-block appliance: a multi-center, randomized, controlled trial. Am J Orthod Dentofacial Orthop. 2009;135(5):573-9.	Excluded; no fixed appliances
695	Romanec C, Dragomir B, Bica C. The Prophylactic Orthodontic Treatment with Removable Appliances in Children. Revista De Chimie. 2018;69(3):693-6.	Excluded; no fixed appliances

696	Banks P, Wright J, O'Brien K. Incremental versus maximum bite advancement during twin-block therapy: a randomized controlled clinical trial. Am J Orthod Dentofacial Orthop. 2004;126(5):583-8.	Excluded; only one age group
697	Bertossi D, Vercellotti T, Podesta A, Nocini PF. Orthodontic microsurgery for rapid dental repositioning in dental malpositions. J Oral Maxillofac Surg. 2011;69(3):747-53.	Excluded; only one age group
698	Cuntan L, Maniu I, Neamtu ML. The evaluation of the efficacyand the duration of the orthodontic treatment related to its age of initiation. European Journal of Pediatrics. 2016;175(11):1847.	Excluded; only one age group
699	Dalci O, Altug AT, Memikoglu UT. Treatment effects of a twin-force bite corrector versus an activator in comparison with an untreated Class II sample: a preliminary report. Aust Orthod J. 2014;30(1):45-53.	Excluded; only one age group
700	D'Attilio M, Festa F, Filippakos A, Comparelli U, Tripodi D. Third Class Resolver: a retrospective analysis. Eur J Paediatr Dent. 2014;15(3):323-5.	Excluded; only one age group
701	DiBiase AT, Woodhouse NR, Papageorgiou SN, Johnson N, Slipper C, Grant J, et al. Effects of supplemental vibrational force on space closure, treatment duration, and occlusal outcome: A multicenter randomized clinical trial. Am J Orthod Dentofacial Orthop 2018;153(4):469-80.e4.	Excluded; only one age group
702	Gazit-Rappaport T, Haisraeli-Shalish M, Gazit E. Psychosocial reward of orthodontic treatment in adult patients. Eur J Orthod 2010;32(4):441-6.	Excluded; only one age group
703	Ireland AJ, Songra G, Clover M, Atack NE, Sherriff M, Sandy JR. Effect of gender and Frankfort mandibular plane angle on orthodontic space closure: a randomized controlled trial. Orthodontics & Craniofacial Research. 2016;19(2):74-82.	Excluded; only one age group
704	Janson G, Busato MC, Henriques JF, de Freitas MR, de Freitas LM. Alignment stability in Class II malocclusion treated with 2- and 4-premolar extraction protocols. Am J Orthod Dentofacial Orthop. 2006;130(2):189-95.	Excluded; only one age group
705	Lee R, Hwang S, Lim H, Cha JY, Kim KH, Chung CJ. Treatment satisfaction and its influencing factors among adult orthodontic patients. Am J Orthod Dentofacial Orthop. 2018;153(6):808-17.	Excluded; only one age group
706	McFadden WM, Engstrom C, Engstrom H, Anholm JM. A study of the relationship between incisor intrusion and root shortening. Am J Orthod Dentofacial Orthop 1989;96(5):390-6.	Excluded; only one age group
707	McNamara JA, Jr., Baccetti T, Franchi L, Herberger TA. Rapid maxillary expansion followed by fixed appliances: a long-term evaluation of changes in arch dimensions. Angle Orthod. 2003;73(4):344-53.	Excluded; only one age group
708	Melo AC, Carneiro LO, Pontes LF, Cecim RL, de Mattos JN, Normando D. Factors related to orthodontic treatment time in adult patients. Dental Press J Orthod. 2013;18(5):59-63.	Excluded; only one age group
709	Merwin D, Ngan P, Hagg U, Yiu C, Wei SH. Timing for effective application of anteriorly directed orthopedic force to the maxilla. Am J Orthod Dentofacial Orthop. 1997;112(3):292-9.	Excluded; only one age group
710	Pandis N, Nasika M, Polychronopoulou A, Eliades T. External apical root resorption in patients treated with conventional and self-ligating brackets. Am J Orthod Dentofacial Orthop 2008;134(5):646-51.	Excluded; only one age group
711	Richmond S, Ikonomou C, Williams B, Ramel S, Rolfe B, Kurol J. Orthodontic treatment standards in a public group practice in Sweden. Swed Dent J. 2001;25(4):137-44.	Excluded; only one age group
712	Ruf S, Pancherz H. Does bite-jumping damage the TMJ? A prospective longitudinal clinical and MRI study of Herbst patients. Angle Orthodontist. 2000;70(3):183-99.	Excluded; only one age group
713	Silva Filho OGd, Capelozza Filho L, Crosara KTB, Ozawa TO. Avaliação cefalométrica dos efeitos do aparelho herbst no tratamento da deficiência mandibular na dentadura permanente. Rev dent press ortodon ortopedi facial. 2007;12(6):101-18.	Excluded; only one age group
714	Taner T, Ciger S, Sencift Y. Evaluation of apical root resorption following extraction therapy in subjects with Class I and Class II malocclusions. Eur J Orthod 1999;21(5):491-6.	Excluded; only one age group
715	Uribe F, Davoody L, Mehr R, Jayaratne YSN, Almas K, Sobue T, et al. Efficiency of piezotome-corticision assisted orthodontics in alleviating mandibular anterior crowding-a randomized clinical trial. Eur J Orthod. 2017;39(6):595-600.	Excluded; only one age group
716	Wiedel AP, Bondemark L. Fixed versus removable orthodontic appliances to correct anterior crossbite in the mixed dentition--a randomized controlled trial. Eur J Orthod. 2015;37(2):123-7.	Excluded; only one age group
717	Wu J, Jiang JH, Xu L, Liang C, Bai Y, Zou W. A pilot clinical study of Class III surgical patients facilitated by improved accelerated osteogenic orthodontic treatments. Angle Orthod. 2015;85(4):616-24.	Excluded; only one age group
718	Yavuz MC, Sunar O, Buyuk SK, Kantarci A. Comparison of piezocision and discision methods in orthodontic treatment. Prog Orthod. 2018;19(1):44.	Excluded; only one age group
719	Yin K, Han E, Guo J, Yasumura T, Grauer D, Sameshima G. Evaluating the treatment effectiveness and efficiency of Carriere Distalizer: a cephalometric and study model comparison of Class II appliances. Progress in Orthodontics. 2019;20(1).	Excluded; only one age group
720	Amuk NG, Baysal A, Coskun R, Kurt G. Effectiveness of incremental vs maximum bite advancement during Herbst appliance therapy in late adolescent and young adult patients. Am J Orthod Dentofacial Orthop 2019;155(1):48-56.	Excluded; mixed age group
721	Anand M, Turpin DL, Jumani KS, Spiekerman CF, Huang GJ. Retrospective investigation of the effects and efficiency of self-ligating and conventional brackets. Am J Orthod Dentofacial Orthop 2015;148(1):67-75.	Excluded; mixed age group
722	Aragon MLC, Bichara LM, Flores-Mir C, Almeida G, Normando D. Efficiency of compensatory orthodontic treatment of mild Class III malocclusion with two different bracket systems. Dental Press J Orthod. 2017;22(6):49-55.	Excluded; mixed age group
723	Årtun J. A post treatment evaluation of multibonded lingual appliances in orthodontics. Eur J Orthod 1987;9(1):205-10.	Excluded; mixed age group
724	Atik E, Akarsu-Guven B, Kocadereli I. Soft tissue effects of three different Class II/1-camouflage treatment strategies. J Orofac Orthop. 2017;78(2):153-65.	Excluded; mixed age group
725	Azeem M, Ul Haq A, Ul Hamid W, Hayat MB, Khan DI, Ahmed A, et al. Efficiency of class III	Excluded; mixed

	malocclusion treatment with 2-premolar extraction and molar distalization protocols. Int Orthod. 2018;16(4):665-75.	age group
726	Baldo TdO. Eficiência do tratamento da má-oclusão de Classe II, subdivisão com extrações de três pré-molares e quatro pré-molares. 2010:143-.	Excluded; mixed age group
727	Basdra EK, Stellzig A, Komposch G. Extraction of maxillary second molars in the treatment of Class II malocclusion. Angle Orthod. 1996;66(4):287-92.	Excluded; mixed age group
728	Beckwith FR, Ackerman RJ, Jr., Cobb CM, Tira DE. An evaluation of factors affecting duration of orthodontic treatment. Am J Orthod Dentofacial Orthop. 1999;115(4):439-47.	Excluded; mixed age group
729	Bertl MH, Foltin A, Giannis K, Vasak C, Bernhart T, Strbac GD. Influence of repeat surgery on treatment time in the interdisciplinary management of impacted maxillary canines: A retrospective cohort study. J Craniomaxillofac Surg. 2016;44(7):843-7.	Excluded; mixed age group
730	Bhattacharya P, Bhattacharya H, Anjum A, Bhandari R, Agarwal DK, Gupta A, et al. Assessment of corticotomy facilitated tooth movement and changes in alveolar bone thickness - A ct scan study. Journal of Clinical and Diagnostic Research. 2014;8(10):ZC26-ZC30.	Excluded; mixed age group
731	Bichara LM, Aragon ML, Brandao GA, Normando D. Factors influencing orthodontic treatment time for non-surgical Class III malocclusion. J Appl Oral Sci. 2016;24(5):431-6.	Excluded; mixed age group
732	Bindayel NA, Alwadei A, Almosa N, Aasser W, Qazali A, Samran A, et al. Evaluation of bracket failure in relation to different factors in patients experiencing comprehensive orthodontic treatment: A retrospective cohort study. Journal of Oral Research. 2019;8(2):116-21.	Excluded; mixed age group
733	Buschang PH, Shaw SG, Ross M, Crosby D, Campbell PM. Comparative time efficiency of aligner therapy and conventional edgewise braces. Angle Orthod. 2014;84(3):391-6.	Excluded; mixed age group
734	Chaushu S, Becker T, Becker A. Impacted central incisors: factors affecting prognosis and treatment duration. Am J Orthod Dentofacial Orthop. 2015;147(3):355-62.	Excluded; mixed age group
735	Cousins AJ, Lewis HG, Viader PH. Changes in orthodontic treatment patterns within one orthodontic practice over a 15 year period. Br J Orthod. 1981;8(1):11-4.	Excluded; mixed age group
736	Crescini A, Nieri M, Buti J, Baccetti T, Pini Prato GP. Orthodontic and periodontal outcomes of treated impacted maxillary canines. Angle Orthod. 2007;77(4):571-7.	Excluded; mixed age group
737	Doshi-Mehta G, Bhad-Patil WA. Efficacy of low-intensity laser therapy in reducing treatment time and orthodontic pain: a clinical investigation. Am J Orthod Dentofacial Orthop. 2012;141(3):289-97.	Excluded; mixed age group
738	Faruqui S, Fida M, Shaikh A. Factors Affecting Treatment Duration - A Dilemma In Orthodontics. J Ayub Med Coll Abbottabad. 2018;30(1):16-21.	Excluded; mixed age group
739	Fink DF, Smith RJ. The duration of orthodontic treatment. Am J Orthod Dentofacial Orthop. 1992;102(1):45-51.	Excluded; mixed age group
740	Fleming PS, DiBiase AT, Lee RT. Randomized clinical trial of orthodontic treatment efficiency with self-ligating and conventional fixed orthodontic appliances. Am J Orthod Dentofacial Orthop. 2010;137(6):738-42.	Excluded; mixed age group
741	Gu J, Tang JS, Skulski B, Fields HW, Jr., Beck FM, Firestone AR, et al. Evaluation of Invisalign treatment effectiveness and efficiency compared with conventional fixed appliances using the Peer Assessment Rating index. Am J Orthod Dentofacial Orthop. 2017;151(2):259-66.	Excluded; mixed age group
742	Ho KH, Liao YF. Predictors of surgical-orthodontic treatment duration of unilateral impacted maxillary central incisors. Orthod Craniofac Res. 2011;14(3):175-80.	Excluded; mixed age group
743	Iseri H, Kisnisci R, Bzizi N, Tuz H. Rapid canine retraction and orthodontic treatment with dentoalveolar distraction osteogenesis. Am J Orthod Dentofacial Orthop. 2005;127(5):533-41; quiz 625.	Excluded; mixed age group
744	Janson G, Nakamura A, Barros SE, Bombonatti R, Chiqueto K. Efficiency of class i and class ii malocclusion treatment with four premolar extractions. Journal of Applied Oral Science. 2014;22(6):522-7.	Excluded; mixed age group
745	Jiang RP, McDonald JP, Fu MK. Root resorption before and after orthodontic treatment: a clinical study of contributory factors. Eur J Orthod. 2010;32(6):693-7.	Excluded; mixed age group
746	Kattner PF, Schneider BJ. Comparison of Roth appliance and standard edgewise appliance treatment results. Am J Orthod Dentofacial Orthop. 1993;103(1):24-32.	Excluded; mixed age group
747	Kocsis A, Seres L, Kocsis-Savanya G, Kovacs A. [Skeletal anchorage: use of miniscrews for impacted maxillary canine management]. Fogorv Sz. 2010;103(1):3-9.	Excluded; mixed age group
748	Kocsis A, Seres L. Orthodontic screws to extrude impacted maxillary canines. Journal of Orofacial Orthopedics-Fortschritte Der Kieferorthopadie. 2012;73(1):19-27.	Excluded; mixed age group
749	Kuftinec MM, Inman GO. A comparison of plain versus multilooped arch wires in stage I of Begg therapy. Am J Orthod. 1980;78(1):81-8.	Excluded; mixed age group
750	Lanteri V, Farronato G, Lanteri C, Caravita R, Cossellu G. The efficacy of orthodontic treatments for anterior crowding with Invisalign compared with fixed appliances using the Peer Assessment Rating Index. Quintessence Int. 2018;49(7):581-7.	Excluded; mixed age group
751	Leon-Salazar R, Janson G, Henriques JF, Leon-Salazar V. Influence of initial occlusal severity on time and efficiency of Class I malocclusion treatment carried out with and without premolar extractions. Dental Press J Orthod. 2014;19(4):38-49.	Excluded; mixed age group
752	Li QZ. 3M self-locking bracket versus straight wire bracket during orthodontic treatment. Chinese Journal of Tissue Engineering Research. 2015;19(25):4043-7.	Excluded; mixed age group
753	Li S, Chen J, Kula KS. Comparison of movement rate with different initial moment-to-force ratios. Am J Orthod Dentofacial Orthop. 2019;156(2):203-9.	Excluded; mixed age group
754	Li X, Xu ZR, Tang N, Ye C, Zhu XL, Zhou T, et al. Effect of intervention using a messaging app on compliance and duration of treatment in orthodontic patients. Clin Oral Investig. 2016;20(8):1849-59.	Excluded; mixed age group
755	Linge L, Linge BO. Patient characteristics and treatment variables associated with apical root	Excluded; mixed

	resorption during orthodontic treatment. Am J Orthod Dentofacial Orthop 1991;99(1):35-43.	age group
756	Maia NG, Normando D, Maia FA, Ferreira MA, do Socorro Costa Feitosa Alves M. Factors associated with long-term patient satisfaction. Angle Orthod. 2010;80(6):1155-8.	Excluded; mixed age group
757	Marques LS, Freitas Junior N, Pereira LJ, Ramos-Jorge ML. Quality of orthodontic treatment performed by orthodontists and general dentists. Angle Orthod. 2012;82(1):102-6.	Excluded; mixed age group
758	Martin J, Pancherz H. Mandibular incisor position changes in relation to amount of bite jumping during Herbst/multibracket appliance treatment: A radiographic-cephalometric study. Am J Orthod Dentofacial Orthop 2009;136(1):44-51.	Excluded; mixed age group
759	Mavragani M, Vergari A, Selliseth NJ, Boe OE, Wisth PL. A radiographic comparison of apical root resorption after orthodontic treatment with a standard edgewise and a straight-wire edgewise technique. Eur J Orthod. 2000;22(6):665-74.	Excluded; mixed age group
760	Motokawa M, Sasamoto T, Kaku M, Kawata T, Matsuda Y, Terao A, et al. Association between root resorption incident to orthodontic treatment and treatment factors. Eur J Orthod. 2012;34(3):350-6.	Excluded; mixed age group
761	Nahas AZ, Samara SA, Rastegar-Lari TA. Decrowding of lower anterior segment with and without photobiomodulation: a single center, randomized clinical trial. Lasers in Medical Science. 2017;32(1):129-35.	Excluded; mixed age group
762	Onyeaso CO, BeGole EA. Orthodontic treatment need in an accredited graduate orthodontic center in north america: a pilot study. J Contemp Dent Pract. 2006;7(2):87-94.	Excluded; mixed age group
763	Paolantonio M, di Girolamo G, Pedrazzoli V, di Murro C, Picciani C, Catamo G, et al. Occurrence of Actinobacillus actinomycetemcomitans in patients wearing orthodontic appliances. A cross-sectional study. J Clin Periodontol. 1996;23(2):112-8.	Excluded; mixed age group
764	Parrish LD, Roberts WE, Maupome G, Stewart KT, Bandy RW, Kula KS. The relationship between the ABO discrepancy index and treatment duration in a graduate orthodontic clinic. Angle Orthod. 2011;81(2):192-7.	Excluded; mixed age group
765	Penning EW, Peerlings RHJ, Govers JDM, Rischen RJ, Zinad K, Bronkhorst EM, et al. Orthodontics with Customized versus Noncustomized Appliances: A Randomized Controlled Clinical Trial. Journal of Dental Research. 2017;96(13):1498-504.	Excluded; mixed age group
766	Picanco GV, de Freitas KM, Cancado RH, Valarelli FP, Picanco PR, Feijao CP. Predisposing factors to severe external root resorption associated to orthodontic treatment. Dental Press J Orthod. 2013;18(1):110-20.	Excluded; mixed age group
767	Pinskaya YB, Hsieh TJ, Roberts WE, Hartsfield JK. Comprehensive clinical evaluation as an outcome assessment for a graduate orthodontics program. Am J Orthod Dentofacial Orthop. 2004;126(5):533-43.	Excluded; mixed age group
768	Pinto AS, Alves LS, Maltz M, Susin C, Zenkner JEA. Does the Duration of Fixed Orthodontic Treatment Affect Caries Activity among Adolescents and Young Adults? Caries Res. 2018;52(6):463-7.	Excluded; mixed age group
769	Pinto AS, Alves LS, Zenkner J, Zanatta FB, Maltz M. Gingival enlargement in orthodontic patients: Effect of treatment duration. Am J Orthod Dentofacial Orthop. 2017;152(4):477-82.	Excluded; mixed age group
770	Pseiner BC, Wunderlich A, Freudenthaler JW. Upper molar distalization with skeletally anchored TopJet appliance. J Orofac Orthop. 2014;75(1):42-50.	Excluded; mixed age group
771	Rakhshan V, Nateghian N, Ordoubazari M. Risk factors associated with external apical root resorption of the maxillary incisors: a 15-year retrospective study. Aust Orthod J. 2012;28(1):51-6.	Excluded; mixed age group
772	Richmond S, Andrews M, Roberts CT. The provision of orthodontic care in the general dental services of England and Wales: extraction patterns, treatment duration, appliance types and standards. Br J Orthod. 1993;20(4):345-50.	Excluded; mixed age group
773	Romanec CL, Georgeta Z. Surgical-orthodontic treatment of malocclusions through Begg technique. Rev Med Chir Soc Med Nat Iasi. 2012;116(4):1076-80.	Excluded; mixed age group
774	Salehi P, Torkan S, Gavareshki SR. Evaluating the effect of low energy laser irradiation on the rate of mandibular molar protraction in orthodontic patients. Journal of Research in Medical and Dental Science. 2016;4(3):228-32.	Excluded; mixed age group
775	Sameshima GT, Sinclair PM. Predicting and preventing root resorption: Part II. Treatment factors. Am J Orthod Dentofacial Orthop. 2001;119(5):511-5.	Excluded; mixed age group
776	Schubert M. The alignment of impacted and ectopic teeth using the Easy-Way-Coil (EWC®)System. Journal of Orofacial Orthopedics. 2008;69(3):213-26.	Excluded; mixed age group
777	Seres L, Kocsis A. Closure of severe skeletal anterior open bite with zygomatic anchorage. J Craniofac Surg. 2009;20(2):478-82.	Excluded; mixed age group
778	Sharab LY, Morford LA, Dempsey J, Falcao-Alencar G, Mason A, Jacobson E, et al. Genetic and treatment-related risk factors associated with external apical root resorption (EARR) concurrent with orthodontia. Orthod Craniofac Res. 2015;18 Suppl 1:71-82.	Excluded; mixed age group
779	Skidmore KJ, Brook KJ, Thomson WM, Harding WJ. Factors influencing treatment time in orthodontic patients. Am J Orthod Dentofacial Orthop. 2006;129(2):230-8.	Excluded; mixed age group
780	Strippoli J, Durand R, Schmittbuhl M, Rompre P, Voyer R, Chandad F, et al. Piezocorticision-assisted orthodontics: Efficiency, safety, and long-term evaluation of the inflammatory process. Am J Orthod Dentofacial Orthop. 2019;155(5):662-9.	Excluded; mixed age group
781	Taylor PJ, Kerr WJ, McColl JH. Factors associated with the standard and duration of orthodontic treatment. Br J Orthod. 1996;23(4):335-41.	Excluded; mixed age group
782	Vig PS, Weintraub JA, Brown C, Kowalski CJ. The duration of orthodontic treatment with and without extractions: a pilot study of five selected practices. Am J Orthod Dentofacial Orthop. 1990;97(1):45-51.	Excluded; mixed age group
783	von Bremen J, Streckbein EM, Ruf S. Changes in university orthodontic care over a period of 20 years : Patient characteristics, treatment quality, and treatment costs. J Orofac Orthop. 2017;78(4):321-9.	Excluded; mixed age group

784	Vu CQ, Roberts WE, Hartsfield JK, Jr., Ofner S. Treatment complexity index for assessing the relationship of treatment duration and outcomes in a graduate orthodontics clinic. Am J Orthod Dentofacial Orthop. 2008;133(1):9.e1-13.	Excluded; mixed age group
785	Wes Fleming J, Buschang PH, Kim KB, Oliver DR. Posttreatment occlusal variability among angle Class I nonextraction patients. Angle Orthod. 2008;78(4):625-30.	Excluded; mixed age group
786	Yassir YA, El-Angbawi AM, McIntyre GT, Revie GF, Bearn DR. A randomized clinical trial of the effectiveness of 0.018-inch and 0.022-inch slot orthodontic bracket systems: part 1-duration of treatment. Eur J Orthod. 2019;41(2):133-42.	Excluded; mixed age group
787	Zuccati G, Ghobadlu J, Nieri M, Clauser C. Factors associated with the duration of forced eruption of impacted maxillary canines: a retrospective study. Am J Orthod Dentofacial Orthop. 2006;130(3):349-56.	Excluded; mixed age group
788	Stucki N, Ingervall B. The use of the Jasper Jumper for the correction of Class II malocclusion in the young permanent dentition. Eur J Orthod. 1998;20(3):271-81.	Excluded; duration not assessed
789	Calheiros AdA, Miguel JAM, Moura PM, Almeida MAdO. Tratamento da má oclusão de Classe II de Angle em duas fases: avaliação da efetividade e eficácia por meio do índice PAR. Rev dent press ortodon ortopedi facial. 2008;13(1):43-53.	Excluded; different treatment for various age groups
790	Campbell CL, Roberts WE, Hartsfield Jr JK, Qi R. Treatment outcomes in a graduate orthodontic clinic for cases defined by the American Board of Orthodontics malocclusion categories. Am J Orthod Dentofacial Orthop 2007;132(6):822-9.	Excluded; different treatment for various age groups
791	Pietila I, Pietila T, Svedstrom-Oristo AL, Varrela J, Alanen P. Orthodontic treatment practices in Finnish municipal health centres with differing timing of treatment. Eur J Orthod. 2009;31(3):287-93.	Excluded; different treatment for various age groups
792	Nigul K, Jagomagi T. Factors related to apical root resorption of maxillary incisors in orthodontic patients. Stomatologija. 2006;8(3):76-9.	Excluded; missing data
793	Lee YJ, Lee TY. External root resorption during orthodontic treatment in root-filled teeth and contralateral teeth with vital pulp: A clinical study of contributing factors. Am J Orthod Dentofacial Orthop. 2016;149(1):84-91.	Excluded; only one adult patient
794	Bhattarai P, Shrestha RM. Comparative study of duration of orthodontic treatment among Nepalese adolescent and adult patients. Orthod J Nepal 2011;1(1):28-30.	Included
795	Dyer GS, Harris EF, Vaden JL. Age effects on orthodontic treatment: adolescents contrasted with adults. Am J Orthod Dentofacial Orthop. 1991 Dec;100(6):523-30.	Included
796	Furquim BD, Janson G, Cope LCC, Freitas KMS, Henriques JFC. Comparative effects of the Mandibular Protraction Appliance in adolescents and adults. Dental Press J Orthod. 2018;23(3):63-72.	Included
797	Harris EF, Baker WC. Loss of root length and crestal bone height before and during treatment in adolescent and adult orthodontic patients. Am J Orthod Dentofacial Orthop. 1990 Nov;98(5):463-9.	Included
798	Iancu Potrubacz M, Chimenti C, Marchione L, Tepedino M. Retrospective evaluation of treatment time and efficiency of a predictable cantilever system for orthodontic extrusion of impacted maxillary canines. Am J Orthod Dentofacial Orthop. 2018;154(1):55-64.	Included
799	Jiang F, Chen J, Kula K, Gu H, Du Y, Eckert G. Root resorptions associated with canine retraction treatment. Am J Orthod Dentofacial Orthop. 2017;152(3):348-54.	Included
800	Loke ST, Tan SY. Factors influencing duration of orthodontic treatment: A 12-year retrospective study. MDJ 2012;vol 34(2):16-30.	Included
801	Nienkemper M, Wilmes B, Pauls A, Yamaguchi S, Ludwig B, Drescher D. Treatment efficiency of mini-implant-borne distalization depending on age and second-molar eruption. J Orofac Orthop. 2014;75(2):118-32.	Included
802	Robb SI, Sadowsky C, Schneider BJ, BeGole EA. Effectiveness and duration of orthodontic treatment in adults and adolescents. Am J Orthod Dentofacial Orthop. 1998;114(4):383-6.	Included
803	Sachdeva RC, Aranha SL, Egan ME, Gross HT, Sachdeva NS, Currier GF, et al. Treatment time: SureSmile vs conventional. Orthodontics (Chic). 2012;13(1):72-85.	Included
804	Shim YS, Kim AH, An SY. A Study of Root Resorption in Upper and Lower Incisor in Patients following Orthodontic Treatment. Journal of Dental Hygiene Science. 2011;11(3):251-5.	Included
